# Emergence of multidrug-resistant *Mycobacterium tuberculosis* of the Beijing lineage in Portugal and Guinea-Bissau: a snapshot of moving clones by whole-genome sequencing

**DOI:** 10.1080/22221751.2020.1774425

**Published:** 2020-06-15

**Authors:** João Perdigão, Carla Silva, Fernando Maltez, Diana Machado, Anabela Miranda, Isabel Couto, Paulo Rabna, Paola Florez de Sessions, Jody Phelan, Arnab Pain, Ruth McNerney, Martin L. Hibberd, Igor Mokrousov, Taane G. Clark, Miguel Viveiros, Isabel Portugal

**Affiliations:** a iMed.ULisboa – Instituto de Investigação do Medicamento, Faculdade de Farmácia, Universidade de Lisboa, Lisboa, Portugal; b Serviço de Doenças Infecciosas, Hospital de Curry Cabral-CHLC, Lisboa, Portugal; c Global Health and Tropical Medicine, GHTM, Instituto de Higiene e Medicina Tropical, IHMT, Universidade Nova de Lisboa, UNL, Lisboa, Portugal; d National Mycobacterium Reference Laboratory, Instituto Nacional de Saúde Dr. Ricardo Jorge, Porto, Portugal; e Instituto Nacional de Saúde Pública/Projecto de Saúde de Bandim (INASA/PSB), Bissau, Guiné-Bissau; f Genomics Institute Singapore, Singapore; g London School of Hygiene & Tropical Medicine, London, UK; h Pathogen Genomics Laboratory, BESE Division, King Abdullah University of Science and Technology (KAUST), Thuwal, Kingdom of Saudi Arabia; i Department of Medicine, University of Cape Town, Cape Town, South Africa; j Laboratory of Molecular Epidemiology and Evolutionary Genetics, St Petersburg Pasteur Institute, St Petersburg, Russia

**Keywords:** MIRU-VNTR, tuberculosis, migration, Mycobacteria, Beijing, MtbC15-9 94-32, MtbC15-9 100-32

## Abstract

The Beijing genotype comprises a highly disseminated strain type that is frequently associated with multidrug resistant (MDR) tuberculosis (TB) and increased transmissibility but, countries such as Portugal and Guinea-Bissau fall outside the regions phylogeographically associated with this specific genotype. Nevertheless, recent data shows that this genotype might be gradually emerging in these two countries as an underlying cause of primary MDR-TB. Here, we describe the emergence of *Mycobacterium tuberculosis* Beijing strains associated with MDR-TB in Portugal and Guinea-Bissau demonstrating the presence of the well described superclusters 100-32 and 94-32 in Portugal and Guinea-Bissau, respectively. Genome-wide analysis and comparison with a global genomic dataset of *M. tuberculosis* Beijing strains, revealed the presence of two genomic clusters encompassing isolates from Portugal and Guinea-Bissau, GC1 (*n *= 121) and GC2 (*n *= 39), both of which bore SNP signatures compatible with the 100-32/B0/W148 and 94-32/Central Asia Outbreak clades, respectively. Moreover, GC2 encompasses a cross-border cluster between Portugal, Guinea-Bissau and Brazil thus supporting migration-associated introduction of MDR-TB and subsequent clonal expansion at the community-level. The comparison with global Beijing datasets demonstrates the global reach of the disease and its complex dissemination across multiple countries while in parallel there are clear microevolutionary trajectories towards extensively drug resistant TB.

## Introduction

Originally described by van Soolingen et al. in 1995, *Mycobacterium tuberculosis* Beijing lineage has shown a variable association with drug resistance coupled with a well-documented transmissibility and ability to cause tuberculosis (TB) outbreaks [1]. In the 1990s, Beijing strains became widely known as the underlying cause of a major New York multidrug-resistant (MDR)-TB outbreak among patients co-infected with the Human Immunodeficiency Virus (HIV) [2]. Other outbreaks involving drug resistant Beijing strains are often described in different regions throughout the globe [3,4]. This specific and widespread genotype was initially thought to have emerged in North-Central China more than one thousand years ago but, recent estimates point to Beijing lineage split approximately thirty thousand years ago in southern East Asia [5,6]. At the immunopathological level, the Beijing lineage has shown several potential selective advantages, particularly its “modern” sub-branch (i.e. strains harboring a IS*6110* at the NTF locus and *mutT4* codon 48 CGG > GGG substitution) which were shown in some studies to be associated with a reduced proinflammatory response and an increased virulence on mouse models of infection [7,8].

In Portugal, MDR-TB has been a major cause of public health concern particularly in the metropolitan area surrounding Lisbon. This concern is mainly due to two major monophyletic clades of the Latin American and Mediterranean (LAM) lineage (Lisboa3 and Q1), highly associated with MDR-TB, but also extensively drug resistant (XDR)-TB [9]. Despite its well-documented transmissibility and ability to cause TB outbreaks, Beijing strains are seldom isolated in the country [3,4,10].

Nevertheless, over the last 10 years we have been observing an increasing number of MDR-TB cases associated with Beijing strains (unpublished data). Recently, we developed CPLP-TB (http://cplp-tb.ff.ulisboa.pt), an online database aimed at tracking clones of *M. tuberculosis* expanding over Portuguese-speaking countries [11]. Presently, CPLP-TB contains nine and eight Beijing isolates from Portugal and Guinea-Bissau, respectively, with available 24-loci Mycobacterial Interspersed Repetitive Unit-Variable Number of Tandem Repeat (MIRU-VNTR) profiles. In Guinea-Bissau, the emergence of Beijing strains associated with MDR-TB was previously reported by us although the route driving the emergence and spread of such strains to this country was unclear [12].

Herein, we explore the source of the MDR-TB Beijing genotypes in both Portugal and Guinea-Bissau and unveil novel and previously uncharacterized routes for their emergence.

## Methods

### Clinical isolates and drug susceptibility testing

A total of seventeen *M. tuberculosis* clinical isolates belonging to the Beijing genotype as determined by spoligotyping were selected. From these, eight isolates were recovered from patients followed at the Hospital Raoul Follereau in Bissau, Guinea-Bissau, while the remaining nine isolates were obtained from patients at laboratories and hospitals across the Lisbon Health Region in Portugal between 2008 and 2014.

All isolates were subjected to susceptibility testing to all five first-line drugs by the BACTEC™ MGIT™ 960 System (Becton Dickinson Diagnostic Systems, Sparks, MD, USA) using standardized critical concentrations.

### Genotyping and cluster identification

Twenty-four-loci MIRU-VNTR typing was carried out by multiplex amplification procedure described by Supply et al [13]. Amplicon size determination was performed by capillary electrophoresis on an ABI 3130 Genetic Analyzer (Applied Biosystems^®^, Foster City, CA, USA) using a custom ROX-labelled molecular weight marker, MapMarker^®^1200 (Bioventures^®^, Murfreesboro, TN, USA), with 25 bands sized between 100 and 1200 bp.

Spoligotyping was performed by a single-tube multiplex PCR amplification of 43 spacer regions of the direct repeat (DR) locus using oligonucleotide primers DRa (5′-Biotin-GGTTTTGGGTCTGACGAC-3′) and DRb (5′-CCGAGAGGGGACGAAAC-3′) and 20 ng of genomic DNA. Amplicons were reverse hybridized on a membrane with amino-linked immobilized probes for each spacer as described previously by Kamerbeek et al. Detection was performed using the ECL^®^ Chemiluminescence Detection System (GE Healthcare^®^, Cleveland, OH, USA) as per the manufacturer’s instructions. Spoligotyping profiles were assigned to lineage, clade and shared international type (SIT) using the rules described in SITVITWEB and SITVIT2 international databases [10,14,15].

A comparative genotypic profile analysis was carried out including a total of 5482 isolates and 24-loci MIRU-VNTR profiles assembled from three large global studies, along with publicly available metadata [16–18]. Hierarchical clustering analysis was conducted in R statistical software using the *hclust* function and clusters defined as groups of two or more isolates sharing identical 24-loci MIRU-VNTR profiles. MtbC15-9 type classification was performed using the MIRU-VNTRplus online database [19]. The Hunter-Gaston index of diversity was computed as described previously [20].

### Whole genome sequencing and bioinformatic analysis

Whole genome sequencing (WGS) was carried out using an Illumina HiSeq 2500 paired-end (100/150 bp) platform. Single nucleotide polymorphisms (SNPs) were obtained by mapping raw sequence reads to the *M. tuberculosis* H37Rv genome (GenBank Ref. NC000962.3) using BWA-mem and by variant calling using the SAMtools/GATK software suites in established pipelines [9,21,22]. A comparison against publicly available sequence data was carried out for all sequenced isolates against 5296 Lineage 2 *M. tuberculosis* strains with Illumina short-read sequence data available on the European Nucleotide Archive (ENA) until July 2017 (see Supplementary Table S1 for full list of ENA accessions) and, for which SNPs were then called using the same bioinformatics pipeline as above. SNP sites or samples having an excess of 10% missing calls were removed from the analysis [21]. SNP sites within PE/PPE genes or those occurring at low mappability regions were also excluded from the analysis. The final dataset was composed of a total of 5180 isolates and 67,846 SNPs. Sequence data generated in this study is available on ENA under study accession ERP002611.

Genomic clustering was evaluated under both a five and twelve SNP distance threshold using R and the *ape* package [23]. A maximum-likelihood phylogenetic tree was constructed for the complete dataset using FastTree with a Generalised Time Reversible (GTR) model (Supplementary Figure S1) [24]. Additional maximum-likelihood phylogenetic trees were constructed using PhyML as implemented in Seaview for the genomic clusters found under a maximum distance of twelve SNPs. The phylogenetic context of the non-clustered isolates from Portugal or Guinea-Bissau was reconstructed by comparison with isolates within 100 SNPs. The best-fit nucleotide substitution models for each dataset were evaluated using the R package *phangorn*. The GTR model allowing across site rate variation and invariable sites was found to be the best-fit model for the GC1 (negative Log-likelihood: 99341.88), GC2 (negative Log-likelihood: 93879.79) and the GW000065 phylogenetic context (negative Log-likelihood: 100664.40); the GTR model allowing for invariable sites were found as the best-fit model for the PT000095 (negative Log-likelihood: 96291.71) and PT000025 (negative Log-likelihood: 94204.24) phylogenetic datasets. Tree visualization and annotation was performed using the *Interactive Tree of Life* tool [25]. A map showing the geographical dispersion of genomic clusters of interest was constructed using *Microreact* [26].

Minimum spanning trees (MSTs) were constructed using the goeBURST/Phyloviz software (available at http://online2.phyloviz.net) [27].

## Results

### Emergence of Beijing family MDR-TB in Portugal and Guinea-Bissau is associated with distinct superclusters

To understand the dynamics of emergence and transmission of MDR-TB associated with the Beijing genotype in Portugal and Guinea-Bissau, we analysed seventeen Beijing isolates from Portugal (*n *= 9) and Guinea-Bissau (*n *= 8), all of which recently characterized in the scope of the CPLP-TB database. The public health importance of these strains in both countries is noteworthy as 7/9 and 7/8 of these isolates in Portugal and Guinea-Bissau, respectively, are MDR-TB strains (Table 1). However, no links between the Portuguese and Guinean Beijing strains were found except at the 12-*loci* MIRU-VNTR set (Table 1, Supplementary Table S2). In Portugal, the nine Beijing strains yielded seven distinct profiles (two clusters of two isolates, each; and five non-clustered isolates) (Table 1). In Guinea-Bissau, five distinct profiles were observed encompassing two MIRU-VNTR clusters with two and three isolates, respectively, and three non-clustered isolates (Table 1). Regarding the clustered isolates, a classical epidemiological survey approach failed to find epidemiological links between patients, except for two patients (P16 and P17) which are of two siblings sharing the same household. Interestingly, both patients are Portuguese nationals for which no further TB contacts were identified and, neither had any prior history of travelling outside the country.

**Table 1.** Beijing strains isolated in Portugal and Guinea-Bissau with available 24-*loci* MIRU-VNTR profiles.

Isolate ID	Patient ID	Year of isolation	Country	Patient nationality	Drug resistance	WGS	SIT^a^/Clade	Genome-wide barcode	MtbC15-9 type	MIRU-VNTR cluster	SNP-based genomic cluster (12/5 SNPs)	Comments
GW000065	P1	2012	GW	Guinean	Susceptible	No	1/Beijing	nd	2061-88	NC	n.c.	12-MIRU type M33
GW000061	P2	2012	GW	Guinean	IRSEP	Yes	1/Beijing	2.2.1	9124-32	GW-01	GC2/GC2.1	12-MIRU type M2
GW000073	P3	2012	GW	Guinean	IRSEP	No	1/Beijing	nd	9124-32	GW-01	GC2/GC2.1	12-MIRU type M2
GW000063	P4	2012	GW	Guinean	IRSEP	No	1/Beijing	nd	94-32	GW-02	GC2/GC2.1	12-MIRU type M2
GW000064	P5	2012	GW	Guinean	IRSEP	No	1/Beijing	nd	94-32	GW-02	GC2/GC2.1	12-MIRU type M2
GW000072	P6	2012	GW	Guinean	IREP	No	1/Beijing	nd	94-32	GW-02	GC2/GC2.1	12-MIRU type M2
GW000068	P7	2012	GW	Guinean	IRS	No	1/Beijing	nd	95-88	NC	GC2/GC2.1	12-MIRU type M2
GW000066	P8	2012	GW	Guinean	IRSEP	No	1/Beijing	nd	97-387	NC	GC2/GC2.1	12-MIRU type M2
PT000443	P9	2007	PT	Unknown	ISEP	No	1/Beijing	nd	100-32	PT-02	nd	12-MIRU type M11
PT000013	P10	2008	PT	Moldavia	IRSEP	Yes	1/Beijing	2.2.1	100-32	PT-02	GC1/GC1.2	12-MIRU type M11
PT000025	P11	2009	PT	Unknown	IS	Yes	1/Beijing	2.2.1	2083-32	NC	n.c.	12-MIRU type M33
PT000095	P12	2009	PT	Portuguese	IR	Yes	1/Beijing	2.2.1	9387-32	NC	n.c.	12-MIRU type M8
PT000089	P13	2011	PT	Ukranyan	IRSEP	No	1/Beijing	nd	?-32	NC	nd	12-MIRU type M2
PT000242	P14	2011	PT	Brazillian	IRSEP	Yes	1/Beijing	2.2.1	17836-32	NC	n.c.	12-MIRU type M33
PT000074	P15	2014	PT	Ukranyan	IRSEP	No	1/Beijing	nd	?-32	NC	nd	12-MIRU type M166
PT000078	P16	2014	PT	Portuguese	IRSE	Yes	1/Beijing	2.2.1	4737-32	PT-06	GC1/GC1.1	Sibling to P17, married to prison guard (undiagnosed with TB); 12-MIRU type M1
PT000080	P17	2014	PT	Portuguese	IRSE	Yes	1/Beijing	2.2.1	4737-32	PT-06	GC1/GC1.1	Sibling to P16; 12-MIRU type M1

Further data (inc. MIRU-VNTR) available online at CPLP-TB: http://cplp-tb.ff.ulisboa.pt

^a^SIT, Shared International Type (available at SITVIT WEB, http://www.pasteur-guadeloupe.fr:8081/SITVIT_ONLINE/)

Except for cases directly linked with immigrants, which is not relevant in the Guinea-Bissau dataset since all patients were of Guinean nationality, the absence of additional transmission clusters with established epidemiological links render unknown the routes by which MDR-TB Beijing strains are being introduced. To identify links with other Beijing clusters, we compared the genotypic profiles of all seventeen clinical isolates included in this study against a global dataset of 5,482 isolates assembled from three large studies [16–18]. The comparison against this dataset yielded a total of 1,848 distinct profiles (Hunter-Gaston Index of Diversity, *h *= 0.9812). Four cross-border clusters, comprising six out of the nine Portuguese isolates, were identified matching MtbC15-9 type 100-32 (*n *= 302; 2 Portuguese [PT] isolates); 2083-32 (*n *= 18; 1 PT isolate); 4737–32 (*n *= 5; 2 PT isolates); and, 9387–32 (*n *= 3; 1 PT isolate) (Table 2). For Guinea-Bissau strains, two cross-border clusters were detected (*n *= 5) matching MtbC15-9 type 94-32 (*n *= 576; 3 Guinea-Bissau [GW] isolates) and 9124-32 (*n *= 6; 2 GW isolates) (Table 2).

**Table 2.** MIRU-VNTR (24-*loci*) obtained by comparison against a global Beijing dataset.

Cluster MtbC15-9	CPLP-TB Cluster	Total isolates in cluster	No. of isolates from Portugal	No. of isolates from Guinea-Bissau	Distribution per country of Isolation > 5% (%)^a^	No. drug resistant isolates in cluster (n) ^a^	Comment^a^
94-32	GW-02	576	0	3	Uzbekistan = 25; Kazakhstan = 25; Russia = 9.4; Germany = 5.7; Turkmenistan = 5.6	Susc = 56; non-MDR = 88; MDR = 317; XDR = 8; nd = 107	Russian/Asian Clone CC1 (RFLP-IS6110 A0 cluster)
100-32	PT-02	302	2	0	Russia = 28.5; Lithuania = 21.9; Germany = 7.9	Susc = 8; non-MDR = 26; MDR = 162; nd = 98	Russian B0/148
2083-32	NC	18	1	0	n.s.	non-MDR = 3; nd = 15	Highly prevalent in Vietnam (*n * = 11/18)
9124-32	GW-01	6	0	2	n.s	MDR = 5; nd = 1	Detected on Georgia (*n * = 1), Poland (*n * = 1) and Russia (*n * = 2)
4737-32	PT-06	5	2	0	n.s	MDR = 4; nd = 1	Armenia (*n *= 1), Lithuania (*n * = 1) and Russia (*n *= 1)
9387-32	NC	3	1	0	n.s	MDR = 1; nd = 2	Beyond Portugal, only detected in South Africa (*n *= 2)

^a^ Distribution according to global dataset assembled from Merker et al. [16], Skiba et al. [17] and Yin et al. [18].

n.s. – not significant due to the small number of isolates, see Comment column.

### WGS enables high-resolution clustering snapshots of cross-border superclusters and the detection of novel clustering between Portugal and Guinea-Bissau

To obtain a more resolved snapshot, we sequenced the genomes of 14 clinical isolates (Guinea-Bissau, *n *= 8; Portugal, *n *= 6) and compared them to 5,296 Lineage 2 strains available on ENA until July 2017 (Supplementary Table S1). The final dataset, after removal of low coverage samples and those having an excess of missing calls was composed of 5,181 isolates (including *M. tuberculosis* H37Rv as an outgroup strain) and 67,846 SNP sites. The global phylogenetic tree obtained for the entire dataset along with the examination of drug resistance genotypes showed a wide distribution and a high predominance of isolates showing a mutational profile compatible with MDR-TB (*n *= 1,447) and XDR-TB (*n *= 155) (Supplementary Figure S1). Moreover, the overall tree structure is consistent with multiple emergence of drug resistance and closely grouped clusters of MDR-TB.

**Figure 1.**
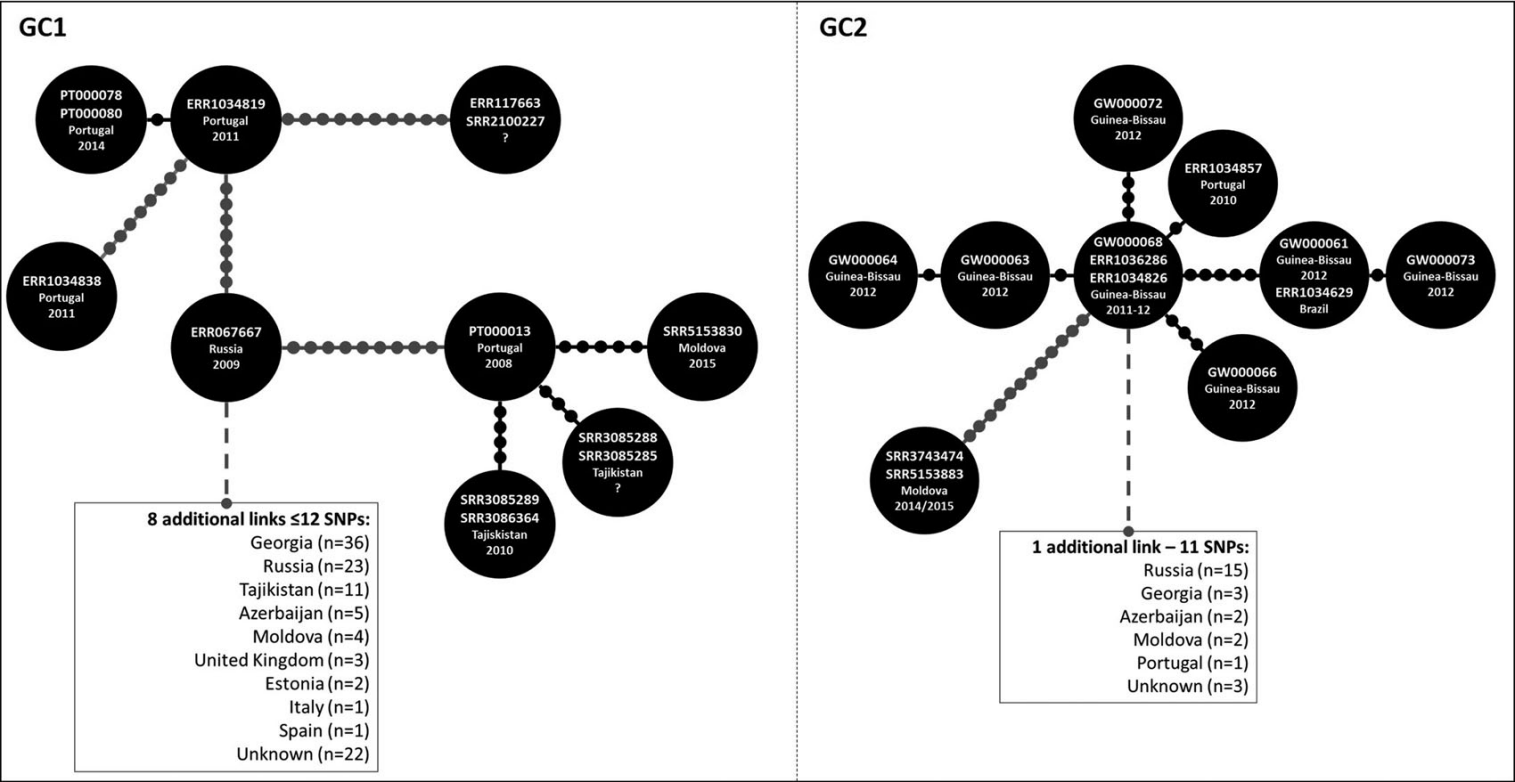
Genomic Clusters (GC) 1 and 2 comprising isolates characterized in the present study and herein defined as strains/nodes within 12 SNPs of distance between each other. Each GC is here partially represented as a Minimum Spanning Tree (MST) as to highlight nodes/strains closer to the analysed Beijing clinical isolates from Portugal and Guinea-Bissau (see Supplementary Figure S2 for full MSTs). Lines connecting each node represent a link of ≤12 SNPs where each dot represents the genetic distance corresponding to one SNP; lines depicted in black and grey represent distances ranging between 1–5 and 7–12 SNPs, respectively. GC1 comprises 121 isolates (displayed in a truncated form that highlights the positioning of Beijing strains isolated in Portugal) and includes isolates PT000078, PT000080 (both representing a known transmission case among the same household and involving two siblings, MtbC15-9 type 4737-32) and PT000013. PT000078 and PT000080 whose patients had no history of travel and of known TB contacts, were found to be in proximity with an imprisoned immigrant from Eastern Europe (ERR1034819), which is eight SNPs apart from another undetected immigrant from Eastern Europe in Portugal (ERR1034838). PT000013 pertains to a Moldovan immigrant in Portugal and is herein linked to an isolate originating from Moldova, seven years after this case has been detected in Portugal and is 3–4 SNPs apart of cases detected in Tajikistan. GC1 thus highlight the epidemiological impact and emergence of MDR-TB strains belonging to this GC that appear to originate from a complex transmission network that mainly spans across former Soviet states as well as its successful spread outside its endemic settings. GC2 depicts a cross-border cluster of previously unlinked cases between Portugal, Guinea-Bissau and Brazil that are also linked (≤12 SNPs) with isolates from former Soviet states. Overall, the high resolution offered by whole genome sequencing enabled the identification of previously epidemiologically unlinked cases and the identification of new routes for the spread of MDR-TB Beijing strains.

**Figure 2.**
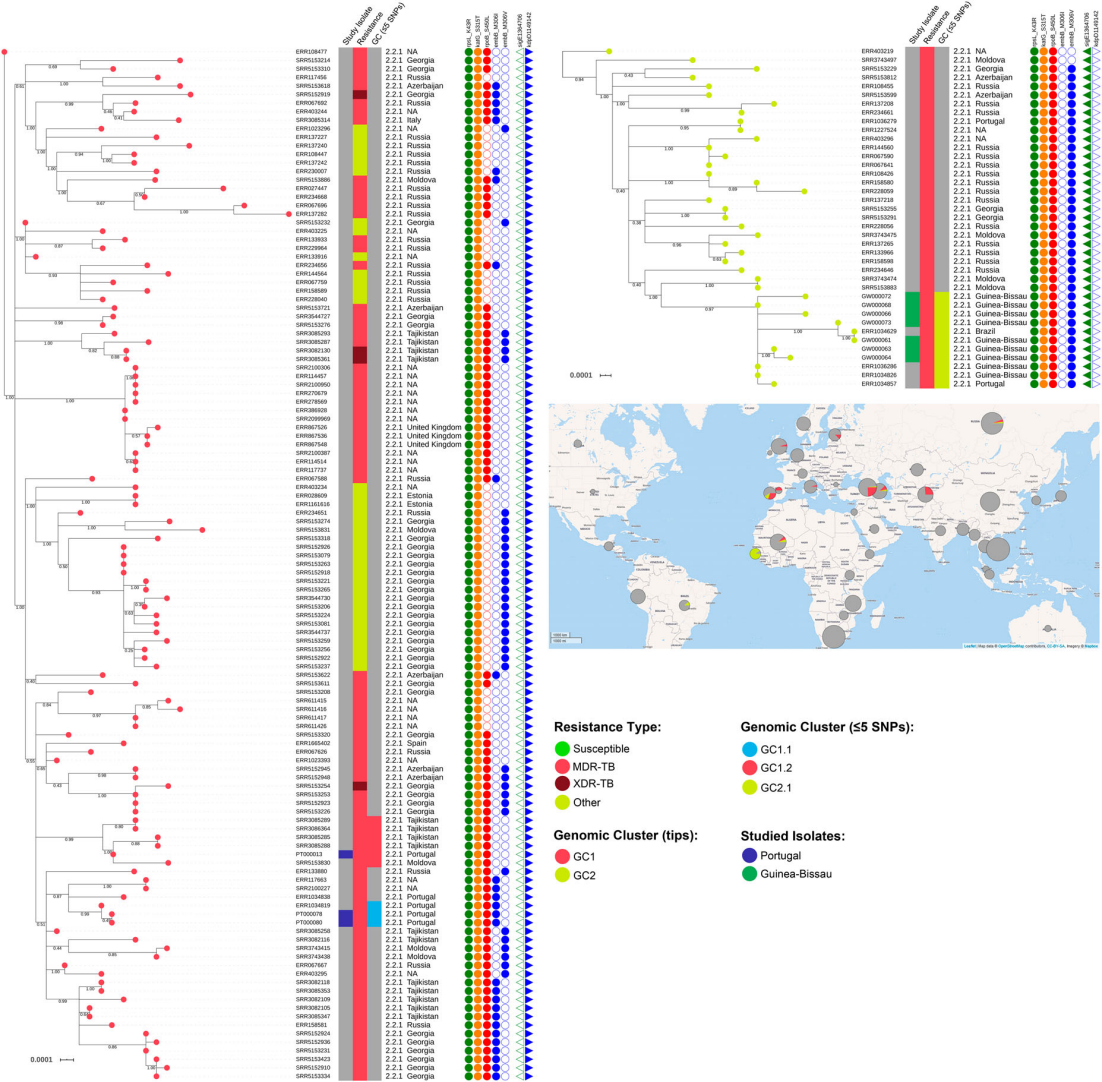
Maximum-likelihood based phylogenetic trees for GC1 and GC2encompassing 121 and 39 clinical isolates, respectively. The global phylogenetic tree is shown annotated with: tip colours for GC1 (red) and GC2 (yellow) isolates; a first inner colour strip highlighting isolates sequenced in this study from Portugal (blue) and Guinea-Bissau (green); a second colour strip highlighting genotypic based classification of isolates as susceptible (green), any drug resistance other than MDR-/XDR-TB (yellow), MDR-TB (red) and, XDR-TB (dark red); a third colour strip highlights sub-clusters at the five SNP distance threshold: GC1.1 (light blue), GC1.2 (red) and GC2.1 (yellow); sublineage and country of isolation; most common drug resistance associated mutations; and, presence of *kdpD* binucleotide deletion marker for the Russian B0/W148 (blue triangles) and the *sigE* silent mutation at codon 98 associated with type 94-32 (green triangles). A map illustrates the geographical dissemination of GC1 (red) and GC2 (yellow) across Asia, Europe, Africa and South America.

The genomic clustering analysis using a maximum distance of 12 SNPs [23] enabled the identification of two genomic clusters (GC1 and GC2) associated with nine out of the 14 initially sequenced strains (Table 1). GC2 (partially depicted in Figure 1 see Supplementary Figure S2 for the full MST) encompasses a total of 39 isolates including seven isolates sequenced in this study, all which from Guinea-Bissau. Interestingly, this cluster also comprises two additional isolates from Guinea-Bissau and another from Portugal (Figure 1). Further cluster discrimination using a five SNP threshold showed that all isolates from Guinea-Bissau herein studied plus two isolates also from Guinea-Bissau, one from Portugal and one from Brazil formed a more restricted genomic cluster (GC2.1) that comprised a monophyletic sub-branch within GC2 that is parallel to other sub-branches that encompass strains circulating mostly in Russia and on other neighbouring countries such as Georgia, Moldova or Azerbaijan. The topological structure of the tree is therefore suggestive of dissemination from these regions towards Guinea-Bissau, Portugal and Brazil. All GC2 harboured the *sigE* silent mutation at codon 98 (genome position 1364706, G > A), associated with type 94-32, which suggests possible descent from this cluster with concomitant divergence at the MIRU-VNTR *loci* (Figure 2) [28]. More specifically, all GC2 strains also bore the intergenic specific SNPs for the Central Asia Outbreak specific sub-branch of the 94-32 type [16].

The largest genome-wide-based cluster (GC1; partially depicted in Figure 1 see Supplementary Figure S2 for the full MST) was comprised of 121 *M. tuberculosis* Beijing isolates and included three isolates sequenced in this study (PT000078, PT000080 and PT000013). The first two clinical isolates belong to the two siblings (P16 and P17) with unknown TB contacts. Notably, WGS data showed these samples to be extremely close (one SNP difference) to a previously undetected isolate in Portugal three years before. An epidemiological investigation showed that the latter was obtained from an imprisoned Eastern European immigrant. No epidemiological link could be established between the imprisoned patient and the siblings, except for the fact that the husband of one of the siblings worked as a prison guard and was often responsible for transportation of imprisoned individuals; however this prison guard was never diagnosed with TB. The third isolate (PT000013) recovered from a Moldova immigrant in Portugal was also part of this large transmission network and genetically close (≤5 SNPs) to isolates originating from Moldova and Tajikistan. GC1 sub-clustering under a five SNP threshold confirmed the genomic clustering of the siblings with the imprisoned patient with the latter assuming a basal positioning to this monophyletic sub-cluster (GC1.1; Figure 2), while the PT000013 isolate was also found to be part of a monophyletic cluster (GC1.2; Figure 2) composed of five additional isolates from Tajikistan (*n *= 4) and Moldova (*n *= 1). These findings corroborate, on the one hand, that the dissemination of strains leading to PT000078 and PT000080 result from the clonal expansion of MDR-TB Beijing isolates in Portugal but that, on the other hand, strains belonging to this same cluster are thought to have disseminated from former Soviet states towards Eastern Europe and Western Europe, therein represented by Portugal. All strains in this GC were positive for the *kdpD* binucleotide deletion marker for the Russian B0/W148, part of the MIRU-VNTR 100-32 supercluster (Figure 2) [16,29]. Albeit belonging to MIRU-VNTR type 4737-32, PT00078 and PT00080 also bear this latter genetic marker highlighting its likely descent from 100-32 supercluster. In comparison to GC2, GC1 showed a more widespread distribution across Europe (Figure 2).

**Figure 3.**
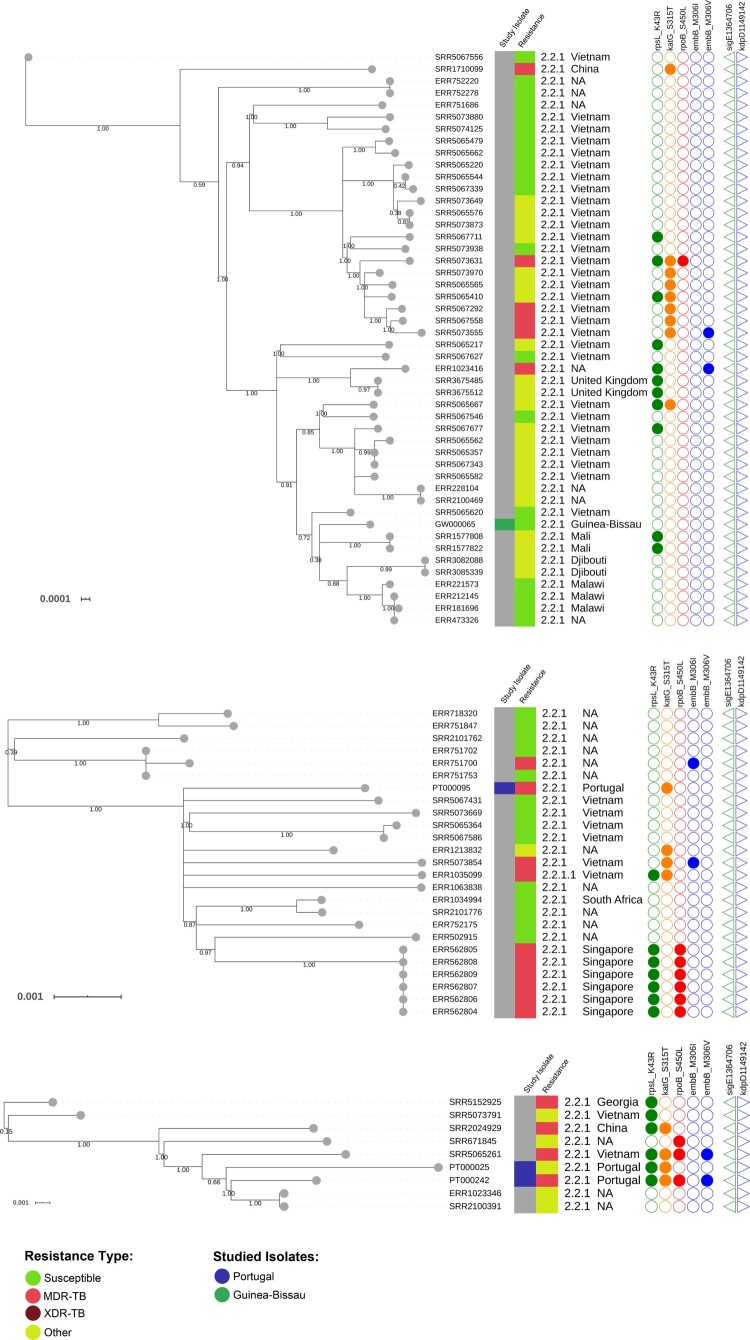
Maximum-likelihood based phylogenetic trees illustrating the phylogenetic context surrounding the non-clustered isolates GW000065, PT000095, PT000025 and PT000242. Three phylogenetic trees are shown annotated with: a first inner colour strip highlighting isolates sequenced in this study from Portugal (blue) and Guinea-Bissau (green); a second colour strip highlighting genotypic based classification of isolates as susceptible (green), any drug resistance other than MDR/XDR-TB (yellow), MDR-TB (red) and, XDR-TB (dark red); sublineage and country of isolation; most common drug resistance associated mutations; and, presence of *kdpD* binucleotide deletion marker for the Russian B0/W148 (blue triangles) and the *sigE* silent mutation at codon 98 associated with type 94-32 (green triangles).

For non-clustered isolates GW000065, PT000095, PT000025 and PT000242, the respective phylogenetic contexts were investigated by examining the topological structure of the phylogenetic trees constructed for isolates within 100 SNPs of these isolates (Figure 3). PT000242 and PT000025 were part of a sub-branch of sublineage 2.2.1 that is parallel to strains isolated in China and Vietnam. PT000095 is also phylogenetically positioned closer to strains circulating in Eastern hemisphere countries such as Vietnam and Singapore. Lastly, the GW000065 isolate from Guinea-Bissau is part of a sub-branch of sublineage 2.2.1 that appears to be disseminating in Africa and is parallel to other sub-branches mostly found in Vietnam which also suggests dissemination to Africa from the Eastern part of the globe (Figure 3).

### Molecular basis of resistance within GC1 and GC2 reveals multiple evolutionary trajectories towards resistance amplification and XDR-TB

We examined the molecular basis of resistance within the GCs herein detected (Table 3). All isolates in GC2 (*n *= 39) bore the classical *katG* S315T, *rpoB* S450L and *rpsL* K43R mutations associated with isoniazid (INH), rifampicin (RIF) and streptomycin (STR) resistance, respectively; *embB* M306V mutation was putatively associated with EMB resistance in 37 isolates, 11 of which corresponding to the Guinea-Bissau/Portugal/Brazil sub-branch of GC2 concomitantly showed the *embA* C-16G mutation; and, a D63A mutation on *pncA* potentially conferring PZA resistance was found in the latter eleven isolates. Furthermore, a frameshift *ethA* mutation (182delG) and a *thyX* promoter mutation (C-16T) were also detected among the same eleven GC2 isolates which, is therefore potentially linked with ETH and PAS resistance, respectively.

**Table 3.** Mutations detected across drug resistance associated genes in GC1 and GC2 isolates.

Drug^a^	Gene	GC1 (*n *= 121)	GC2 (*n *= 39)
INH	*ahpC*	G-48A (*n *= 1)	
	*inhA*	C-15T (*n *= 4); C-34T (*n *= 4); T-8G (*n *= 2); S94A (*n *= 1)	
	*katG*	S315T (*n *= 121)	S315T (*n *= 39)
RIF	*rpoB*	1349delCGinsTC (*n *= 18); 1365del3bp (*n *= 1); A286V (*n *= 1); A584G (*n *= 2); R827C (*n *= 5); D435G (*n *= 2); E761G (*n *= 1); G836S (*n *= 1); H835P (*n *= 1); I480V (*n *= 13); I491V (*n *= 4); L452P (*n *= 6); L731P (*n *= 12); P45S (*n *= 2); S450L (*n *= 78); V496A (*n *= 6)	S450L (*n *= 39)
STR	*gidB*	E92D (*n *= 121)	E92D (*n *= 39)
	*rpsL*	K43R (*n *= 121)	K43R (*n *= 39)
EMB	*embA*	C-12T (*n *= 1)	N722D (*n *= 1); C-16G (*n *= 11)
	*embB*	N296H (*n *= 4); D1024N (*n *= 1); D354A (*n *= 8); Q497R (*n *= 14); Q497K (*n *= 3); G406A (*n *= 5); G406D (*n *= 4); G406C (*n *= 1); G406S (*n *= 1); M306I (*n *= 28); M306V (*n *= 38); T643I (*n *= 1); Y319C (*n *= 2)	M306V (*n *= 37); Y319S (*n *= 1)
	*ubiA*	I179T (*n *= 4); W175G (*n *= 1)	T104A (*n *= 1)
PZA	*pncA*	260insCC (*n *= 1); 390delC (*n *= 2); D12A (*n *= 2); D136Y (*n *= 1); D63A (*n *= 6); Q141P (*n *= 17); G108R (*n *= 1); G17V (*n *= 1); G78D (*n *= 1); G97V (*n *= 1); H57R (*n *= 3); L116P (*n *= 1); L151S (*n *= 4); L172P (*n *= 1); L4S (*n *= 13); L4W (*n *= 2); M1I (*n *= 1); M1T (*n *= 1); T114P (*n *= 1); T168P (*n *= 1); T47A (*n *= 2); W68R (*n *= 1); W68G (*n *= 1); Y64D (*n *= 2); V139G (*n *= 1); V180A (*n *= 6); V7G (*n *= 2); A-11G (*n *= 2); G-13T (*n *= 2)	188delG (*n *= 4); 352insG (*n *= 2); D63A (*n *= 11); G132S (*n *= 2); H51Y (*n *= 1); I133T (*n *= 1); I6S (*n *= 2); L156P (*n *= 1); K96T (*n *= 1); V139L (*n *= 1); V155G (*n *= 1); V163E (*n *= 1)
FQ	*gyrA*	280del5bpins5bp (*n *= 1); 281del4bpins4bp (*n *= 13); A90V (*n *= 5); E21Q (*n *= 121); G668D (*n *= 121); H52Y (*n *= 1); S447F (*n *= 4)	280del5bpins5bp (*n *= 1); 281del4bpins4bp (*n *= 7); E21Q (*n *= 39); G668D (*n *= 39)
SLID	*eis*	C-12T (*n *= 3); C-14T (*n *= 3); G-10A (*n *= 10); G-37T (*n *= 19)	C-12T (*n *= 1); C-14T (*n *= 4); G-10A (*n *= 1)
	*rrs*	A1401G (*n *= 16)	A1401G (*n *= 8)
ETH	*ethA*	1012delA (*n *= 7); 113delT (*n *= 9); 511delT (*n *= 3); 705delA (*n *= 2); 828delC (*n *= 2); 887delA (*n *= 6); L440P (*n *= 5); F64V (*n *= 1); W256Ter (*n *= 2); Y140Ter (*n *= 2); Y461C (*n *= 1)	182delG (*n *= 11); 945delG (*n *= 1); R207G (*n *= 2); Q254P (*n *= 1); Q347Ter (*n *= 2); L62R (*n *= 4); T314I (*n *= 9); W391Ter (*n *= 2)
	*inhA*	C-15T (*n *= 4); C-34T (*n *= 4); T-8G (*n *= 2); S94A (*n *= 1)	
DCS	*alr*	S22L (*n *= 1)	
PAS	*folC*	I43T (*n *= 2); S150G (*n *= 1)	E40G (*n *= 1)
	*thyA*	654delC (*n *= 1)	P253A (*n *= 12)
	*thyX*	C-16T (*n *= 1); C-19G (*n *= 1)	C-16T (*n *= 11)

^a^Drug abbreviations: INH, isoniazid; RIF, rifampicin; STR, streptomycin; EMB, ethambutol; PZA, pyrazinamide; FQ, fluoroquinolones; SLID, second-line injectable drugs; ETH, ethionamide; DCS, D-cycloserine; PAS, para-aminosalicylic acid.

Contrarily to GC2, GC1 (*n *= 121) exhibited a remarkably higher mutational diversity associated with drug resistance. Nevertheless, two mutations were common across all GC1 isolates: *rpsL* K43R and *katG* S315T. Considering the three GC1 isolates sequenced in this study, all possessed a *rpoB* S450L mutation but diverged on the mutational profile of other loci: the siblings P16 and P17 isolates’ both showed *embB* M306I, *rpoB* V496A, and a single nucleotide deletion on *ethA* (887delA); the remaining isolate possessed a *pncA* D63A mutation. Globally, GC1 isolates showed 98 distinct mutations on drug resistance associated loci, and 4/121 showed mutational profiles compatible with XDR-TB (Figure 2). Associated with PZA resistance, the *pncA* gene showed the highest mutational diversity: 29 mutations, two of which occurring on the promoter region (Table 3).

Comparing the mutations found across GC1 and GC2 isolates to the complete Beijing dataset assembled for this study (Supplementary Table S3) it is noteworthy that: (i) the *ethA* 182delG and 887delA in eleven GC2 and in six GC1 isolates, respectively, were not found in any other Beijing isolates; (ii) the *rpoB* V496A mutation was detected only in the GC1 sub-branch encompassing PT000078 and PT000080; and (iii) the *pncA* D63A was only detected in 17 isolates across the entire dataset, eleven of which from GC2 and six from GC1. In contrast, *rpoB* S450L, *katG* S315T and *rpsL* K43R mutations were highly prevalent as these were detected in 1246 (77.8%), 1,392 (86.9%) and 778 (48.6%) MDR isolates, respectively, and thereby confirming these as the most prevalent mutations in drug resistant isolates, particularly in former Soviet states [16]. Moreover, the *thyX* C-16T and *embA* C-16G mutation were well represented in eleven GC2 (28.2%) isolates whereas across MDR-TB and in the entire dataset these mutations were detected only in 62 (3.8%) MDR-TB isolates (total: 72 [1.4%] across all isolates) for the *thyX* C-16T mutation and, in 15 (0.9%) MDR-TB isolates (total: 17 [0.3%] across all isolates) for the *embA* C-16G mutation.

## Discussion

The increasing rates of MDR-TB and XDR-TB are a major roadblock to the WHO TB Elimination goals. Despite having the *M. tuberculosis* genetic diversity characterized to different extents, Portugal and Guinea-Bissau present different healthcare systems and challenges regarding TB control. A common challenge pertains the introduction and transmission of non-endemic strains such as the Beijing family with no study previously attempting to establish epidemiological links between these countries and investigate the routes by which these strains may be introduced in the current epidemiological scenario of each country.

Most Beijing strains detected in Portugal and Guinea-Bissau were in fact not clustered using classical typing methods. This latter observation suggests recent emergence of these strains, particularly in Portugal. A higher degree of clustered Beijing isolates was observed for Guinea-Bissau therefore suggesting ongoing transmission of Beijing strains but the route by which these strains were imported into Guinea-Bissau is unclear as all patients were of Guinean nationality. It is known that the Beijing is the predominant genotype across Central, Eastern, South-Eastern Asia and North Eurasia and, that it has been detected in Northern, Eastern and Southern Africa [10,15]. This data suggests that MDR-TB Beijing strains are now also emerging in West African countries such as Guinea-Bissau.

To understand and place the origin of Beijing isolates in Portugal and Guinea-Bissau in a broader epidemiological context, we compiled a global dataset of 5,482 clinical isolates. Isolates belonging to superclusters 100-32 and 94-32 were detected in Portugal and Guinea-Bissau, respectively. Supercluster 100-32 (more specifically, its major B0/W148 variant) is highly associated with MDR-TB in Russia [29] and is often isolated from immigrants in Europe, whereas supercluster 94-32 (Central Asian/Russian clone), second to 100-32 in Russia and less associated with MDR-TB, is more prevalent in former Soviet Central Asia [17]. Beyond these, the presence of types 2083-32, 4737-32 and 9387-32 in Portugal suggests other possible sources of Beijing strains. The presence of type 9124-32 in Guinea-Bissau is also suggestive of dissemination from Russia or eastern European countries, where it is more prevalent. All clusters but type 9387-32 had been previously associated with drug resistance which stresses the ability of MDR-TB Beijing strains to expand beyond its endemic settings. This initial analysis based on cluster dispersion is indicative of different possible sources for the Beijing strains in these settings. One possible link between Guinea-Bissau and Russia lies in the close bilateral relations that once existed between former Soviet Union and Guinea-Bissau which promoted scholarships for Guinean students and military training in the Soviet Union. However, the main limitation of this MIRU-VNTR based analysis lies in its poor discriminatory power when compared to WGS and poor ability to infer on the directionality of the transmission dynamics. The fact that all MIRU-VNTR clusters were defined based on 24-loci along with a stringent definition of cluster based on completely similar profiles does limit the discordance between methods [30]. Further discriminatory power could have been obtained through the inclusion of 1980, 3232, 3820 and 4120 hypervariable VNTR loci since the standardized 24-loci MIRU-VNTR typing set alone is in many circumstances unable to resolve closely related clones such as the 94-32 and 100-32 [31,32]. While the standard 24-loci set enabled the discriminatory of the Beijing isolates in a non-endemic setting such as Portugal, the inexistence of global datasets with hypervariable allelic also hampers global comparison using this classical typing method.

Nevertheless, WGS did allow for a more resolved scenario in a global context and, enabled the identification of two genomic clusters, herein named GC1 and GC2, that included isolates sequenced in this study. In fact, GC2 encompassed a cross-border clustering event between Portugal, Guinea-Bissau and Brazil, with the number of immigrants and arrivals from Guinea-Bissau to Portugal alerting for a previously unrecognized source of MDR-TB Beijing strains in this country [11]. Moreover, the phylogenetic context of GC2 further demonstrated that emergence of MDR-TB Beijing in Guinea-Bissau is likely driven from strains imported from Eastern Europe or former Soviet countries to Guinea-Bissau and later mobilized to other Portuguese-speaking countries. Interestingly, GC2 is herein shown to be part of the Central Asia Outbreak clade which is a specific sub-branch of the Central Asia clade, more associated with MDR-TB and extensive geographic dissemination beyond its place of origin [33]. Contrarily to GC2, GC1 shows a widespread distribution across Central Asia and Europe and, has been detected in Portugal as a result of two separate and independent dissemination events: (i) one involving four cases, two of which detected in the present study, that given the apparent genetic distances suggest clonal expansion already in Portugal but whose original geographical source is unknown; and (ii) a single case phylogenetically nested within strains isolated in Tajikistan and Moldova that may have spread into Europe via other former Soviet countries. In fact, GC1 appears to be dominated by strains circulating within former Soviet states, comprising a reservoir of MDR-TB Beijing strains emerging in Europe and likely to be part of Clade B/East-Europe2/B0-W148 cluster [6,16,34]. The mutational diversity associated with GC1 corroborates its extensive dissemination and multiple evolutionary trajectories leading to resistance amplification towards XDR-TB from a common ancestor already resistant to INH and STR. This observation is also corroborated by the detection of different XDR-TB and pre-XDR-TB Beijing strains in Spain among sex workers, one of which (BJR1/ERR1665402) herein found to belong to GC1 [35]. While the latter did not disseminate in the community, GC1 secondary cases occurring in Portugal are herein observed and, stress the importance of screening and prompt access to TB care for immigrants [36].

The phylogenetic context of the non-clustered isolates suggests that the three non-clustered isolates from Portugal are representative of strains that appear to circulate mostly in East Asian countries such as China and Vietnam. Also, the same can be said for the single Guinea-Bissau non-clustered isolate although this isolate appears to represent an entire clade disseminating across Africa but more deeply rooted across Asian countries. The migrant population in Guinea-Bissau (*n *= 1933, according to the last population census in 2009) is mostly comprised of immigrants from Western African countries which can pose as vehicle for the dissemination of these strains but the migration profile of the country is poorly characterized [37]. Portugal on the other hand has observed a steep increase in the foreign population with residency status originating from Asia: from 24,269, in 2007, to 54,508 legal residents in 2017 [38]. The emergence of non-clustered Beijing strains in Portugal can therefore result from the increasing international migratory flow from high-prevalence settings for Beijing strains other than Eastern European countries.

In conclusion, the present study corroborates the emergence of MDR-TB Beijing strains in Portugal and Guinea-Bissau linked with former Soviet Countries, as well as previously unidentified cross-border clustering between Guinea-Bissau and Portugal concerning the epidemiology of MDR-TB Beijing strains. The latter is of special concern as the Lusophone migratory system may constitute an additional gateway for intercontinental dissemination of MDR-TB Beijing strains to countries such as Brazil or Angola, that appear to have a very low prevalence of Beijing strains. Also, we stress the role of WGS as a high-resolution typing tool that can assist the tracking of international relevant clones and its transmission dynamics.

## Supplementary Material

Supplementary_TableS1.pdf

## References

[1] van Soolingen D, Qian L, de Haas PE, et al. Predominance of a single genotype of Mycobacterium tuberculosis in countries of east Asia. J Clin Microbiol. 1995;33(12):3234–3238. 8586708 10.1128/jcm.33.12.3234-3238.1995 PMC228679

[2] Bifani PJ, Plikaytis BB, Kapur V, et al. Origin and interstate spread of a New York City multidrug-resistant Mycobacterium tuberculosis clone family. JAMA. 1996;275(6):452–457. 8627966

[3] Gurjav U, Erkhembayar B, Burneebaatar B, et al. Transmission of multi-drug resistant tuberculosis in Mongolia is driven by Beijing strains of Mycobacterium tuberculosis resistant to all first-line drugs. Tuberculosis (Edinb). 2016;101:49–53. 27865397 10.1016/j.tube.2016.07.010

[4] Marais BJ, Mlambo CK, Rastogi N, et al. Epidemic spread of multidrug-resistant tuberculosis in Johannesburg, South Africa. J Clin Microbiol. 2013;51(6):1818–1825. 23554196 10.1128/JCM.00200-13 PMC3716102

[5] Mokrousov I. Genetic geography of Mycobacterium tuberculosis Beijing genotype: a multifacet mirror of human history? Infect Genet Evol. 2008;8(6):777–785. 18691674 10.1016/j.meegid.2008.07.003

[6] Luo T, Comas I, Luo D, et al. Southern East Asian origin and coexpansion of Mycobacterium tuberculosis Beijing family with Han Chinese. Proc Natl Acad Sci U S A. 2015;112(26):8136–8141. 26080405 10.1073/pnas.1424063112 PMC4491734

[7] Ribeiro SC, Gomes LL, Amaral EP, et al. Mycobacterium tuberculosis strains of the modern sublineage of the Beijing family are more likely to display increased virulence than strains of the ancient sublineage. J Clin Microbiol. 2014;52(7):2615–2624. 24829250 10.1128/JCM.00498-14 PMC4097719

[8] van Laarhoven A, Mandemakers JJ, Kleinnijenhuis J, et al. Low induction of proinflammatory cytokines parallels evolutionary success of modern strains within the Mycobacterium tuberculosis Beijing genotype. Infect Immun. 2013;81(10):3750–3756. 23897611 10.1128/IAI.00282-13 PMC3811744

[9] Perdigao J, Silva H, Machado D, et al. Unraveling Mycobacterium tuberculosis genomic diversity and evolution in Lisbon, Portugal, a highly drug resistant setting. BMC Genomics. 2014;15(991). 10.1186/1471-2164-15-991 PMC4289236 25407810

[10] Demay C, Liens B, Burguiere T, et al. SITVITWEB – a publicly available international multimarker database for studying Mycobacterium tuberculosis genetic diversity and molecular epidemiology. Infect Genet Evol. 2012;12(4):755–766. 22365971 10.1016/j.meegid.2012.02.004

[11] Perdigao J, Silva C, Diniz J, et al. Clonal expansion across the seas as seen through CPLP-TB database: A joint effort in cataloguing Mycobacterium tuberculosis genetic diversity in Portuguese-speaking countries. Infect Genet Evol. 2019;72:44–58. 29559379 10.1016/j.meegid.2018.03.011 PMC6598853

[12] Rabna P, Ramos J, Ponce G, et al. Direct detection by the Xpert MTB/RIF Assay and Characterization of Multi and Poly drug-resistant tuberculosis in Guinea-Bissau, West Africa. PLoS ONE. 2015;10(5):e0127536. 26017968 10.1371/journal.pone.0127536 PMC4446334

[13] Supply P, Allix C, Lesjean S, et al. Proposal for standardization of optimized mycobacterial interspersed repetitive unit-variable-number tandem repeat typing of Mycobacterium tuberculosis. J Clin Microbiol. 2006;44(12):4498–4510. 17005759 10.1128/JCM.01392-06 PMC1698431

[14] Couvin D, Rastogi N. Tuberculosis - A global emergency: Tools and methods to monitor, understand, and control the epidemic with specific example of the Beijing lineage. Tuberculosis (Edinb). 2015;95(Suppl 1):S177–S189. 25797613 10.1016/j.tube.2015.02.023

[15] Couvin D, David A, Zozio T, et al. Macro-geographical specificities of the prevailing tuberculosis epidemic as seen through SITVIT2, an updated version of the Mycobacterium tuberculosis genotyping database. Infect Genet Evol. 2019;72:31–43. 30593925 10.1016/j.meegid.2018.12.030

[16] Merker M, Blin C, Mona S, et al. Evolutionary history and global spread of the Mycobacterium tuberculosis Beijing lineage. Nat Genet. 2015;47(3):242–249. 25599400 10.1038/ng.3195 PMC11044984

[17] Skiba Y, Mokrousov I, Ismagulova G, et al. Molecular snapshot of Mycobacterium tuberculosis population in Kazakhstan: a country-wide study. Tuberculosis (Edinb). 2015;95(5):538–546. 26076582 10.1016/j.tube.2015.04.012

[18] Yin QQ, Liu HC, Jiao WW, et al. Evolutionary history and ongoing transmission of phylogenetic Sublineages of Mycobacterium tuberculosis Beijing genotype in China. Sci Rep. 2016;6(34353). 10.1038/srep34353 PMC5041183 27681182

[19] Allix-Beguec C, Harmsen D, Weniger T, et al. Evaluation and strategy for use of MIRU-VNTRplus, a multifunctional database for online analysis of genotyping data and phylogenetic identification of Mycobacterium tuberculosis complex isolates. J Clin Microbiol. 2008;46(8):2692–2699. 18550737 10.1128/JCM.00540-08 PMC2519508

[20] Hunter PR, Gaston MA. Numerical index of the discriminatory ability of typing systems: an application of Simpson’s index of diversity. J Clin Microbiol. 1988;26(11):2465–2466. 3069867 10.1128/jcm.26.11.2465-2466.1988 PMC266921

[21] Coll F, Phelan J, Hill-Cawthorne GA, et al. Genome-wide analysis of multi- and extensively drug-resistant Mycobacterium tuberculosis. Nat Genet. 2018;50(2):307–316. 29358649 10.1038/s41588-017-0029-0

[22] Coll F, McNerney R, Guerra-Assuncao JA, et al. A robust SNP barcode for typing Mycobacterium tuberculosis complex strains. Nat Commun. 2014;5:4812. 25176035 10.1038/ncomms5812 PMC4166679

[23] Walker TM, Ip CL, Harrell RH, et al. Whole-genome sequencing to delineate Mycobacterium tuberculosis outbreaks: a retrospective observational study. Lancet Infect Dis. 2013;13(2):137–146. 23158499 10.1016/S1473-3099(12)70277-3 PMC3556524

[24] Price MN, Dehal PS, Arkin AP. FastTree 2 – approximately maximum-likelihood trees for large alignments. PLoS ONE. 2010;5(3):e9490. 20224823 10.1371/journal.pone.0009490 PMC2835736

[25] Letunic I, Bork P. Interactive tree Of Life (iTOL) v4: recent updates and new developments. Nucleic Acids Res. 2019;47(W1):W256–W2W9. 30931475 10.1093/nar/gkz239 PMC6602468

[26] Argimon S, Abudahab K, Goater RJE, et al. Microreact: visualizing and sharing data for genomic epidemiology and phylogeography. Microb Genom. 2016;2(11):e000093. 28348833 10.1099/mgen.0.000093 PMC5320705

[27] Nascimento M, Sousa A, Ramirez M, et al. PHYLOViz 2.0: providing scalable data integration and visualization for multiple phylogenetic inference methods. Bioinformatics. 2017;33(1):128–129. 27605102 10.1093/bioinformatics/btw582

[28] Mokrousov I, Chernyaeva E, Vyazovaya A, et al. Rapid Assay for detection of the epidemiologically Important Central Asian/Russian strain of the Mycobacterium tuberculosis Beijing genotype. J Clin Microbiol. 2018;56(2):e01551–17. 29142045 10.1128/JCM.01551-17 PMC5786733

[29] Mokrousov I, Narvskaya O, Vyazovaya A, et al. Russian “successful” clone B0/W148 of Mycobacterium tuberculosis Beijing genotype: a multiplex PCR assay for rapid detection and global screening. J Clin Microbiol. 2012;50(11):3757–3759. 22933595 10.1128/JCM.02001-12 PMC3486266

[30] Nikolayevskyy V, Kranzer K, Niemann S, et al. Whole genome sequencing of Mycobacterium tuberculosis for detection of recent transmission and tracing outbreaks: A systematic review. Tuberculosis (Edinb). 2016;98:77–85. 27156621 10.1016/j.tube.2016.02.009

[31] Allix-Beguec C, Wahl C, Hanekom M, et al. Proposal of a consensus set of hypervariable mycobacterial interspersed repetitive-unit-variable-number tandem-repeat loci for subtyping of Mycobacterium tuberculosis Beijing isolates. J Clin Microbiol. 2014;52(1):164–172. 24172154 10.1128/JCM.02519-13 PMC3911419

[32] Murase Y, Izumi K, Ohkado A, et al. Prediction of Local transmission of Mycobacterium tuberculosis isolates of a predominantly beijing lineage by use of a variable-number tandem-repeat typing method incorporating a consensus set of hypervariable loci. J Clin Microbiol. 2018;56(1):e01016–17. 29046413 10.1128/JCM.01016-17 PMC5744219

[33] Shitikov E, Vyazovaya A, Malakhova M, et al. Simple Assay for detection of the Central Asia Outbreak clade of the Mycobacterium tuberculosis Beijing genotype. J Clin Microbiol. 2019;57(7):e00215–19. 31043465 10.1128/JCM.00215-19 PMC6595453

[34] Casali N, Nikolayevskyy V, Balabanova Y, et al. Microevolution of extensively drug-resistant tuberculosis in Russia. Genome Res. 2012;22(4):735–745. 22294518 10.1101/gr.128678.111 PMC3317155

[35] Perez-Lago L, Martinez-Lirola M, Garcia S, et al. Urgent Implementation in a Hospital setting of a Strategy To Rule Out secondary cases Caused by imported extensively drug-resistant Mycobacterium tuberculosis strains at Diagnosis. J Clin Microbiol. 2016;54(12):2969–2974. 27682128 10.1128/JCM.01718-16 PMC5121387

[36] Pereira C, Gomes P, Taveira R, et al. Insights on the Mycobacterium tuberculosis population structure associated with migrants from Portuguese-speaking countries over a three-year period in Greater Lisbon, Portugal: Implications at the public health level. Infect Genet Evol. 2019;71:159–165. 30928606 10.1016/j.meegid.2019.03.025

[37] Instituto Nacional de Estatística. Recenseamento Geral da População e Habitação – Guiné Bissau. Bissau: Instituto Nacional de Estatística; 2009.

[38] Gabinete de Estratégia e Economia. População Estrangeira Residente em Portugal. Lisboa: Ministério da Economia; 2018.

