# Supplementary material for: Emergence of multidrug-resistant Mycobacterium tuberculosis of the Beijing lineage in Portugal and Guinea-Bissau: a snapshot of moving clones by whole-genome sequencing
Source: Emerg Microbes Infect. 2020 Jun 15;9(1):1342–53. doi: 10.1080/22221751.2020.1774425 (PMC7473242; doi:10.1080/22221751.2020.1774425)
Supplement: Supplementary_TableS1.pdf [file TEMI_A_1774425_SM3565.pdf]

Supplementary Table S1 - ENA Run Accessions (n=5 167) used in this study to track putative epidemiologically linked isolates to Beijing/Lineage 2 strains collected in Portugal and Guinea-Bissau. Origin, collection date and strain was obtained from ENA by parsing associated sample XML files. Resistance type and (Sub)Lineage data were inferred in this study by mapping raw read data to *M. tuberculosis* H37Rv genome (GenBank Accession NC000962.3) and examining for the occurrence/distribution of SNPs and Indels previously associated with specific lineage barcodes and drug resistance [1,2].

Accession	Origin	Collection Year	Resistance Type	(Sub)Lineage
DRR019435	Japan	2007	Other	2.2.1
DRR019436	Japan	2009	Other	2.2.1
DRR019437	Japan	2010	Other	2.2.1
DRR019438	Japan	2006	Other	2.2.1
DRR019439	Japan	2005	Other	2.2.1
DRR019440	Japan	2005	Other	2.2.1
DRR019441	Japan	2009	Other	2.2.1
DRR019442	Japan	2010	Other	2.2.1
DRR019443	Japan	2009	Other	2.2.1
DRR019444	Japan	2008	Other	2.2.1
DRR041783	NA	2004	Susceptible	2.2.1
DRR041784	NA	2005	Susceptible	2.2.1
DRR041785	NA	2010	Susceptible	2.2.1
DRR041786	NA	2010	Susceptible	2.2.1
DRR041787	NA	2011	Susceptible	2.2.1
DRR041788	NA	2011	Susceptible	2.2.1
DRR041789	NA	2011	Susceptible	2.2.1
ERR017783	Russia	2008	MDR	2.2.1
ERR017784	Russia	2008	MDR	2.2.1
ERR017786	Russia	2008	MDR	2.2.1
ERR017787	United Kingdom	2006	Susceptible	2.2.1
ERR017788	Estonia	NA	MDR	2.2.1
ERR019564	Russia	2008	MDR	2.2.1
ERR019565	United Kingdom	2005	Susceptible	2.2.1.1
ERR019569	United Kingdom	2005	Susceptible	2.2.1
ERR019570	United Kingdom	2005	Susceptible	2.2.1
ERR019571	United Kingdom	2005	Susceptible	2.2.1
ERR019572	Russia	2008	Other	2.2.1
ERR019573	United Kingdom	2005	Susceptible	2.2.1.1
ERR019574	United Kingdom	2005	Other	2.2.1
ERR019575	Russia	2008	MDR	2.2.1
ERR024350	NA	NA	MDR	2.2.1
ERR024351	NA	NA	MDR	2.2.1
ERR025439	Netherlands	1998	Other	2.2.2
ERR025839	United Kingdom	2008	Susceptible	2.2.1
ERR025840	United Kingdom	2008	Susceptible	2.2.1
ERR025876	NA	NA	Susceptible	2.2.1.2
ERR025881	NA	NA	Susceptible	2.1
ERR025890	NA	NA	Susceptible	2.2.1
ERR026473	Netherlands	1995	Susceptible	2.2.1
ERR026474	Netherlands	2003	Susceptible	2.2.1
ERR026477	Netherlands	2008	Susceptible	2.2.1
ERR026478	Netherlands	2004	Susceptible	2.2.1
ERR026479	Netherlands	2005	Susceptible	2.2.1
ERR026480	Netherlands	2007	Susceptible	2.2.1.1
ERR026481	Netherlands	2000	Susceptible	2.2.1.1
ERR026482	Netherlands	2002	Susceptible	2.2.1.1
ERR027445	Russia	2008	Susceptible	2.2.1
ERR027446	Russia	2008	MDR	2.2.1
ERR027447	Russia	2008	MDR	2.2.1
ERR027448	Russia	2008	MDR	2.2.1
ERR027449	Russia	2008	Susceptible	2.2.1
ERR027450	Russia	2008	MDR	2.2.1
ERR027451	Russia	2008	MDR	2.2.1

ERR027452	Estonia	NA	MDR	2.2.1
ERR027454	Russia	2008	Other	2.2.1
ERR027455	Russia	2008	MDR	2.2.1
ERR027456	Russia	2008	MDR	2.2.1
ERR027458	Russia	2008	MDR	2.2.1
ERR027459	Russia	2008	Susceptible	2.2.1
ERR027462	United Kingdom	2005	Susceptible	2.2.1
ERR027463	NA	NA	XDR	2.2.1
ERR027468	NA	NA	MDR	2.2.1
ERR028358	NA	NA	Susceptible	2.2.2
ERR028359	NA	NA	Susceptible	2.2.1
ERR028367	NA	NA	Susceptible	2.2.1
ERR028368	NA	NA	Susceptible	2.2.1
ERR028607	Estonia	2005	MDR	2.2.1
ERR028609	Estonia	2005	Other	2.2.1
ERR028611	Netherlands	1994	Other	2.2.1
ERR028619	Estonia	2005	MDR	2.2.1
ERR028623	Netherlands	1998	Other	2.2.2
ERR029203	South Africa	1900/2013	Susceptible	2.2.1.1
ERR029205	Netherlands	2008	Susceptible	2.2.1
ERR029206	Netherlands	2005	Susceptible	2.2.1
ERR029207	Netherlands	2005	Susceptible	2.2.1
ERR029209	Estonia	2005	MDR	2.2.1
ERR029210	Netherlands	2004	MDR	2.2.1
ERR031449	NA	NA	Susceptible	2.2.1
ERR031453	NA	NA	Susceptible	2.2.1
ERR031454	NA	NA	Susceptible	2.2.1
ERR031467	NA	NA	Susceptible	2.2.1
ERR031468	NA	NA	Susceptible	2.2.1.1
ERR031469	NA	NA	Susceptible	2.1
ERR031470	NA	NA	Susceptible	2.2.1.1
ERR031471	NA	NA	Susceptible	2.2.1
ERR031475	NA	NA	Susceptible	2.1
ERR031476	NA	NA	Susceptible	2.2.1
ERR031481	NA	NA	Susceptible	2.2.1.1
ERR031486	NA	NA	Other	2.2.1.2
ERR031492	NA	NA	Susceptible	2.2.1
ERR036186	Malawi	1995	Susceptible	2.2.1
ERR036235	Malawi	1997	Susceptible	2.2.1
ERR037551	Malawi	1999	Susceptible	2.2.1
ERR038746	Uganda	01-03-2005	MDR	2.2.1
ERR046938	United Kingdom	2010	Susceptible	2.2.1
ERR046947	United Kingdom	2010	Susceptible	2.2.1
ERR046949	United Kingdom	2010	Susceptible	2.2.1
ERR046963	United Kingdom	2010	Susceptible	2.2.1
ERR046966	United Kingdom	2010	Susceptible	2.2.1
ERR046967	United Kingdom	2010	Susceptible	2.2.1
ERR047003	United Kingdom	2007	Other	2.2.1
ERR067576	Russia	2008	MDR	2.2.1
ERR067578	Russia	2008	MDR	2.2.1
ERR067580	Russia	2008	Susceptible	2.2.1
ERR067581	Russia	2008	MDR	2.2.1
ERR067582	Russia	2008	Other	2.2.1
ERR067583	Russia	2008	MDR	2.2.1
ERR067584	Russia	2008	MDR	2.2.1
ERR067586	Russia	2008	Other	2.2.1
ERR067587	Russia	2008	MDR	2.2.1
ERR067588	Russia	2008	MDR	2.2.1
ERR067589	Russia	2008	Other	2.2.1
ERR067590	Russia	2008	MDR	2.2.1
ERR067592	Russia	2008	MDR	2.2.1

ERR067593	Russia	2008	MDR	2.2.1
ERR067594	Russia	2008	Susceptible	2.2.1
ERR067595	Russia	2009	Susceptible	2.2.1
ERR067598	Russia	2009	MDR	2.2.1
ERR067600	Russia	2008	Other	2.2.1
ERR067604	Russia	2008	Susceptible	2.2.1
ERR067605	Russia	2008	MDR	2.2.1
ERR067608	Russia	2008	Other	2.2.1
ERR067610	Russia	2008	Other	2.2.1
ERR067611	Russia	2008	MDR	2.2.1
ERR067613	Russia	2008	MDR	2.2.1
ERR067618	Russia	2008	MDR	2.2.1
ERR067619	Russia	2008	XDR	2.2.1
ERR067620	Russia	2008	MDR	2.2.1
ERR067621	Russia	2008	MDR	2.2.1
ERR067622	Russia	2008	MDR	2.2.1
ERR067623	Russia	2008	XDR	2.2.1
ERR067625	Russia	2008	MDR	2.2.1
ERR067626	Russia	2008	MDR	2.2.1
ERR067627	Russia	2008	MDR	2.2.1
ERR067628	NA	NA	MDR	2.2.1
ERR067630	Russia	2008	MDR	2.2.1
ERR067631	Russia	2008	MDR	2.2.1
ERR067635	Russia	2009	MDR	2.2.1
ERR067637	Russia	2009	Susceptible	2.2.1
ERR067638	Russia	2009	Other	2.2.1
ERR067639	Russia	2009	Other	2.2.1
ERR067641	Russia	2009	MDR	2.2.1
ERR067643	Russia	2009	MDR	2.2.1
ERR067644	Russia	2009	XDR	2.2.1
ERR067646	Russia	2009	Susceptible	2.2.1
ERR067647	Russia	2009	MDR	2.2.1
ERR067648	Russia	2009	Susceptible	2.2.1
ERR067649	Russia	2009	MDR	2.2.1
ERR067650	Russia	2009	MDR	2.2.1
ERR067651	Russia	2009	MDR	2.2.1
ERR067652	Russia	2009	MDR	2.2.1
ERR067653	Russia	2009	MDR	2.2.1
ERR067654	Russia	2009	MDR	2.2.1
ERR067655	Russia	2009	Susceptible	2.2.1
ERR067656	Russia	2009	MDR	2.2.1
ERR067659	Russia	2009	MDR	2.2.1
ERR067660	Russia	2009	MDR	2.2.1
ERR067662	Russia	2009	MDR	2.2.1
ERR067663	Russia	2009	MDR	2.2.1
ERR067665	Russia	2009	MDR	2.2.1
ERR067667	Russia	2009	MDR	2.2.1
ERR067668	Russia	2009	MDR	2.2.1
ERR067671	Russia	2009	MDR	2.2.1
ERR067672	Russia	2009	MDR	2.2.1
ERR067673	Russia	2009	MDR	2.2.1
ERR067674	Russia	2009	MDR	2.2.1
ERR067686	Russia	2009	Susceptible	2.2.1
ERR067687	Russia	2009	MDR	2.2.1
ERR067688	Russia	2009	Susceptible	2.2.1
ERR067689	Russia	2009	Susceptible	2.2.1
ERR067692	Russia	2008	MDR	2.2.1
ERR067694	Russia	2008	MDR	2.2.1
ERR067695	Russia	2008	MDR	2.2.1
ERR067696	Russia	2008	MDR	2.2.1
ERR067698	Russia	2009	MDR	2.2.1

ERR067702	NA	NA	Susceptible	2.2.1
ERR067704	Russia	2009	MDR	2.2.1
ERR067705	Russia	2009	MDR	2.2.1
ERR067707	Russia	2009	MDR	2.2.1
ERR067709	Russia	2009	MDR	2.2.1
ERR067710	Russia	2009	MDR	2.2.1
ERR067711	Russia	2009	MDR	2.2.1
ERR067713	Russia	2009	Susceptible	2.2.1
ERR067714	Russia	2009	MDR	2.2.1
ERR067715	Russia	2009	Other	2.2.1
ERR067717	Russia	2009	MDR	2.2.1
ERR067718	Russia	2008	MDR	2.2.1
ERR067720	Russia	2008	MDR	2.2.1
ERR067721	Russia	2008	MDR	2.2.1
ERR067722	Russia	2008	MDR	2.2.1
ERR067723	Russia	2008	MDR	2.2.1
ERR067724	Russia	2008	MDR	2.2.1
ERR067736	Russia	2008	Susceptible	2.2.1
ERR067739	Russia	2008	MDR	2.2.1
ERR067740	Russia	2008	Susceptible	2.2.1
ERR067741	Russia	2008	MDR	2.2.1
ERR067742	Russia	2008	MDR	2.2.1
ERR067743	Russia	2008	Susceptible	2.2.1
ERR067744	Russia	2008	XDR	2.2.1
ERR067745	Russia	2008	MDR	2.2.1
ERR067746	Russia	2008	Susceptible	2.2.1
ERR067749	NA	NA	MDR	2.2.1
ERR067750	Russia	2008	MDR	2.2.1
ERR067754	Russia	2008	MDR	2.2.1
ERR067755	Russia	2008	MDR	2.2.1
ERR067758	Russia	2008	MDR	2.2.1
ERR067759	Russia	2008	Other	2.2.1
ERR067760	Russia	2008	MDR	2.2.1
ERR067761	Russia	2008	XDR	2.2.1
ERR067762	Russia	2008	MDR	2.2.1
ERR067764	NA	NA	MDR	2
ERR067765	Russia	2008	MDR	2.2.1
ERR067766	Russia	2008	Susceptible	2.2.1
ERR072027	United Kingdom	2007	Other	2.2.1
ERR1023296	NA	NA	Other	2.2.1
ERR1023297	NA	NA	MDR	2.2.1
ERR1023301	NA	NA	MDR	2.2.1
ERR1023346	NA	NA	Other	2.2.1
ERR1023353	NA	NA	MDR	2.2.1
ERR1023356	NA	NA	Susceptible	2.2.1
ERR1023366	NA	NA	Other	2.2.1
ERR1023392	NA	NA	Susceptible	2.2.1
ERR1023393	NA	NA	MDR	2.2.1
ERR1023395	NA	NA	Other	2.2.1
ERR1023396	NA	NA	Other	2.2.1
ERR1023400	NA	NA	MDR	2.2.1
ERR1023401	NA	NA	MDR	2.2.1.1
ERR1023403	NA	NA	MDR	2.2.1
ERR1023404	NA	NA	MDR	2.2.1
ERR1023406	NA	NA	Susceptible	2.2.1
ERR1023407	NA	NA	Other	2.2.1
ERR1023409	NA	NA	MDR	2.2.1
ERR1023413	NA	NA	Other	2.2.1
ERR1023415	NA	NA	MDR	2.2.1
ERR1023416	NA	NA	MDR	2.2.1
ERR1023419	NA	NA	Susceptible	2.2.1.1

ERR1023422	NA	NA	Susceptible	2.2.1.1
ERR1023423	NA	NA	Susceptible	2.2.1
ERR1023427	NA	NA	Susceptible	2.2.1
ERR1023428	NA	NA	Susceptible	2.2.1.1
ERR1023436	NA	NA	Susceptible	2.2.1
ERR1023438	NA	NA	Susceptible	2.2.1
ERR1023439	NA	NA	Susceptible	2.2.1.1
ERR1023442	NA	NA	MDR	2.2.1.1
ERR1023445	NA	NA	Susceptible	2.2.2
ERR1023448	NA	NA	Susceptible	2.2.1
ERR1023449	NA	NA	Susceptible	2.2.1.1
ERR1023454	NA	NA	MDR	2.2.2
ERR1023456	NA	NA	Susceptible	2.2.1
ERR1023458	NA	NA	Susceptible	2.2.1
ERR1023459	NA	NA	Susceptible	2.2.1.1
ERR1023461	NA	NA	Susceptible	2.2.1.1
ERR1023462	NA	NA	Susceptible	2.2.1
ERR1023464	NA	NA	Susceptible	2.2.1
ERR1023465	NA	NA	XDR	2.2.1
ERR1023468	NA	NA	Susceptible	2.2.1
ERR1023475	NA	NA	Other	2.2.1.1
ERR1023476	NA	NA	Susceptible	2.2.1
ERR1023480	NA	NA	XDR	2.2.2
ERR1023481	NA	NA	Susceptible	2.2.1.1
ERR1023484	NA	NA	Susceptible	2.2.1
ERR1023486	NA	NA	Susceptible	2.2.1
ERR1023487	NA	NA	Susceptible	2.2.1
ERR1023491	NA	NA	Susceptible	2.2.1
ERR1023496	NA	NA	Susceptible	2.2.1
ERR1023498	NA	NA	Susceptible	2.2.1.1
ERR1023499	NA	NA	Susceptible	2.2.1.1
ERR1023500	NA	NA	Susceptible	2.2.1.1
ERR1023501	NA	NA	MDR	2.2.1.1
ERR1023507	NA	NA	Susceptible	2.2.1.1
ERR1023510	NA	NA	MDR	2.2.2
ERR1023512	NA	NA	Other	2.2.1.1
ERR1023516	NA	NA	MDR	2.2.1.1
ERR1023517	NA	NA	Other	2.2.1
ERR1023518	NA	NA	Susceptible	2.2.1.1
ERR1023519	NA	NA	Other	2.2.1
ERR1023520	NA	NA	MDR	2.2.1
ERR1023521	NA	NA	MDR	2.2.1
ERR1023523	NA	NA	MDR	2.2.1
ERR1023524	NA	NA	MDR	2.2.2
ERR1023528	NA	NA	Susceptible	2.2.1
ERR1023530	NA	NA	MDR	2.2.2
ERR1023531	NA	NA	Other	2.2.1
ERR1034629	Brazil	NA	MDR	2.2.1
ERR1034637	Brazil	NA	MDR	2.2.1
ERR1034638	Brazil	NA	MDR	2.2.1
ERR1034640	Brazil	NA	XDR	2.2.1
ERR1034642	Brazil	NA	MDR	2.2.1
ERR1034643	Brazil	NA	MDR	2.2.2
ERR1034644	Brazil	NA	MDR	2.2.1
ERR1034645	Brazil	NA	MDR	2.2.2
ERR1034648	Brazil	NA	MDR	2.2.2
ERR1034666	Bulgaria	NA	MDR	2.2.1
ERR1034698	India	NA	Other	2.2.1
ERR1034717	Peru	NA	MDR	2.2.1
ERR1034738	Peru	NA	MDR	2.2.1
ERR1034751	Peru	NA	Other	2.2.1

ERR1034754	Peru	NA	MDR	2.2.1
ERR1034787	Peru	NA	Other	2.2.1
ERR1034801	Peru	NA	Susceptible	2.2.1
ERR1034819	Portugal	NA	MDR	2.2.1
ERR1034826	Guinea-Bissau	NA	MDR	2.2.1
ERR1034838	Portugal	NA	MDR	2.2.1
ERR1034857	Portugal	NA	MDR	2.2.1
ERR1034861	Portugal	NA	MDR	2.2.1
ERR1034876	Portugal	NA	MDR	2.2.1
ERR1034877	Portugal	NA	MDR	2.2.1
ERR1034880	Portugal	NA	MDR	2.2.1
ERR1034893	Portugal	NA	MDR	2.2.1
ERR1034912	South Africa	NA	Susceptible	2.2.1
ERR1034914	South Africa	NA	Susceptible	2.2.1
ERR1034918	South Africa	NA	Susceptible	2.2.1
ERR1034955	South Africa	NA	Susceptible	2.2.1.1
ERR1034958	South Africa	NA	Susceptible	2.2.1
ERR1034977	South Africa	NA	MDR	2.2.2
ERR1034982	South Africa	NA	MDR	2.2.1.1
ERR1034985	South Africa	NA	Susceptible	2.2.1.1
ERR1034986	South Africa	NA	MDR	2.2.2
ERR1034994	South Africa	NA	Susceptible	2.2.1
ERR1034995	South Africa	NA	XDR	2.2.2
ERR1035007	South Africa	NA	MDR	2.2.1
ERR1035008	South Africa	NA	Susceptible	2.2.1
ERR1035010	South Africa	NA	MDR	2.2.1
ERR1035012	South Africa	NA	MDR	2.2.1
ERR1035013	South Africa	NA	MDR	2.2.1
ERR1035014	South Africa	NA	MDR	2.2.2
ERR1035015	South Africa	NA	MDR	2.2.1
ERR1035019	South Africa	NA	Other	2.2.2
ERR1035022	South Africa	NA	Other	2.2.1.1
ERR1035023	South Africa	NA	MDR	2.2.1
ERR1035024	South Africa	NA	MDR	2.2.2
ERR1035025	South Africa	NA	MDR	2.2.2
ERR1035027	South Africa	NA	MDR	2.2.1
ERR1035031	South Africa	NA	MDR	2.2.2
ERR1035035	South Africa	NA	Other	2.2.1.1
ERR1035036	South Africa	NA	MDR	2.2.2
ERR1035038	South Africa	NA	XDR	2.2.2
ERR1035040	South Africa	NA	Other	2.2.1.1
ERR1035041	South Africa	NA	MDR	2.2.2
ERR1035042	South Africa	NA	MDR	2.2.2
ERR1035043	South Africa	NA	MDR	2.2.1
ERR1035044	South Africa	NA	Other	2.2.1.1
ERR1035045	South Africa	NA	Other	2.2.1.1
ERR1035047	South Africa	NA	Other	2.2.1
ERR1035050	South Africa	NA	Other	2.2.1
ERR1035055	South Africa	NA	Other	2.2.1.1
ERR1035058	South Africa	NA	Other	2.2.1
ERR1035060	South Africa	NA	Other	2.2.1.1
ERR1035064	South Africa	NA	Other	2.2.1
ERR1035065	South Africa	NA	MDR	2.2.1
ERR1035067	South Africa	NA	Other	2.2.1.1
ERR1035069	South Africa	NA	MDR	2.2.1
ERR1035073	South Africa	NA	MDR	2.2.1.1
ERR1035074	South Africa	NA	MDR	2.2.1
ERR1035075	South Africa	NA	MDR	2.2.1
ERR1035076	South Africa	NA	MDR	2.2.1
ERR1035077	South Africa	NA	MDR	2.2.2
ERR1035078	South Africa	NA	MDR	2.2.1

ERR1035079	South Africa	NA	MDR	2.2.1
ERR1035081	South Africa	NA	MDR	2.2.1
ERR1035082	South Africa	NA	MDR	2.2.1
ERR1035084	South Africa	NA	MDR	2.2.1
ERR1035085	South Africa	NA	MDR	2.2.1
ERR1035086	Vietnam	NA	MDR	2.2.1
ERR1035087	Vietnam	NA	MDR	2.2.1
ERR1035088	Vietnam	NA	MDR	2.2.1.1
ERR1035089	Vietnam	NA	Other	2.2.1
ERR1035090	Vietnam	NA	MDR	2.2.1
ERR1035094	Vietnam	NA	Other	2.2.1
ERR1035097	Vietnam	NA	MDR	2.1
ERR1035098	Vietnam	NA	MDR	2.2.1
ERR1035099	Vietnam	NA	MDR	2.2.1.1
ERR1035100	Vietnam	NA	Other	2.2.1.1
ERR1035102	Vietnam	NA	MDR	2.2.1
ERR1035103	Vietnam	NA	MDR	2.2.1
ERR1035107	Vietnam	NA	MDR	2.2.1
ERR1035114	Vietnam	NA	Susceptible	2.2.1
ERR1035117	Vietnam	NA	Susceptible	2.2.1
ERR1035124	Vietnam	NA	Susceptible	2.2.1
ERR1035127	Vietnam	NA	Other	2.2.1
ERR1035129	Vietnam	NA	Susceptible	2.2.1
ERR1035132	Vietnam	NA	Susceptible	2.2.1
ERR1035133	Vietnam	NA	Other	2.2.1.1
ERR1035135	Vietnam	NA	Other	2.2.2
ERR1035137	Saudi Arabia	NA	Susceptible	2.2.1
ERR1035139	Saudi Arabia	NA	MDR	2.2.1
ERR1035140	Saudi Arabia	NA	Susceptible	2.2.1.1
ERR1035159	Saudi Arabia	NA	Susceptible	2.2.2
ERR1035169	Saudi Arabia	NA	MDR	2.2.1
ERR1035882	NA	NA	Other	2.2.1
ERR1036279	Portugal	NA	MDR	2.2.1
ERR1036286	Guinea-Bissau	NA	MDR	2.2.1
ERR1036287	Portugal	NA	MDR	2.2.1
ERR1036292	Portugal	NA	Other	2.2.1
ERR1063838	NA	NA	Susceptible	2.2.1
ERR1063841	NA	NA	MDR	2.2.1
ERR1063845	NA	NA	Susceptible	2.2.1
ERR1063867	NA	NA	Susceptible	2.2.1
ERR1063891	NA	NA	Susceptible	2.2.1
ERR108128	South Africa	2008/2014	Susceptible	2.2.1.1
ERR108129	South Africa	2008/2014	Susceptible	2.2.1.1
ERR108130	South Africa	2008/2014	Susceptible	2.2.1.1
ERR108143	South Africa	2008/2014	Susceptible	2.2.1.1
ERR108147	South Africa	2008/2014	Susceptible	2.2.1
ERR108148	South Africa	2008/2014	Susceptible	2.2.1
ERR108151	South Africa	2008/2014	Susceptible	2.2.1.1
ERR108153	South Africa	2008/2014	Susceptible	2.2.1.1
ERR108155	South Africa	2008/2014	Susceptible	2.2.1.1
ERR108156	South Africa	2008/2014	Susceptible	2.2.1.1
ERR108159	South Africa	2008/2014	Susceptible	2.2.1.1
ERR108160	South Africa	2008/2014	Susceptible	2.2.1.1
ERR108161	South Africa	2008/2014	Susceptible	2.2.1
ERR108162	South Africa	2008/2014	Susceptible	2.2.1
ERR108165	South Africa	2008/2014	Susceptible	2.2.1.1
ERR108166	South Africa	2008/2014	Susceptible	2.2.1.1
ERR108171	South Africa	2008/2014	Susceptible	2.2.1.1
ERR108172	South Africa	2008/2014	Susceptible	2.2.1.1
ERR108173	South Africa	2008/2014	Susceptible	2.2.1.1
ERR108174	South Africa	2008/2014	Susceptible	2.2.1.1

ERR108177	South Africa	2008/2014	Susceptible	2.2.1
ERR108178	South Africa	2008/2014	Susceptible	2.2.1
ERR108181	South Africa	2008/2014	Susceptible	2.2.1
ERR108182	South Africa	2008/2014	Susceptible	2.2.1
ERR108183	South Africa	2008/2014	Susceptible	2.2.1.1
ERR108184	South Africa	2008/2014	Susceptible	2.2.1.1
ERR108420	Russia	2009	MDR	2.2.1
ERR108421	Russia	2009	MDR	2.2.1
ERR108423	Russia	2009	Other	2.2.1
ERR108426	Russia	2009	MDR	2.2.1
ERR108428	Russia	2009	MDR	2.2.1
ERR108430	Russia	2009	Susceptible	2.2.1
ERR108433	Russia	2009	Susceptible	2.2.1
ERR108434	Russia	2009	Susceptible	2.2.1
ERR108435	Russia	2009	MDR	2.2.1
ERR108436	Russia	2009	MDR	2.2.1
ERR108439	Russia	2009	MDR	2.2.1
ERR108440	Russia	2009	Susceptible	2.2.1
ERR108441	Russia	2008	Susceptible	2.2.1
ERR108443	Russia	2009	MDR	2.2.1
ERR108447	Russia	2009	Other	2.2.1
ERR108450	Russia	2009	MDR	2.2.1
ERR108452	Russia	2009	Susceptible	2.2.1
ERR108453	Russia	2009	MDR	2.2.1
ERR108454	NA	NA	MDR	2.2.1
ERR108455	Russia	2009	MDR	2.2.1
ERR108458	Russia	2009	MDR	2.2.1
ERR108462	Russia	2008	MDR	2.2.1
ERR108467	Russia	2009	MDR	2.2.1
ERR108469	Russia	2009	MDR	2.2.1
ERR108470	Russia	2009	Susceptible	2.2.1
ERR108473	Russia	2009	MDR	2.2.1
ERR108475	Russia	2009	MDR	2.2.1
ERR108477	NA	NA	MDR	2.2.1
ERR108479	Russia	2009	Susceptible	2.2.1
ERR108484	Russia	2009	MDR	2.2.1
ERR108486	Russia	2009	Susceptible	2.2.1
ERR108488	Russia	2009	MDR	2.2.1
ERR108489	Russia	2009	MDR	2.2.1
ERR108490	Russia	2009	Susceptible	2.2.1
ERR108491	Russia	2009	MDR	2.2.1
ERR108492	Russia	2009	Other	2.2.1
ERR108493	Russia	2009	MDR	2.2.1
ERR108494	Russia	2009	Other	2.2.1
ERR108495	Russia	2009	MDR	2.2.1
ERR108496	Russia	2009	Susceptible	2.2.1
ERR108498	Russia	2008	MDR	2.2.1
ERR108499	Russia	2008	MDR	2.2.1
ERR108500	Russia	2008	MDR	2.2.1
ERR108501	Russia	2008	Susceptible	2.2.1
ERR108502	Russia	2008	MDR	2.2.1
ERR108503	Russia	2009	MDR	2.2.1
ERR108505	Russia	2009	Other	2.2.1
ERR108506	Russia	2009	Susceptible	2.2.1
ERR108507	Russia	2009	MDR	2.2.1
ERR108510	Russia	2009	MDR	2.2.1
ERR108511	Russia	2009	MDR	2.2.1
ERR108513	Russia	2009	Susceptible	2.2.1
ERR108514	Russia	2008	MDR	2.2.1
ERR108515	Russia	2008	MDR	2.2.1
ERR114436	NA	NA	Other	2.2.1

ERR114457	NA	NA	MDR	2.2.1
ERR114474	NA	NA	Susceptible	2.2.1
ERR114485	NA	NA	Susceptible	2.2.1
ERR114488	NA	NA	Other	2.2.1
ERR114514	NA	NA	MDR	2.2.1
ERR1161615	Estonia	2005	MDR	2.2.1
ERR1161616	Estonia	2005	Other	2.2.1
ERR1161617	Estonia	2005	MDR	2.2.1
ERR1161618	Estonia	2005	MDR	2.2.1
ERR1161619	Estonia	2008	XDR	2.2.1
ERR1161620	Estonia	2008	MDR	2.2.1
ERR1161621	Estonia	2009	MDR	2.2.1
ERR1161622	Estonia	2009	MDR	2.2.1
ERR1161623	Estonia	2009	MDR	2.2.1
ERR1161624	Estonia	2009	MDR	2.2.1
ERR117449	Russia	2008	Susceptible	2.2.1
ERR117453	Russia	2008	MDR	2.2.1
ERR117454	Russia	2009	MDR	2.2.1
ERR117455	Russia	2009	Susceptible	2.2.1
ERR117456	Russia	2009	MDR	2.2.1
ERR117457	Russia	2009	Susceptible	2.2.1
ERR117458	Russia	2009	Susceptible	2.2.1
ERR117459	Russia	2009	MDR	2.2.1
ERR117460	Russia	2009	MDR	2.2.1
ERR117462	Russia	2009	XDR	2.2.1
ERR117463	Russia	2009	MDR	2.2.1
ERR117464	Russia	2009	MDR	2.2.1
ERR117465	Russia	2009	Susceptible	2.2.1
ERR117467	Russia	2009	Susceptible	2.2.1
ERR117468	Russia	2009	MDR	2.2.1
ERR117469	Russia	2008	Other	2.2.1
ERR117470	Russia	2009	MDR	2.2.1
ERR117634	NA	NA	Susceptible	2.2.1
ERR117657	NA	NA	Susceptible	2.2
ERR117663	NA	NA	MDR	2.2.1
ERR117687	NA	NA	XDR	2.2.2
ERR117695	NA	NA	MDR	2.2.1
ERR117707	NA	NA	MDR	2.2.1
ERR117716	NA	NA	Other	2.2.1
ERR117722	NA	NA	Susceptible	2.2.1
ERR117735	NA	NA	Other	2.2.1
ERR117737	NA	NA	MDR	2.2.1
ERR1194779	Norway	2014	Other	2.2.1
ERR1194780	Norway	2014	Other	2.2.1
ERR1194781	Norway	2014	Other	2.2.1
ERR1194782	Norway	2014	Other	2.2.1
ERR1194783	Norway	2014	Other	2.2.1
ERR1194784	Norway	2014	Other	2.2.1
ERR1194785	Norway	2014	Other	2.2.1
ERR1194786	Norway	2014	Other	2.2.1
ERR1194787	Norway	2014	Other	2.2.1
ERR1194788	Norway	2014	Other	2.2.1
ERR1194789	Norway	2014	Other	2.2.1
ERR1194790	Norway	2014	Other	2.2.1
ERR1194791	Norway	2014	Other	2.2.1
ERR1194792	Norway	2014	Other	2.2.1
ERR1194793	Norway	2014	Other	2.2.1
ERR1194794	Norway	2014	Other	2.2.1
ERR1194795	Norway	2014	Other	2.2.1
ERR1194796	Norway	2014	Other	2.2.1
ERR1194797	Norway	2014	Other	2.2.1

ERR1194798	Norway	2014	Other	2.2.1
ERR1194799	Norway	2014	Other	2.2.1
ERR1194800	Norway	2014	Other	2.2.1
ERR1194801	Norway	2014	Other	2.2.1
ERR1194802	Norway	2014	Other	2.2.1
ERR1194803	Norway	2014	Other	2.2.1
ERR1194804	Norway	2014	Other	2.2.1
ERR1194805	Norway	2014	Other	2.2.1
ERR1213827	NA	NA	Susceptible	2.2.2
ERR1213828	NA	NA	Susceptible	2.2.1
ERR1213830	NA	NA	Susceptible	2.2.2
ERR1213831	NA	NA	Susceptible	2.2.1
ERR1213832	NA	NA	Other	2.2.1
ERR1213834	NA	NA	Other	2.2.1
ERR1213835	NA	NA	Other	2.2.1
ERR1213836	NA	NA	MDR	2.2.1
ERR1213840	NA	NA	Other	2.2.1
ERR1213846	NA	NA	Other	2.2.1
ERR1213847	NA	NA	Other	2.2.2
ERR1213849	NA	NA	MDR	2.2.2
ERR1213851	NA	NA	MDR	2.2.2
ERR1213853	NA	NA	MDR	2.2.2
ERR1213855	NA	NA	Other	2.2.1
ERR1213856	NA	NA	Other	2.2.1
ERR1213857	NA	NA	MDR	2.2.2
ERR1213858	NA	NA	MDR	2.2.1
ERR1213859	NA	NA	MDR	2.2.2
ERR1213860	NA	NA	Other	2.2.2
ERR1213864	NA	NA	Other	2.2.2
ERR1213865	NA	NA	MDR	2.2.2
ERR1213874	NA	NA	MDR	2.2.1
ERR1213880	NA	NA	Other	2.2.1
ERR1213886	NA	NA	MDR	2.2.1
ERR1213887	NA	NA	MDR	2.2.1.1
ERR1213893	NA	NA	Other	2.2.1
ERR1213897	NA	NA	Susceptible	2.2.2
ERR1213901	NA	NA	MDR	2.2.1
ERR1213929	NA	NA	Susceptible	2.2.1
ERR1227524	NA	NA	MDR	2.2.1
ERR123925	NA	NA	Susceptible	2.2.1
ERR123946	NA	NA	Susceptible	2.2.1
ERR126606	Malawi	2007	Susceptible	2.2.1
ERR133798	Russia	2008	Other	2.2.1
ERR133799	Russia	2008	Other	2.2.1
ERR133802	Russia	2008	Susceptible	2.2.1
ERR133805	Russia	2008	MDR	2.2.1
ERR133806	Russia	2008	MDR	2.2.1
ERR133810	Russia	2008	Susceptible	2.2.1
ERR133814	Russia	2008	Other	2.2.1
ERR133815	Russia	2008	Other	2.2.1
ERR133820	Russia	2008	Susceptible	2.2.1
ERR133826	Russia	2008	MDR	2.2.1
ERR133827	Russia	2008	MDR	2.2.1
ERR133831	Russia	2008	MDR	2.2.1
ERR133837	Russia	2008	Other	2.2.1
ERR133839	Russia	2008	MDR	2.2.1
ERR133840	Russia	2008	Susceptible	2.2.1
ERR133842	Russia	2008	Other	2.2.1
ERR133843	NA	NA	Susceptible	2.2.1
ERR133844	Russia	2008	MDR	2.2.1
ERR133845	Russia	2008	MDR	2.2.1

ERR133847	Russia	2008	Susceptible	2.2.1
ERR133848	Russia	2008	XDR	2.2.1
ERR133852	Russia	2008	MDR	2.2.1
ERR133853	Russia	2008	MDR	2.2.1
ERR133854	Russia	2008	MDR	2.2.1
ERR133855	Russia	2008	MDR	2.2.1
ERR133856	Russia	2008	MDR	2.2.1
ERR133859	Russia	2008	Susceptible	2.2.1
ERR133860	Russia	2008	MDR	2.2.1
ERR133861	Russia	2008	MDR	2.2.1
ERR133862	Russia	2008	Other	2.2.1
ERR133863	Russia	2008	Other	2.2.1
ERR133865	Russia	2008	Other	2.2.1
ERR133866	Russia	2008	MDR	2.2.1
ERR133867	Russia	2008	MDR	2.2.1
ERR133868	Russia	2008	MDR	2.2.1
ERR133869	Russia	2008	Other	2
ERR133871	Russia	2008	Susceptible	2.2.1
ERR133873	Russia	2008	MDR	2.2.1
ERR133875	Russia	2008	Susceptible	2.2.1
ERR133879	Russia	2008	MDR	2.2.1
ERR133880	Russia	2008	MDR	2.2.1
ERR133882	Russia	2008	Other	2.2.1
ERR133884	Russia	2008	MDR	2.2.1
ERR133885	Russia	2008	MDR	2.2.1
ERR133886	Russia	2008	MDR	2.2.1
ERR133888	Russia	2008	Other	2.2.1
ERR133889	Russia	2008	MDR	2.2.1
ERR133890	Russia	2008	MDR	2.2.1
ERR133891	Russia	2008	Susceptible	2.2.1
ERR133892	Russia	2008	MDR	2.2.1
ERR133894	Russia	2009	Other	2.2.1
ERR133895	Russia	2009	MDR	2.2.1
ERR133896	Russia	2008	MDR	2.2.1
ERR133898	Russia	2009	Susceptible	2.2.1
ERR133899	Russia	2009	MDR	2.2.1
ERR133900	Russia	2009	Other	2.2.1
ERR133901	Russia	2009	MDR	2.2.1
ERR133902	Russia	2009	MDR	2.2.1
ERR133904	Russia	2009	Susceptible	2.2.1
ERR133906	Russia	2009	MDR	2.2.1
ERR133907	Russia	2009	MDR	2.2.1
ERR133908	Russia	2009	MDR	2.2.1
ERR133910	Russia	2009	MDR	2.2.1
ERR133913	Russia	2009	Other	2.2.1
ERR133914	Russia	2009	Other	2.2.1
ERR133915	Russia	2009	MDR	2.2.1
ERR133916	NA	NA	Other	2.2.1
ERR133918	Russia	2009	MDR	2.2.1
ERR133919	Russia	2009	MDR	2.2.1
ERR133924	Russia	2009	Other	2.2.1
ERR133925	Russia	2009	Susceptible	2.2.1
ERR133926	Russia	2009	Susceptible	2.2.1
ERR133927	Russia	2009	MDR	2.2.1
ERR133929	Russia	2009	Susceptible	2.2.1
ERR133930	Russia	2009	MDR	2.2.1
ERR133931	Russia	2009	MDR	2.2.1
ERR133932	Russia	2009	Susceptible	2.2.1
ERR133933	Russia	2009	MDR	2.2.1
ERR133935	Russia	2009	MDR	2.2.1
ERR133936	Russia	2009	MDR	2.2.1

ERR133941	Russia	2009	Susceptible	2.2.1
ERR133944	Russia	2009	MDR	2.2.1
ERR133945	Russia	2009	Other	2.2.1
ERR133946	Russia	2009	Susceptible	2.2.1
ERR133947	Russia	2009	Other	2.2.1
ERR133948	Russia	2009	Susceptible	2.2.1
ERR133950	Russia	2008	Susceptible	2.2.1
ERR133951	Russia	2009	MDR	2.2.1
ERR133953	Russia	2009	MDR	2.2.1
ERR133954	Russia	2009	MDR	2.2.1
ERR133955	Russia	2009	Other	2.2.1
ERR133957	Russia	2009	MDR	2.2.1
ERR133958	Russia	2009	Susceptible	2.2.1
ERR133959	Russia	2009	MDR	2.2.1
ERR133960	Russia	2009	Susceptible	2.2.1
ERR133963	Russia	2009	Other	2.2.1
ERR133966	Russia	2009	MDR	2.2.1
ERR133968	Russia	2009	Other	2.2.1
ERR133970	Russia	2009	MDR	2.2.1
ERR133973	Russia	2009	Other	2.2.1
ERR133975	Russia	2009	Other	2.2.1
ERR133976	Russia	2009	Other	2.2.1
ERR133979	Russia	2009	Susceptible	2.2.1
ERR133982	Russia	2009	MDR	2.2.1
ERR133983	Russia	2009	Susceptible	2.2.1
ERR133984	Russia	2009	MDR	2.2.1
ERR133987	Russia	2009	Susceptible	2.2.1
ERR133988	Russia	2009	Other	2.2.1
ERR1352298	China	2009	Susceptible	2.2.1
ERR1352299	China	2009	Susceptible	2.2.1
ERR1352300	China	2009	Susceptible	2.2.1
ERR1352301	China	2009	Susceptible	2.2.1
ERR1352302	China	2009	Susceptible	2.2.1
ERR1352303	China	2009	Susceptible	2.2.1
ERR1352304	China	2009	Susceptible	2.2.1
ERR1352305	China	2009	Susceptible	2.2.1
ERR1352306	China	2009	Susceptible	2.2.1
ERR1352307	China	2009	Susceptible	2.2.1
ERR1352308	China	2009	Susceptible	2.2.1
ERR1352309	China	2009	Susceptible	2.2.1
ERR1352310	China	2009	Susceptible	2.2.1
ERR1352311	China	2009	Susceptible	2.2.1
ERR1352312	China	2009	Susceptible	2.2.1
ERR1352313	China	2009	Susceptible	2.2.1
ERR1352314	China	2009	Susceptible	2.2.1
ERR1352315	China	2009	Susceptible	2.2.1
ERR1352316	China	2009	Susceptible	2.2.1
ERR1352317	China	2009	Susceptible	2.2.1
ERR1352318	China	2009	Susceptible	2.2.1
ERR1352324	China	2009	Susceptible	2.2.1
ERR1352325	China	2009	Susceptible	2.2.1
ERR1352326	China	2009	Susceptible	2.2.1
ERR1352327	China	2009	Susceptible	2.2.1
ERR1352328	China	2009	Susceptible	2.2.1
ERR1352329	China	2009	MDR	2.2.1
ERR1352330	China	2009	MDR	2.2.1
ERR1352331	China	2009	MDR	2.2.1
ERR1352332	China	2009	MDR	2.2.1
ERR1352333	China	2009	MDR	2.2.1
ERR1352334	China	2009	MDR	2.2.1
ERR1352335	China	2009	MDR	2.2.1

ERR1352336	China	2009	MDR	2.2.1
ERR1352337	China	2009	MDR	2.2.1
ERR1352338	China	2009	MDR	2.2.1
ERR1352339	China	2009	MDR	2.2.1
ERR1352340	China	2009	MDR	2.2.1
ERR1352341	China	2009	MDR	2.2.1
ERR1352342	China	2009	MDR	2.2.1
ERR1352343	China	2009	MDR	2.2.1
ERR1352344	China	2009	XDR	2.2.1
ERR1352345	China	2009	Other	2.2.2
ERR1352346	China	2009	Other	2.2.2
ERR1352347	China	2009	Other	2.2.2
ERR1352348	China	2009	Susceptible	2.2.2
ERR1352349	China	2009	Other	2.2.2
ERR1352350	China	2009	XDR	2.2.1
ERR1352351	China	2009	XDR	2.2.1
ERR1352352	China	2009	XDR	2.2.1
ERR1352353	China	2009	XDR	2.2.1
ERR1352354	China	2009	XDR	2.2.1
ERR1352355	China	2009	XDR	2.2.1
ERR1352356	China	2009	XDR	2.2.1
ERR1352357	China	2009	XDR	2.2.1
ERR1352358	China	2009	XDR	2.2.1
ERR1367615	NA	NA	MDR	2.2.1
ERR1367637	NA	NA	Susceptible	2.2.1
ERR1367641	NA	NA	Other	2.2.1
ERR1367646	NA	NA	Other	2.2.1
ERR1367648	NA	NA	Other	2.2.1
ERR1367667	NA	NA	MDR	2.2.1
ERR137192	Russia	2009	MDR	2.2.1
ERR137195	Russia	2009	Susceptible	2.2.1
ERR137198	Russia	2009	Other	2.2.1
ERR137203	Russia	2009	Susceptible	2.2.1
ERR137204	Russia	2009	MDR	2.2.1
ERR137205	Russia	2009	Susceptible	2.2.1
ERR137206	Russia	2009	Other	2.2.1
ERR137207	Russia	2009	Susceptible	2.2.1
ERR137208	Russia	2010	MDR	2.2.1
ERR137210	Russia	2009	MDR	2.2.1
ERR137211	Russia	2009	MDR	2.2.1
ERR137212	Russia	2009	Other	2.2.1
ERR137215	Russia	2009	Susceptible	2.2.1
ERR137216	Russia	2009	Other	2.2.1
ERR137217	Russia	2009	MDR	2.2.1
ERR137218	Russia	2009	MDR	2.2.1
ERR137219	Russia	2009	MDR	2.2.1
ERR137220	Russia	2009	MDR	2.2.1
ERR137221	Russia	2009	Other	2.2.1
ERR137222	Russia	2009	MDR	2.2.1
ERR137223	Russia	2009	MDR	2.2.1
ERR137225	Russia	2009	MDR	2.2.1
ERR137226	NA	NA	MDR	2.2.1
ERR137227	Russia	2009	Other	2.2.1
ERR137229	Russia	2009	MDR	2.2.1
ERR137230	Russia	2009	MDR	2.2.1
ERR137231	Russia	2009	XDR	2.2.1
ERR137232	Russia	2009	Susceptible	2.2.1
ERR137234	Russia	2009	MDR	2.2.1
ERR137235	Russia	2009	Susceptible	2.2.1
ERR137236	Russia	2009	MDR	2.2.1
ERR137237	Russia	2009	MDR	2.2.1

ERR137240	Russia	2009	Other	2.2.1
ERR137241	Russia	2009	Susceptible	2.2.1
ERR137242	Russia	2009	Other	2.2.1
ERR137243	Russia	2009	MDR	2.2.1
ERR137244	Russia	2009	MDR	2.2.1
ERR137246	Russia	2009	MDR	2.2.1
ERR137247	Russia	2009	MDR	2.2.1
ERR137248	Russia	2009	XDR	2.2.1
ERR137249	Russia	2009	Susceptible	2.2.1
ERR137252	Russia	2009	Susceptible	2.2.1
ERR137253	Russia	2009	Susceptible	2.2.1
ERR137254	Russia	2009	MDR	2.2.1
ERR137255	Russia	2009	MDR	2.2.1
ERR137257	Russia	2009	MDR	2.2.1
ERR137259	Russia	2009	MDR	2.2.1
ERR137260	Russia	2009	MDR	2.2.1
ERR137262	Russia	2009	MDR	2.2.1
ERR137263	Russia	2009	Susceptible	2.2.1
ERR137264	Russia	2009	MDR	2.2.1
ERR137265	Russia	2009	MDR	2.2.1
ERR137267	Russia	2009	Other	2.2.1
ERR137268	Russia	2009	Susceptible	2.2.1
ERR137269	Russia	2009	MDR	2.2.1
ERR137272	Russia	2009	MDR	2.2.1
ERR137273	Russia	2009	MDR	2.2.1
ERR137275	Russia	2009	XDR	2.2.1
ERR137277	Russia	2009	MDR	2.2.1
ERR137278	Russia	2009	Other	2.2.1
ERR137279	Russia	2009	Susceptible	2.2.1
ERR137281	Russia	2009	MDR	2.2.1
ERR137282	Russia	2009	MDR	2.2.1
ERR137283	Russia	2009	MDR	2.2.1
ERR137285	Russia	2009	Susceptible	2.2.1
ERR1394278	India	2013	MDR	2.2.1
ERR144542	Russia	2009	Other	2.2.1
ERR144543	Russia	2009	MDR	2.2.1
ERR144545	Russia	2009	Other	2.2.1
ERR144548	Russia	2009	MDR	2.2.1
ERR144549	Russia	2009	MDR	2.2.1
ERR144550	Russia	2009	Other	2.2.1
ERR144551	Russia	2009	MDR	2.2.1
ERR144552	Russia	2009	Susceptible	2.2.1
ERR144553	Russia	2009	MDR	2.2.1
ERR144554	Russia	2009	MDR	2.2.1
ERR144556	Russia	2009	Susceptible	2.2
ERR144558	Russia	2009	MDR	2.2.1
ERR144560	Russia	2009	MDR	2.2.1
ERR144561	Russia	2009	MDR	2.2.1
ERR144562	Russia	2009	MDR	2.2.1
ERR144563	Russia	2009	MDR	2.2.1
ERR144564	Russia	2009	Other	2.2.1
ERR144566	Russia	2009	MDR	2.2.1
ERR144567	Russia	2009	XDR	2.2.1
ERR144570	Russia	2009	MDR	2.2.1
ERR144571	Russia	2009	MDR	2.2.1
ERR144572	Russia	2009	MDR	2.2.1
ERR144573	Russia	2009	MDR	2.2.1
ERR144574	Russia	2009	MDR	2.2.1
ERR144575	Russia	2009	MDR	2.2.1
ERR144577	Russia	2009	Other	2.2.1
ERR144579	Russia	2009	MDR	2.2.1

ERR144580	Russia	2009	Other	2.2.1
ERR144581	Russia	2009	MDR	2.2.1
ERR144582	Russia	2009	Susceptible	2.2.1
ERR144584	Russia	2009	MDR	2.2.1
ERR144588	Russia	2009	Other	2.2.1
ERR144589	NA	NA	Susceptible	2.2.1
ERR144590	Russia	2009	MDR	2.2.1
ERR144591	NA	NA	Other	2.2.1
ERR144593	Russia	2009	MDR	2.2.1
ERR144594	Russia	2009	Other	2.2.1
ERR144595	Russia	2009	MDR	2.2.1
ERR144596	Russia	2009	MDR	2.2.1
ERR144597	Russia	2009	MDR	2.2.1
ERR144599	Russia	2009	Other	2.2.1
ERR144600	Russia	2009	MDR	2.2.1
ERR144601	NA	NA	MDR	2.2.1
ERR144606	Russia	2009	Susceptible	2.2.1
ERR144607	Russia	2009	MDR	2.2.1
ERR144608	Russia	2009	MDR	2.2.1
ERR144609	Russia	2009	Susceptible	2.2.1
ERR144610	Russia	2009	Susceptible	2.2.1
ERR144611	Russia	2009	MDR	2.2.1
ERR144614	Russia	2009	Susceptible	2.2.1
ERR144615	Russia	2009	MDR	2.2.1
ERR144616	Russia	2009	Susceptible	2.2.1
ERR144617	NA	NA	MDR	2.2.1
ERR144619	Russia	2009	MDR	2.2.1
ERR144620	Russia	2009	MDR	2.2.1
ERR144622	Russia	2009	MDR	2.2.1
ERR144623	Russia	2009	MDR	2.2.1
ERR144625	Russia	2009	Susceptible	2.2.1
ERR144626	Russia	2009	Susceptible	2.2.1
ERR144628	Russia	2009	MDR	2.2.1
ERR144630	Russia	2009	Susceptible	2.2.1
ERR144631	Russia	2009	MDR	2.2.1
ERR144632	Russia	2009	MDR	2.2.1
ERR144633	Russia	2009	MDR	2.2.1
ERR144634	Russia	2009	MDR	2.2.1
ERR144635	Russia	2009	MDR	2.2.1
ERR144636	Russia	2009	XDR	2.2.1
ERR1452609	South Africa	1996	MDR	2.2.2
ERR1452610	South Africa	1996	Susceptible	2.2.1
ERR1452611	South Africa	1996	Susceptible	2.2.1
ERR1452612	South Africa	1996	Susceptible	2.2.1
ERR1452613	South Africa	1996	Susceptible	2.2.1
ERR1452614	South Africa	1996	Susceptible	2.2.1
ERR1452615	South Africa	1996	Susceptible	2.2.1.1
ERR1452616	South Africa	1996	Susceptible	2.2.1.1
ERR1452617	South Africa	1996	Susceptible	2.2.1.1
ERR152759	NA	NA	MDR	2.2.1
ERR152760	United Kingdom	2010	Other	2.2.2
ERR152761	United Kingdom	2010	Other	2.2.2
ERR1544431	Kazakhstan	2014	MDR	2.2.1
ERR1544432	Kazakhstan	2014	MDR	2.2.1
ERR1544433	Kazakhstan	2014	MDR	2.2.1
ERR1544434	Kazakhstan	2014	MDR	2.2.1
ERR1544435	Kazakhstan	2014	MDR	2.2.1
ERR1544436	Kazakhstan	2014	MDR	2.2.1
ERR1544437	Kazakhstan	2014	MDR	2.2.1
ERR1544438	Kazakhstan	2014	MDR	2.2.1
ERR1544439	Kazakhstan	2014	MDR	2.2.1

ERR1544440	Kazakhstan	2014	MDR	2.2.1
ERR1544441	Kazakhstan	2014	MDR	2.2.1
ERR1559736	Kazakhstan	2014	MDR	2.2.1
ERR1559737	Kazakhstan	2014	MDR	2.2.1
ERR1559738	Kazakhstan	2014	MDR	2.2.1
ERR1559739	Kazakhstan	2014	MDR	2.2.1
ERR1559740	Kazakhstan	2014	MDR	2.2.1
ERR1559741	Kazakhstan	2014	MDR	2.2.1
ERR1559742	Kazakhstan	2014	MDR	2.2.1
ERR1559743	Kazakhstan	2014	MDR	2.2.1
ERR1577246	Norway	2015	Susceptible	2.2.1
ERR1577247	Norway	2015	Susceptible	2.2.1
ERR1577249	Norway	2015	Other	2.2.1
ERR158569	Russia	2009	Other	2.2.1
ERR158570	Russia	2009	MDR	2.2.1
ERR158572	Russia	2009	Other	2.2.1
ERR158574	Russia	2009	Susceptible	2.2.1
ERR158575	Russia	2009	Susceptible	2.2.1
ERR158576	Russia	2009	MDR	2.2.1
ERR158577	Russia	2009	Susceptible	2.2.1
ERR158578	Russia	2009	MDR	2.2.1
ERR158580	Russia	2009	MDR	2.2.1
ERR158581	Russia	2009	MDR	2.2.1
ERR158585	Russia	2009	MDR	2.2.1
ERR158586	Russia	2009	MDR	2.2.1
ERR158587	Russia	2009	MDR	2.2.1
ERR158588	Russia	2009	MDR	2.2.1
ERR158589	Russia	2009	Other	2.2.1
ERR158590	Russia	2009	Other	2.2.1
ERR158591	Russia	2009	Other	2.2.1
ERR158592	Russia	2009	MDR	2.2.1
ERR158593	Russia	2009	Susceptible	2.2.1
ERR158594	Russia	2009	Other	2.2.1
ERR158596	Russia	2009	MDR	2.2.1
ERR158597	Russia	2009	MDR	2.2.1
ERR158598	Russia	2009	MDR	2.2.1
ERR158602	United Kingdom	2011	MDR	2.2.1
ERR158605	United Kingdom	2011	Susceptible	2.2.1
ERR158606	United Kingdom	2011	MDR	2.2.1
ERR158607	United Kingdom	2011	MDR	2.2.1
ERR158608	United Kingdom	2011	MDR	2.2.1
ERR158609	United Kingdom	2011	MDR	2.2.1.1
ERR158611	Russia	2008	MDR	2.2.1
ERR158613	Russia	2008	MDR	2.2.1
ERR158615	Russia	2008	Other	2.2.1
ERR158616	Russia	2008	MDR	2.2.1
ERR1633777	South Africa	2010	Susceptible	2.2.1.1
ERR1633791	South Africa	2010	Susceptible	2.2.2
ERR1633796	South Africa	2010	MDR	2.2.2
ERR1633800	South Africa	2010	Susceptible	2.2.1.1
ERR1633809	South Africa	2010	Susceptible	2.2.1.1
ERR1633816	South Africa	2010	Susceptible	2.2.1
ERR1633819	South Africa	2010	MDR	2.2.1.1
ERR1633822	South Africa	2010	Susceptible	2.2.1
ERR1633823	South Africa	2010	Susceptible	2.2.1.1
ERR1633828	South Africa	2010	Susceptible	2.2.1.1
ERR1633829	South Africa	2010	Susceptible	2.2.1.1
ERR1633831	South Africa	2010	Susceptible	2.2.1
ERR1633835	South Africa	2010	Susceptible	2.2.1.1
ERR1633843	South Africa	2010	Susceptible	2.2.1
ERR1633844	South Africa	2010	Susceptible	2.2.1.1

ERR1633868	South Africa	2010	Susceptible	2.2.1
ERR1633872	South Africa	2010	Susceptible	2.2.1
ERR1633881	South Africa	2010	MDR	2.2.1
ERR1633882	South Africa	2010	Susceptible	2.2.1.1
ERR1633885	South Africa	2010	Susceptible	2.2.1.1
ERR1633895	South Africa	2010	Susceptible	2.2.1
ERR1633896	South Africa	2010	Susceptible	2.2.1
ERR1633915	South Africa	2010	Susceptible	2.2.1.1
ERR1633922	South Africa	2010	Susceptible	2.2.1.1
ERR1633925	South Africa	2010	Susceptible	2.2.1
ERR1633928	South Africa	2010	Susceptible	2.2.1
ERR1633933	South Africa	2010	Susceptible	2.2.1.1
ERR1633938	South Africa	2010	Susceptible	2.2.1
ERR1633939	South Africa	2010	Other	2.2.1.1
ERR1633944	South Africa	2010	Susceptible	2.2.1
ERR1633946	South Africa	2010	Susceptible	2.2.1.1
ERR1633948	South Africa	2010	Susceptible	2.2.1.1
ERR1633951	South Africa	2010	Susceptible	2.2.1
ERR1633955	South Africa	2010	Susceptible	2.2.1
ERR1633956	South Africa	2010	MDR	2.2.1
ERR1633962	South Africa	2010	Susceptible	2.2.1.1
ERR1633964	South Africa	2010	MDR	2.2.1
ERR163959	Malawi	2004	Susceptible	2.2.1
ERR163990	Malawi	2004	Susceptible	2.2.1
ERR1665402	Spain	NA	MDR	2.2.1
ERR1665404	Spain	NA	MDR	2.2.1
ERR171135	South Africa	2008/2014	Susceptible	2.2.1
ERR171138	South Africa	2008/2014	Susceptible	2.2.1
ERR171144	South Africa	2008/2014	Susceptible	2.2.1
ERR171145	South Africa	2008/2014	Susceptible	2.2.1
ERR171148	South Africa	2008/2014	Susceptible	2.2.1
ERR171149	South Africa	2008/2014	Susceptible	2.2.1
ERR171154	Malaysia	2008/2014	Susceptible	2.2.1
ERR171155	Malaysia	2008/2014	Susceptible	2.2.1
ERR171160	Thailand	2008/2014	Susceptible	2.2.1
ERR171161	Thailand	2008/2014	Susceptible	2.2.1
ERR171162	Thailand	2008/2014	Other	2.2.1
ERR171163	Thailand	2008/2014	Other	2.2.1
ERR1726500	China	2015	Susceptible	2.2.1
ERR1726501	China	2015	Susceptible	2.2.1
ERR1726502	China	2015	Susceptible	2.2.1
ERR1726503	China	2015	Susceptible	2.2.1
ERR1750885	Zimbabwe	2008/2012	Susceptible	2.2.1
ERR1750886	Zimbabwe	2008/2012	Susceptible	2.2.1
ERR1750890	Zimbabwe	2008/2012	Susceptible	2.2.1
ERR1750895	Zimbabwe	2008/2012	Susceptible	2.2.1
ERR1750896	Zimbabwe	2008/2012	Susceptible	2.2.1
ERR1750902	South Africa	2008/2012	Susceptible	2.2.1
ERR1750911	South Africa	2008/2012	Susceptible	2.2.1
ERR1750912	South Africa	2008/2012	Susceptible	2.2.1
ERR1750915	South Africa	2008/2012	Susceptible	2.2.1.1
ERR1750916	South Africa	2008/2012	Susceptible	2.2.1.1
ERR1750917	South Africa	2008/2012	Susceptible	2.2.1
ERR1750918	South Africa	2008/2012	Susceptible	2.2.1
ERR1750933	South Africa	2008/2012	Susceptible	2.2.1
ERR1750939	South Africa	2008/2012	Susceptible	2.2.1.1
ERR1750940	South Africa	2008/2012	Susceptible	2.2.1.1
ERR176606	Malawi	1998	Susceptible	2.2.1
ERR176611	Malawi	2000	Susceptible	2.2
ERR176641	Malawi	1999	Susceptible	2.2.1
ERR176718	Malawi	2000	Susceptible	2.2.1

ERR176759	Malawi	1998	Susceptible	2.2.1
ERR176794	Malawi	1999	Susceptible	2.2.1
ERR176799	Malawi	1999	Susceptible	2.2.1
ERR181316	NA	NA	Susceptible	2.1
ERR181440	NA	NA	Susceptible	2.2.1
ERR181696	Malawi	2006	Susceptible	2.2.1
ERR181722	Malawi	2005	Susceptible	2.2.1
ERR181769	Malawi	2001	Susceptible	2.2.1
ERR181821	Malawi	2008	Susceptible	2.2.1
ERR181828	Malawi	2008	Susceptible	2.2.1
ERR181849	Malawi	2008	Susceptible	2.2.1
ERR181866	Malawi	1998	Susceptible	2.2.1
ERR181870	Malawi	1998	Susceptible	2.2.1
ERR181891	Malawi	2001	Susceptible	2.2.1
ERR181898	Malawi	2001	Susceptible	2.2.1
ERR181905	Malawi	2001	Susceptible	2.2.1
ERR181916	Malawi	2001	Susceptible	2.2.1
ERR181918	Malawi	2001	Susceptible	2.2.1
ERR181941	Malawi	2004	Susceptible	2.2.1
ERR181960	Malawi	2004	Susceptible	2.2.1
ERR181965	Malawi	2005	Susceptible	2.2.1
ERR1873389	South Africa	2009	MDR	2.2.2
ERR1873390	South Africa	2009	MDR	2.2.1
ERR1873394	South Africa	2009	MDR	2.2.1
ERR1873395	South Africa	2008	MDR	2.2.2
ERR1873397	South Africa	2010	MDR	2.2.2
ERR1873399	South Africa	2010	XDR	2.2.1
ERR1873402	South Africa	2011	MDR	2.2.2
ERR1873404	South Africa	2011	MDR	2.2.1
ERR1873407	South Africa	2011	MDR	2.2.2
ERR1873409	South Africa	2009	MDR	2.2.2
ERR1873410	South Africa	2010	XDR	2.2.1
ERR1873412	South Africa	2010	XDR	2.2.1
ERR1873413	South Africa	2010	MDR	2.2.1
ERR1873414	South Africa	2010	MDR	2.2.1
ERR1873415	South Africa	2011	MDR	2.2.1
ERR1873416	South Africa	2010	MDR	2.2.1
ERR1873417	South Africa	2011	MDR	2.2.2
ERR1873421	South Africa	2009	MDR	2.2.2
ERR1873422	South Africa	2010	XDR	2.2.2
ERR1873424	South Africa	2010	Other	2.2.2
ERR1873427	South Africa	2010	MDR	2.2.2
ERR1873428	South Africa	2010	Other	2.2.2
ERR1873429	South Africa	2010	XDR	2.2.2
ERR1873430	South Africa	2010	MDR	2.2.2
ERR1873431	South Africa	2009	MDR	2.2.1
ERR1873432	South Africa	2010	MDR	2.2.1
ERR1873434	South Africa	2010	MDR	2.2.2
ERR1873436	South Africa	2010	MDR	2.2.2
ERR1873438	South Africa	2011	XDR	2.2.1
ERR1873439	South Africa	2011	MDR	2.2.2
ERR1873440	South Africa	2011	XDR	2.2.2
ERR1873442	South Africa	2010	MDR	2.2.1
ERR1873443	South Africa	2010	MDR	2.2.2
ERR1873445	South Africa	2010	MDR	2.2.1
ERR1873447	South Africa	2010	XDR	2.2.1
ERR1873450	South Africa	2010	MDR	2.2.1
ERR1873451	South Africa	2010	XDR	2.2.1
ERR1873452	South Africa	2010	MDR	2.2.2
ERR1873453	South Africa	2010	MDR	2.2.2
ERR1873454	South Africa	2010	MDR	2.2.2

ERR1873455	South Africa	2010	MDR	2.2.1
ERR1873456	South Africa	2010	XDR	2.2.2
ERR1873457	South Africa	2010	XDR	2.2.1
ERR1873458	South Africa	2010	XDR	2.2.1
ERR1873460	South Africa	2010	XDR	2.2.2
ERR1873461	South Africa	2010	MDR	2.2.2
ERR1873463	South Africa	2011	MDR	2.2.2
ERR1873464	South Africa	2011	XDR	2.2.1
ERR1873465	South Africa	2011	MDR	2.2.1
ERR1873466	South Africa	2011	MDR	2.2.2
ERR1873467	South Africa	2011	MDR	2.2.2
ERR1873468	South Africa	2011	MDR	2.2.2
ERR1873469	South Africa	2011	MDR	2.2.1
ERR1873470	South Africa	2011	XDR	2.2.1
ERR1873471	South Africa	2011	MDR	2.2.1
ERR1873472	South Africa	2011	MDR	2.2.2
ERR1873473	South Africa	2011	MDR	2.2.2
ERR1873474	South Africa	2011	XDR	2.2.1
ERR1873476	South Africa	2011	MDR	2.2.2
ERR1873477	South Africa	2011	MDR	2.2.1
ERR1873478	South Africa	2011	MDR	2.2.1
ERR1873479	South Africa	2011	MDR	2.2.1
ERR1873480	South Africa	2011	Susceptible	2.2
ERR1873481	South Africa	2011	MDR	2.2.1
ERR1873482	South Africa	2012	MDR	2.2.1
ERR1873484	South Africa	2011	XDR	2.2.1
ERR1873485	South Africa	2012	MDR	2.2.2
ERR1873486	South Africa	2011	MDR	2.2.2
ERR1873487	South Africa	2011	MDR	2.2.1
ERR1873488	South Africa	2012	XDR	2.2.1
ERR1873489	South Africa	2011	MDR	2.2.2
ERR1873490	South Africa	2012	XDR	2.2.2
ERR1873491	South Africa	2012	MDR	2.2.1
ERR1873493	South Africa	2012	MDR	2.2.2
ERR1873494	South Africa	2012	MDR	2.2.2
ERR1873495	South Africa	2012	MDR	2.2.2
ERR1873496	South Africa	2012	MDR	2.2.2
ERR1873497	South Africa	2012	MDR	2.2.2
ERR1873498	South Africa	2012	MDR	2.2.2
ERR1873499	South Africa	2012	MDR	2.2.1
ERR1873500	South Africa	2012	MDR	2.2.2
ERR1873501	South Africa	2012	MDR	2.2.1
ERR1873502	South Africa	2012	MDR	2.2.2
ERR1873503	South Africa	2012	MDR	2.2.2
ERR1873504	South Africa	2012	MDR	2.2.2
ERR1873505	South Africa	2012	MDR	2.2.2
ERR1873507	South Africa	2012	MDR	2.2.2
ERR1873508	South Africa	2012	Other	2.2.1
ERR1873509	South Africa	2012	XDR	2.2.1
ERR1873510	South Africa	2012	MDR	2.2.2
ERR1873514	South Africa	2012	MDR	2.2.2
ERR1873515	South Africa	2012	MDR	2.2.2
ERR1873516	South Africa	2012	MDR	2.2.2
ERR1873519	South Africa	2012	XDR	2.2.2
ERR1873520	South Africa	2012	MDR	2.2.2
ERR1873521	South Africa	2008	MDR	2.2.2
ERR1873522	South Africa	2008	MDR	2.2.1.1
ERR1873523	South Africa	2008	XDR	2.2.2
ERR1873524	South Africa	2008	MDR	2.2.2
ERR1873525	South Africa	2008	MDR	2.2.2
ERR1873526	South Africa	2008	MDR	2.2.2

ERR1873527	South Africa	2008	MDR	2.2.1
ERR1873528	South Africa	2008	MDR	2.2.2
ERR1873529	South Africa	2008	XDR	2.2.2
ERR1873530	South Africa	2009	MDR	2.2.2
ERR1873532	South Africa	2009	MDR	2.2.1
ERR1873533	South Africa	2009	MDR	2.2.2
ERR1873535	South Africa	2009	XDR	2.2.1
ERR1873536	South Africa	2009	MDR	2.2.2
ERR1873537	South Africa	2009	MDR	2.2.1
ERR1873538	South Africa	2009	MDR	2.2.2
ERR1873539	South Africa	2009	MDR	2.2.2
ERR1873541	South Africa	2009	MDR	2.2.2
ERR1873542	South Africa	2009	MDR	2.2.2
ERR1873543	South Africa	2009	MDR	2.2.1
ERR1873544	South Africa	2009	XDR	2.2.2
ERR1873545	South Africa	2009	MDR	2.2.1
ERR1873547	South Africa	2009	MDR	2.2.2
ERR1873548	South Africa	2009	MDR	2.2.2
ERR1873551	South Africa	2009	XDR	2.2.2
ERR1873552	South Africa	2009	MDR	2.2.2
ERR1873553	South Africa	2010	XDR	2.2.2
ERR1873555	South Africa	2010	MDR	2.2.1
ERR1873556	South Africa	2010	XDR	2.2.1
ERR1873557	South Africa	2010	MDR	2.2.1
ERR1873558	South Africa	2010	MDR	2.2.1
ERR1873559	South Africa	2010	MDR	2.2.1
ERR1873560	South Africa	2010	MDR	2.2.1
ERR1873561	South Africa	2010	MDR	2.2.2
ERR1873562	South Africa	2010	XDR	2.2.1
ERR1873563	South Africa	2010	MDR	2.2.1
ERR1873564	South Africa	2010	XDR	2.2.2
ERR1873565	South Africa	2010	MDR	2.2.1
ERR190363	Malawi	2002	Susceptible	2.2.1
ERR190368	Malawi	2001	Susceptible	2.2.1
ERR190369	Malawi	2002	Susceptible	2.2.1
ERR190378	Malawi	1999	Susceptible	2.2.1
ERR190386	Malawi	1998	Susceptible	2.2.1
ERR190401	Malawi	2002	Susceptible	2.2.1
ERR192451	NA	NA	MDR	2.2.1
ERR1952141	Ireland	2004	XDR	2.2.1
ERR198708	NA	NA	MDR	2.2.1
ERR1988847	South Africa	2010	XDR	2.2.2
ERR202405	NA	NA	Susceptible	2.2.1
ERR202407	NA	NA	Other	2.2.1
ERR202409	NA	NA	Susceptible	2.2.1
ERR212013	Malawi	1999	Susceptible	2.2.1
ERR212014	Malawi	1999	Susceptible	2.2.1
ERR212037	Malawi	1998	Susceptible	2.2.1
ERR212081	Malawi	1999	Susceptible	2.2.1
ERR212098	Malawi	2000	Susceptible	2.2.1
ERR212104	Malawi	1998	Susceptible	2.2.1
ERR212120	Malawi	2001	Susceptible	2.2.1
ERR212126	Malawi	2001	Susceptible	2.2.1
ERR212145	Malawi	2009	Susceptible	2.2.1
ERR216958	Malawi	2010	Susceptible	2.2.1
ERR216962	Malawi	2010	Susceptible	2.2.1
ERR218154	United States of America	NA	Susceptible	2.1
ERR221524	Malawi	2000	Susceptible	2.2.1
ERR221536	Malawi	2001	Susceptible	2.2
ERR221538	Malawi	2001	Susceptible	2.2.1
ERR221542	Malawi	2002	Susceptible	2.2.1

ERR221544	Malawi	2002	Susceptible	2.2.1
ERR221545	Malawi	2002	Susceptible	2.2.1
ERR221553	Malawi	2002	Susceptible	2.2.1
ERR221558	Malawi	2003	Susceptible	2.2.1
ERR221572	Malawi	2003	Susceptible	2.2.1
ERR221573	Malawi	2003	Susceptible	2.2.1
ERR221574	Malawi	2003	Susceptible	2.2.1
ERR221584	Malawi	2008	Susceptible	2.2.1
ERR221603	Malawi	2001	Susceptible	2.2.1
ERR221604	Malawi	2002	Susceptible	2.2
ERR221605	Malawi	2003	Susceptible	2.2.1
ERR221628	NA	NA	Susceptible	2.2.1
ERR221629	NA	NA	Susceptible	2.2.1
ERR221630	NA	NA	Susceptible	2.2.1
ERR221631	NA	NA	Susceptible	2.2.1
ERR221632	NA	NA	Susceptible	2.2
ERR221633	NA	NA	Susceptible	2.2.1
ERR221634	NA	NA	Susceptible	2.2.1
ERR221652	NA	NA	Susceptible	2.2.1
ERR221655	NA	NA	Susceptible	2.2.1
ERR221658	NA	NA	Susceptible	2.2.1
ERR227975	Russia	2010	MDR	2.2.1
ERR227976	Russia	2010	MDR	2.2.1
ERR227977	Russia	2010	MDR	2.2.1
ERR227978	Russia	2010	MDR	2.2.1
ERR227979	Russia	2009	Susceptible	2.2.1
ERR227980	Russia	2010	Other	2.2.1
ERR227981	Russia	2010	MDR	2.2.1
ERR227982	Russia	2009	MDR	2.2.1
ERR227983	Russia	2010	MDR	2.2.1
ERR227984	Russia	2010	Susceptible	2.2.1
ERR227987	Russia	2009	MDR	2.2.1
ERR227995	Russia	2009	MDR	2.2.1
ERR227996	Russia	2010	XDR	2.2.1
ERR227999	Russia	2010	Susceptible	2.2.1
ERR228002	Russia	2010	Susceptible	2.2.1
ERR228003	Russia	2009	MDR	2.2.1
ERR228004	Russia	2010	Susceptible	2.2.1
ERR228006	Russia	2009	MDR	2.2.1
ERR228007	Russia	2010	Susceptible	2.2.1
ERR228010	Russia	2010	Susceptible	2.2.1
ERR228012	Russia	2010	MDR	2.2.1
ERR228013	Russia	2010	MDR	2.2.1
ERR228014	Russia	2009	Other	2.2.1
ERR228015	Russia	2010	Susceptible	2.2.1
ERR228017	Russia	2010	MDR	2.2.1
ERR228018	Russia	2010	Susceptible	2.2.1
ERR228019	Russia	2010	MDR	2.2.1
ERR228020	Russia	2010	MDR	2.2.1
ERR228022	Russia	2009	Susceptible	2.2.1
ERR228024	Russia	2010	MDR	2.2.1
ERR228027	Russia	2010	Susceptible	2.2.1
ERR228028	Russia	2010	MDR	2.2.1
ERR228029	Russia	2010	MDR	2.2.1
ERR228032	Russia	2010	MDR	2.2.1
ERR228037	Russia	2010	MDR	2.2.1
ERR228039	Russia	2010	MDR	2.2.1
ERR228040	Russia	2010	Other	2.2.1
ERR228042	Russia	2010	MDR	2.2.1
ERR228043	Russia	2010	MDR	2.2.1
ERR228046	Russia	2009	MDR	2.2.1

ERR228048	Russia	2010	Other	2.2.1
ERR228049	Russia	2010	MDR	2.2.1
ERR228050	Russia	2010	MDR	2.2.1
ERR228051	Russia	2010	MDR	2.2.1
ERR228052	Russia	2010	Susceptible	2.2.1
ERR228054	Russia	2009	MDR	2.2.1
ERR228056	Russia	2010	MDR	2.2.1
ERR228059	Russia	2010	MDR	2.2.1
ERR228060	Russia	2010	Susceptible	2.2.1
ERR228061	Russia	2010	MDR	2.2.1
ERR228062	Russia	2010	MDR	2.2.1
ERR228064	Russia	2010	MDR	2.2.1
ERR228066	Russia	2009	Other	2.2.1
ERR228068	Russia	2010	Susceptible	2.2.1
ERR228104	NA	NA	Other	2.2.1
ERR228127	NA	NA	Other	2.2.1
ERR228179	NA	NA	Susceptible	2.2.1
ERR228184	NA	NA	Other	2.2.1
ERR228186	NA	NA	Other	2.2.1
ERR228192	NA	NA	Susceptible	2.2.1
ERR228202	NA	NA	Other	2.2.1
ERR228226	NA	NA	Susceptible	2.2.1
ERR228237	NA	NA	Susceptible	2.2.1
ERR228269	NA	NA	Susceptible	2.2.1.1
ERR228273	NA	NA	Susceptible	2.2.1
ERR229915	Russia	2010	MDR	2.2.1
ERR229916	Russia	2010	MDR	2.2.1
ERR229918	Russia	2010	Other	2.2.1
ERR229920	Russia	2010	Susceptible	2.2.1
ERR229921	Russia	2010	MDR	2.2.1
ERR229922	Russia	2010	MDR	2.2.1
ERR229923	Russia	2010	MDR	2.2.1
ERR229924	Russia	2010	MDR	2.2.1
ERR229926	Russia	2010	MDR	2.2.1
ERR229929	Russia	2010	XDR	2.2.1
ERR229930	NA	NA	Susceptible	2
ERR229931	Russia	2010	MDR	2.2.1
ERR229933	Russia	2010	MDR	2.2.1
ERR229935	Russia	2010	Other	2.2.1
ERR229936	Russia	2010	MDR	2.2.1
ERR229939	Russia	2010	Susceptible	2.2.1
ERR229940	Russia	2010	MDR	2.2.1
ERR229945	Russia	2010	Susceptible	2.2.1
ERR229946	Russia	2010	MDR	2.2.1
ERR229947	Russia	2010	MDR	2.2.1
ERR229949	Russia	2010	MDR	2.2.1
ERR229955	Russia	2010	MDR	2.2.1
ERR229957	Russia	2010	Other	2.2.1
ERR229960	Russia	2010	XDR	2.2.1
ERR229961	Russia	2010	MDR	2.2.1
ERR229962	Russia	2010	MDR	2.2.1
ERR229964	Russia	2010	MDR	2.2.1
ERR229965	Russia	2010	MDR	2.2.1
ERR229966	Russia	2010	MDR	2.2.1
ERR229968	Russia	2010	MDR	2.2.1
ERR229969	Russia	2010	MDR	2.2.1
ERR229970	Russia	2010	Susceptible	2.2.1
ERR229971	Russia	2010	MDR	2.2.1
ERR229975	Russia	2010	Susceptible	2.2.1
ERR229976	Russia	2010	MDR	2.2.1
ERR229977	Russia	2010	Susceptible	2.2.1

ERR229978	Russia	2010	XDR	2.2.1
ERR229980	Russia	2010	MDR	2.2.1
ERR229982	Russia	2010	XDR	2.2.1
ERR229984	Russia	2010	XDR	2.2.1
ERR229992	Russia	2010	Susceptible	2.2.1
ERR229993	Russia	2010	MDR	2.2.1
ERR229994	Russia	2010	MDR	2.2.1
ERR229995	Russia	2010	MDR	2.2.1
ERR229997	Russia	2010	MDR	2.2.1
ERR229998	Russia	2010	MDR	2.2.1
ERR229999	Russia	2010	Other	2.2.1
ERR230000	Russia	2010	MDR	2.2.1
ERR230004	Russia	2010	Susceptible	2.2.1
ERR230005	Russia	2010	MDR	2.2.1
ERR230006	Russia	2010	Other	2.2.1
ERR230007	Russia	2010	Other	2.2.1
ERR230008	Russia	2010	MDR	2.2.1
ERR230009	NA	NA	MDR	2.2.1
ERR230010	Russia	2010	Other	2.2.1
ERR230045	NA	NA	Other	2.2.1
ERR230055	NA	NA	Susceptible	2.2.1
ERR230061	NA	NA	Susceptible	2.2.1
ERR230079	NA	NA	Susceptible	2.2.1
ERR230084	NA	NA	Susceptible	2.2.1
ERR230100	NA	NA	Susceptible	2.2.1.1
ERR230109	NA	NA	Susceptible	2.2.1
ERR234098	NA	NA	Susceptible	2.2.1
ERR234100	NA	NA	Susceptible	2.2.1
ERR234102	NA	NA	Susceptible	2.2.1
ERR234103	NA	NA	Susceptible	2.2.1
ERR234108	NA	NA	Susceptible	2.2.1
ERR234110	NA	NA	Susceptible	2.2.1
ERR234112	NA	NA	Other	2.2.1
ERR234114	NA	NA	Susceptible	2.2.1
ERR234115	NA	NA	MDR	2.2.1
ERR234116	NA	NA	Susceptible	2.2.1
ERR234117	NA	NA	Susceptible	2.2.1
ERR234118	NA	NA	Susceptible	2.2.1
ERR234119	NA	NA	Susceptible	2.2.1
ERR234120	NA	NA	Susceptible	2.2.1
ERR234121	NA	NA	Susceptible	2.2.1.1
ERR234122	NA	NA	Susceptible	2.2.1
ERR234123	NA	NA	Susceptible	2.2.1
ERR234124	NA	NA	MDR	2.2.1
ERR234125	NA	NA	Susceptible	2.2.1
ERR234126	NA	NA	Susceptible	2.2.1
ERR234127	NA	NA	Susceptible	2.2.1
ERR234128	NA	NA	Susceptible	2.2.1
ERR234129	NA	NA	Susceptible	2.2.1
ERR234130	NA	NA	Susceptible	2.2.1.1
ERR234131	NA	NA	Susceptible	2.2.1
ERR234132	NA	NA	Susceptible	2.2.1
ERR234133	NA	NA	Susceptible	2.2.1
ERR234134	NA	NA	Susceptible	2.2.1
ERR234135	NA	NA	Susceptible	2.2.1
ERR234136	NA	NA	Susceptible	2.2.1
ERR234137	NA	NA	Other	2.2.1
ERR234138	NA	NA	Susceptible	2.2.1
ERR234139	NA	NA	Susceptible	2.2.1
ERR234140	NA	NA	Susceptible	2.2.1.1
ERR234141	NA	NA	Susceptible	2.2.1

ERR234190	NA	NA	Susceptible	2.2.1.1
ERR234193	NA	NA	Susceptible	2.2.1
ERR234208	NA	NA	Susceptible	2.2.1
ERR234209	NA	NA	Susceptible	2.2.1
ERR234210	NA	NA	Susceptible	2.2.1
ERR234211	NA	NA	Susceptible	2.2.2
ERR234213	NA	NA	Susceptible	2.2.1.2
ERR234216	NA	NA	Susceptible	2.1
ERR234242	NA	NA	Susceptible	2.2.1.1
ERR234245	NA	NA	Susceptible	2.2.1
ERR234246	NA	NA	Susceptible	2.2.1.1
ERR234247	NA	NA	Susceptible	2.2.1
ERR234248	NA	NA	Susceptible	2.1
ERR234249	NA	NA	Susceptible	2.2.1.1
ERR234250	NA	NA	Susceptible	2.2.1
ERR234252	NA	NA	Susceptible	2.1
ERR234253	NA	NA	Susceptible	2.2.1
ERR234256	NA	NA	Susceptible	2.2.1.1
ERR234263	NA	NA	Other	2.2.1.2
ERR234269	NA	NA	Susceptible	2.2.1
ERR234270	NA	NA	Susceptible	2.2.1
ERR234271	NA	NA	Susceptible	2.2.1
ERR234556	Russia	2008	MDR	2.2.1
ERR234557	Russia	2008	Susceptible	2.2.1
ERR234559	Russia	2008	Susceptible	2.2.1
ERR234560	Russia	2008	MDR	2.2.1
ERR234562	Russia	2009	MDR	2.2.1
ERR234563	Russia	2009	MDR	2.2.1
ERR234565	Russia	2008	MDR	2.2.1
ERR234568	Russia	2008	Other	2.2.1
ERR234569	Russia	2008	Other	2.2.1
ERR234570	Russia	2009	MDR	2.2.1
ERR234573	Russia	2008	MDR	2.2.1
ERR234574	Russia	2008	MDR	2.2.1
ERR234575	Russia	2008	Other	2.2.1
ERR234576	Russia	2008	MDR	2.2.1
ERR234579	Russia	2009	Susceptible	2.2.1
ERR234581	Russia	2008	Susceptible	2.2.1
ERR234582	Russia	2008	Susceptible	2.2.1
ERR234584	Russia	2008	MDR	2.2.1
ERR234585	Russia	2008	Other	2.2.1
ERR234589	Russia	2008	MDR	2.2.1
ERR234590	Russia	2008	XDR	2.2.1
ERR234591	Russia	2008	Susceptible	2.2.1
ERR234593	Russia	2008	MDR	2.2.1
ERR234595	Russia	2009	MDR	2.2.1
ERR234596	Russia	2008	XDR	2.2.1
ERR234597	Russia	2008	MDR	2.2.1
ERR234598	Russia	2008	XDR	2.2.1
ERR234600	Russia	2008	MDR	2.2.1
ERR234601	NA	NA	MDR	2.2.1
ERR234602	Russia	2009	MDR	2.2.1
ERR234603	Russia	2009	Other	2.2.1
ERR234606	Russia	2008	MDR	2.2.1
ERR234607	Russia	2008	MDR	2.2.1
ERR234608	Russia	2008	MDR	2.2.1
ERR234609	Russia	2009	MDR	2.2.1
ERR234610	Russia	2009	MDR	2.2.1
ERR234612	Russia	2008	MDR	2.2.1
ERR234615	Russia	2008	MDR	2.2.1
ERR234617	Russia	2009	Susceptible	2.2.1

ERR234618	Russia	2009	MDR	2.2.1
ERR234619	Russia	2009	Susceptible	2.2.1
ERR234620	Russia	2008	Other	2.2.1
ERR234621	NA	NA	Other	2.2.1
ERR234622	Russia	2008	MDR	2.2.1
ERR234623	Russia	2008	MDR	2.2.1
ERR234624	Russia	2008	Other	2.2.1
ERR234628	Russia	2008	MDR	2.2.1
ERR234629	NA	NA	Other	2.2.1
ERR234630	Russia	2008	MDR	2.2.1
ERR234632	Russia	2008	MDR	2.2.1
ERR234633	Russia	2009	MDR	2.2.1
ERR234637	Russia	2008	MDR	2.2.1
ERR234638	Russia	2008	Susceptible	2.2.1
ERR234639	Russia	2008	MDR	2.2.1
ERR234640	Russia	2008	Susceptible	2.2.1
ERR234641	Russia	2009	MDR	2.2.1
ERR234642	Russia	2009	Susceptible	2.2.1
ERR234643	Russia	2009	MDR	2.2
ERR234644	Russia	2008	MDR	2.2.1
ERR234645	Russia	2008	Other	2.2.1
ERR234646	Russia	2008	MDR	2.2.1
ERR234647	Russia	2008	Susceptible	2.2.1
ERR234649	Russia	2009	MDR	2.2.1
ERR234650	Russia	2009	MDR	2.2.1
ERR234651	Russia	2009	Other	2.2.1
ERR234652	Russia	2010	Susceptible	2.2.1
ERR234653	Russia	2010	MDR	2.2.1
ERR234655	Russia	2010	Susceptible	2.2.1
ERR234656	Russia	2010	MDR	2.2.1
ERR234657	Russia	2010	XDR	2.2.1
ERR234658	Russia	2010	MDR	2.2.1
ERR234660	Russia	2010	MDR	2.2.1
ERR234661	Russia	2010	MDR	2.2.1
ERR234662	Russia	2010	Other	2.2.1
ERR234663	Russia	2010	Susceptible	2.2.1
ERR234664	Russia	2010	Susceptible	2.2.1
ERR234666	Russia	2010	Other	2.2.1
ERR234668	Russia	2010	MDR	2.2.1
ERR234670	NA	NA	MDR	2.2.1
ERR234671	Russia	2010	MDR	2.2.1
ERR234672	Russia	2010	MDR	2.2.1
ERR234683	Russia	2010	Other	2.2.1
ERR234688	Russia	2010	MDR	2.2.1
ERR234690	Russia	2010	Susceptible	2.2.1
ERR234692	Russia	2010	MDR	2.2.1
ERR234698	Russia	2010	MDR	2.2.1
ERR245655	Malawi	2004	Susceptible	2.2.1
ERR245660	Malawi	2004	Susceptible	2.2.1
ERR245663	Malawi	2005	Susceptible	2.2.1
ERR245680	Malawi	2005	Susceptible	2.2.1
ERR245692	Malawi	2006	Susceptible	2.2.1
ERR245696	Malawi	2006	Susceptible	2.2.1
ERR245710	Malawi	2007	Susceptible	2.2.1
ERR245716	Malawi	2006	Susceptible	2.2.1
ERR245723	Malawi	2007	Susceptible	2.2.1
ERR245728	Malawi	2007	Susceptible	2.2.1
ERR245735	Malawi	2007	Susceptible	2.2.1
ERR245783	Malawi	2000	Susceptible	2.2.1
ERR245831	Malawi	2003	Susceptible	2.2.1
ERR245834	Malawi	2003	Susceptible	2.2.1

ERR245837	Malawi	2003	Susceptible	2.2.1
ERR245846	Malawi	2004	Susceptible	2.2.1
ERR257921	Netherlands	1995	Other	2.2.1
ERR257922	Netherlands	2003	Other	2.2.1
ERR257923	Netherlands	1995	Other	2.2.1
ERR257924	Netherlands	1995	Other	2.2.1
ERR257925	Netherlands	1994	Other	2.2.1
ERR257926	Netherlands	1995	Other	2.2.1
ERR257927	Netherlands	1995	Other	2.2.1
ERR257928	Netherlands	1995	Other	2.2.1
ERR257929	Netherlands	1997	Other	2.2.1
ERR257930	Netherlands	2004	Other	2.2.1
ERR266521	NA	NA	MDR	2.2.1
ERR266539	NA	NA	Susceptible	2.2.1
ERR266553	NA	NA	Other	2.2.1
ERR266584	NA	NA	Susceptible	2.2.1.1
ERR266595	NA	NA	Susceptible	2.2.1
ERR270627	NA	NA	Susceptible	2.2.1
ERR270630	NA	NA	MDR	2.2.1
ERR270637	NA	NA	Susceptible	2.2.1
ERR270679	NA	NA	MDR	2.2.1
ERR270693	NA	NA	XDR	2.2.2
ERR270697	NA	NA	Other	2.1
ERR270718	NA	NA	MDR	2.2.2
ERR270720	NA	NA	XDR	2.2.1
ERR270769	NA	NA	Other	2.2.1
ERR270773	NA	NA	Other	2.2.1
ERR270774	NA	NA	Other	2.2.1
ERR270780	NA	NA	Other	2.2.1
ERR270789	NA	NA	Other	2.2.1
ERR278522	NA	NA	MDR	2.2.1
ERR278524	NA	NA	Other	2.2.1
ERR278569	NA	NA	MDR	2.2.1
ERR278593	NA	NA	Susceptible	2.2.1
ERR279468	NA	NA	MDR	2.2.2
ERR279471	NA	NA	Susceptible	2.2.1.1
ERR279472	NA	NA	Susceptible	2.2.1
ERR279478	NA	NA	Susceptible	2.2.1.1
ERR279491	NA	NA	Susceptible	2.2.2
ERR279496	NA	NA	Susceptible	2.2.1
ERR279499	NA	NA	Susceptible	2.2.1.1
ERR279507	NA	NA	Susceptible	2.2.1.1
ERR279514	NA	NA	Susceptible	2.2.1
ERR279516	NA	NA	MDR	2.2.1.1
ERR279519	NA	NA	Susceptible	2.2.1
ERR279520	NA	NA	Susceptible	2.2.1.1
ERR279525	NA	NA	Susceptible	2.2.1.1
ERR279527	NA	NA	Susceptible	2.2.1
ERR279531	NA	NA	Susceptible	2.2.1.1
ERR279538	NA	NA	Susceptible	2.2.1
ERR279539	NA	NA	Susceptible	2.2.1.1
ERR279557	NA	NA	Susceptible	2.2.1.1
ERR279561	NA	NA	Susceptible	2.2.1
ERR279562	NA	NA	MDR	2.2.2
ERR279563	NA	NA	MDR	2.2.1
ERR279569	NA	NA	Susceptible	2.2.1.1
ERR279571	NA	NA	MDR	2.2.1
ERR279573	NA	NA	Susceptible	2.2.1
ERR279581	NA	NA	Susceptible	2.2
ERR279582	NA	NA	Susceptible	2.2.1.1
ERR279585	NA	NA	Susceptible	2.2.1.1

ERR279594	NA	NA	Susceptible	2.2.1
ERR279595	NA	NA	Susceptible	2.2.1
ERR279600	NA	NA	Susceptible	2.2.1.1
ERR279613	NA	NA	Susceptible	2.2.1.1
ERR279620	NA	NA	Susceptible	2.2.1
ERR279622	NA	NA	Susceptible	2.2.1
ERR279625	NA	NA	Susceptible	2.2.1
ERR279629	NA	NA	Susceptible	2.2.2
ERR279630	NA	NA	Susceptible	2.2.1.1
ERR279634	NA	NA	Susceptible	2.2.1
ERR279635	NA	NA	Other	2.2.1.1
ERR279640	NA	NA	Susceptible	2.2.1
ERR279641	NA	NA	Susceptible	2.2.1.1
ERR279643	NA	NA	Susceptible	2.2.1.1
ERR279646	NA	NA	Susceptible	2.2.1
ERR279650	NA	NA	Susceptible	2.2.1
ERR279651	NA	NA	MDR	2.2.1
ERR279655	NA	NA	Susceptible	2.2.1.1
ERR279656	NA	NA	Susceptible	2.2.1
ERR279657	NA	NA	Susceptible	2.2.1.1
ERR294198	NA	NA	Other	2.2.1
ERR294206	NA	NA	Susceptible	2.2.1.1
ERR294263	NA	NA	XDR	2.2.1
ERR294267	NA	NA	MDR	2.2.1
ERR323119	NA	NA	Susceptible	2.2.1
ERR330672	NA	NA	Other	2.2.1
ERR330688	NA	NA	Susceptible	2.2.1
ERR330711	NA	NA	Other	2.2.1
ERR330740	NA	NA	Susceptible	2.2.1
ERR351895	NA	NA	Other	2.2.1.1
ERR351920	NA	NA	Susceptible	2.2.1
ERR351928	NA	NA	Susceptible	2.2.1
ERR356389	NA	NA	MDR	2.2.1
ERR356390	NA	NA	MDR	2.2.1
ERR386827	NA	NA	Other	2.2.1.1
ERR386852	NA	NA	Susceptible	2.2.1
ERR386860	NA	NA	Susceptible	2.2.1
ERR386881	NA	NA	MDR	2.2.1
ERR386883	NA	NA	Other	2.2.1
ERR386928	NA	NA	MDR	2.2.1
ERR386952	NA	NA	Susceptible	2.2.1
ERR386982	NA	NA	Other	2.2.1
ERR386985	NA	NA	Susceptible	2.2.1
ERR386992	NA	NA	XDR	2.2.1
ERR386993	NA	NA	Susceptible	2.2.1
ERR387011	NA	NA	MDR	2.2.1
ERR387032	NA	NA	Other	2.2.1
ERR392507	NA	NA	Other	2.2.1.2
ERR392508	NA	NA	Susceptible	2.2.1.1
ERR392509	NA	NA	Susceptible	2.2.1.1
ERR400317	NA	NA	Susceptible	2.2.1
ERR400324	NA	NA	Susceptible	2.2.1
ERR400325	NA	NA	Susceptible	2.2.1.1
ERR400331	NA	NA	Other	2.2.1
ERR400332	NA	NA	Susceptible	2.2.1
ERR400341	NA	NA	Other	2.2.1
ERR400349	NA	NA	Susceptible	2.2.1
ERR400352	NA	NA	Susceptible	2.2.1
ERR400378	NA	NA	Other	2.2.1
ERR400382	NA	NA	MDR	2.2.1
ERR400387	NA	NA	Susceptible	2.2.1

ERR400400	NA	NA	Susceptible	2.2.1
ERR400407	NA	NA	Susceptible	2.2.1.1
ERR400414	NA	NA	Other	2.2.1
ERR400424	NA	NA	Susceptible	2.2.2
ERR400436	NA	NA	Susceptible	2.2.1
ERR400444	NA	NA	Susceptible	2.2.1
ERR400445	NA	NA	Susceptible	2.2.1
ERR400447	NA	NA	Susceptible	2.2.1
ERR400452	NA	NA	Susceptible	2.2.1
ERR400458	NA	NA	Susceptible	2.2.1
ERR400464	NA	NA	Susceptible	2.2.1
ERR400481	NA	NA	Other	2.2.1
ERR400493	NA	NA	Other	2.2.1
ERR400494	NA	NA	Susceptible	2.2.1
ERR400503	NA	NA	Susceptible	2.2.1
ERR400509	NA	NA	Susceptible	2.2.1
ERR400512	NA	NA	Susceptible	2.2.1
ERR400517	NA	NA	XDR	2.2.1
ERR400531	NA	NA	Susceptible	2.2.1
ERR400533	NA	NA	Susceptible	2.2.1.1
ERR400546	NA	NA	Susceptible	2.2.1.1
ERR400555	NA	NA	Susceptible	2.2.1.1
ERR403214	NA	NA	MDR	2.2.1
ERR403215	NA	NA	Susceptible	2.2.1
ERR403216	NA	NA	Susceptible	2.2.1
ERR403217	NA	NA	Susceptible	2.2.1
ERR403218	NA	NA	MDR	2.2.1
ERR403219	NA	NA	MDR	2.2.1
ERR403221	NA	NA	MDR	2.2.1
ERR403222	NA	NA	MDR	2.2.1
ERR403223	NA	NA	Susceptible	2.2.1
ERR403224	NA	NA	MDR	2.2.1
ERR403225	NA	NA	Other	2.2.1
ERR403226	NA	NA	Susceptible	2.2.1
ERR403227	NA	NA	Other	2.2.1
ERR403228	NA	NA	MDR	2.2.1
ERR403229	NA	NA	MDR	2.2.1
ERR403230	NA	NA	MDR	2.2.1
ERR403232	NA	NA	Other	2.2.1
ERR403234	NA	NA	Other	2.2.1
ERR403235	NA	NA	Other	2.2.1
ERR403236	NA	NA	MDR	2.2.1
ERR403237	NA	NA	Susceptible	2.2.1
ERR403238	NA	NA	Susceptible	2.2.1
ERR403240	NA	NA	MDR	2.2.1
ERR403241	NA	NA	Other	2.2.1
ERR403242	NA	NA	MDR	2.2.1
ERR403244	NA	NA	MDR	2.2.1
ERR403245	NA	NA	MDR	2.2.1
ERR403246	NA	NA	Susceptible	2.2.2
ERR403247	NA	NA	MDR	2.2.1
ERR403248	NA	NA	Other	2.2.1
ERR403250	NA	NA	MDR	2.2.1
ERR403252	NA	NA	Other	2.2.1
ERR403253	NA	NA	Susceptible	2.2.1
ERR403254	NA	NA	Other	2.2.1
ERR403259	NA	NA	MDR	2.2.1
ERR403262	NA	NA	Susceptible	2.2.1
ERR403263	NA	NA	Other	2.2.1
ERR403264	NA	NA	Other	2.2.1
ERR403266	NA	NA	MDR	2.2.1

ERR403268	NA	NA	MDR	2.2.1
ERR403269	NA	NA	Susceptible	2.2.1
ERR403270	NA	NA	Susceptible	2.2.1
ERR403271	NA	NA	MDR	2.2.1
ERR403272	NA	NA	Susceptible	2.2.1
ERR403273	NA	NA	MDR	2.2.1
ERR403275	NA	NA	Susceptible	2.2.1
ERR403276	NA	NA	MDR	2.2.1
ERR403278	NA	NA	Susceptible	2.2.1
ERR403279	NA	NA	MDR	2.2.1
ERR403281	NA	NA	Susceptible	2.2.1
ERR403284	NA	NA	MDR	2.2.1
ERR403286	NA	NA	MDR	2.2.1
ERR403287	NA	NA	Susceptible	2.2.1
ERR403288	NA	NA	Susceptible	2.2.1
ERR403289	NA	NA	MDR	2.2.1
ERR403290	NA	NA	MDR	2.2.1
ERR403293	NA	NA	Susceptible	2.2.1
ERR403295	NA	NA	MDR	2.2.1
ERR403296	NA	NA	MDR	2.2.1
ERR403297	NA	NA	MDR	2.2.1
ERR403300	NA	NA	Other	2.2.1
ERR403301	NA	NA	MDR	2.2.1
ERR403302	NA	NA	MDR	2.2.1
ERR403303	NA	NA	MDR	2.2.1
ERR403304	NA	NA	Susceptible	2.2.1
ERR403305	NA	NA	MDR	2.2.1
ERR403306	NA	NA	XDR	2.2.1
ERR403307	NA	NA	MDR	2.2.1
ERR403309	NA	NA	Susceptible	2.2.1
ERR403310	NA	NA	Susceptible	2.2.1
ERR403313	NA	NA	MDR	2.2.1
ERR403316	NA	NA	MDR	2.2.1
ERR403318	NA	NA	MDR	2.2.1
ERR403324	NA	NA	MDR	2.2.1
ERR403327	NA	NA	MDR	2.2.1
ERR403345	NA	NA	Susceptible	2.2.1
ERR403359	NA	NA	Other	2.2.1
ERR403390	NA	NA	Susceptible	2.2.1.1
ERR403401	NA	NA	Susceptible	2.2.1
ERR405212	NA	NA	Other	2.2.1
ERR405215	NA	NA	Susceptible	2.2.1
ERR405217	NA	NA	Susceptible	2.2.1
ERR405221	NA	NA	MDR	2.2.1.1
ERR440680	NA	NA	MDR	2.2.1.1
ERR473283	NA	NA	Susceptible	2.2.1
ERR473304	NA	NA	Susceptible	2.2.1
ERR473326	NA	NA	Susceptible	2.2.1
ERR494999	NA	NA	MDR	2.2.2
ERR495002	NA	NA	Susceptible	2.2.1.1
ERR495009	NA	NA	Susceptible	2.2.1.1
ERR495022	NA	NA	Susceptible	2.2.2
ERR495027	NA	NA	Susceptible	2.2.1
ERR495030	NA	NA	Susceptible	2.2.1.1
ERR495038	NA	NA	Susceptible	2.2.1.1
ERR495045	NA	NA	Susceptible	2.2.1
ERR495047	NA	NA	MDR	2.2.1.1
ERR495050	NA	NA	Susceptible	2.2.1
ERR495051	NA	NA	Susceptible	2.2.1.1
ERR495056	NA	NA	Susceptible	2.2.1.1
ERR495058	NA	NA	Susceptible	2.2.1

ERR495062	NA	NA	Susceptible	2.2.1.1
ERR495069	NA	NA	Susceptible	2.2.1
ERR495070	NA	NA	Susceptible	2.2.1.1
ERR495088	NA	NA	Susceptible	2.2.1.1
ERR495092	NA	NA	Susceptible	2.2.1
ERR495093	NA	NA	MDR	2.2.2
ERR495094	NA	NA	MDR	2.2.1
ERR495100	NA	NA	Susceptible	2.2.1.1
ERR495102	NA	NA	MDR	2.2.1
ERR495104	NA	NA	Susceptible	2.2.1
ERR495113	NA	NA	Susceptible	2.2.1.1
ERR495116	NA	NA	Susceptible	2.2.1.1
ERR495125	NA	NA	Susceptible	2.2.1
ERR495126	NA	NA	Susceptible	2.2.1
ERR495144	NA	NA	Susceptible	2.2.1.1
ERR495151	NA	NA	Susceptible	2.2.1.1
ERR495153	NA	NA	Susceptible	2.2.1
ERR495156	NA	NA	Susceptible	2.2.1
ERR495161	NA	NA	Susceptible	2.2.1.1
ERR495165	NA	NA	Susceptible	2.2.1
ERR495166	NA	NA	Other	2.2.1.1
ERR495171	NA	NA	Susceptible	2.2.1
ERR495174	NA	NA	Susceptible	2.2.1.1
ERR495177	NA	NA	Susceptible	2.2.1
ERR495181	NA	NA	Susceptible	2.2.1
ERR495182	NA	NA	MDR	2.2.1
ERR495186	NA	NA	Susceptible	2.2.1.1
ERR495187	NA	NA	Susceptible	2.2.1
ERR495188	NA	NA	Susceptible	2.2.1.1
ERR495199	NA	NA	Susceptible	2.2.1.1
ERR502884	NA	NA	MDR	2.2.1
ERR502885	NA	NA	XDR	2.2.2
ERR502886	NA	NA	MDR	2.2.1
ERR502887	NA	NA	Susceptible	2.2.1
ERR502888	NA	NA	Other	2.2.1
ERR502890	NA	NA	MDR	2.2.1
ERR502891	NA	NA	MDR	2.2.1
ERR502892	NA	NA	MDR	2.2.1
ERR502893	NA	NA	MDR	2.2.1
ERR502894	NA	NA	MDR	2.2.1
ERR502895	NA	NA	XDR	2.2.1
ERR502896	NA	NA	Other	2.2.1
ERR502897	NA	NA	Susceptible	2.2.1
ERR502898	NA	NA	Other	2.2.1
ERR502899	NA	NA	Other	2.2.1
ERR502900	NA	NA	Other	2.2.1.1
ERR502901	NA	NA	MDR	2.2.1
ERR502902	NA	NA	Susceptible	2.2.1
ERR502903	NA	NA	Susceptible	2.2.1
ERR502904	NA	NA	Susceptible	2.2.1
ERR502905	NA	NA	Susceptible	2.2.1
ERR502906	NA	NA	Other	2.2.1.1
ERR502907	NA	NA	Susceptible	2.2.1
ERR502908	NA	NA	Susceptible	2.2.1
ERR502911	NA	NA	Susceptible	2.2.1
ERR502912	NA	NA	Susceptible	2.2.1
ERR502913	NA	NA	Susceptible	2.2.1
ERR502914	NA	NA	Susceptible	2.2.1
ERR502915	NA	NA	Susceptible	2.2.1
ERR502916	NA	NA	Susceptible	2.2.1
ERR502917	NA	NA	Susceptible	2.2.1

ERR502918	NA	NA	Susceptible	2.2.1
ERR502919	NA	NA	Susceptible	2.2.1
ERR502920	NA	NA	Susceptible	2.2.1
ERR502921	NA	NA	MDR	2.2.1
ERR502922	NA	NA	MDR	2.2.1
ERR502923	NA	NA	MDR	2.2.1
ERR502924	NA	NA	Other	2.2.1
ERR502925	NA	NA	Other	2.2.1
ERR502926	NA	NA	Susceptible	2.2.2
ERR502927	NA	NA	MDR	2.2.1
ERR502928	NA	NA	XDR	2.2.1
ERR502929	NA	NA	Other	2.2.1
ERR502930	NA	NA	MDR	2.2.1
ERR502931	NA	NA	Susceptible	2.2.1
ERR502932	NA	NA	Other	2.2.1
ERR502933	NA	NA	MDR	2.2.1
ERR502934	NA	NA	MDR	2.2.1
ERR502935	NA	NA	Susceptible	2.2.1
ERR502936	NA	NA	Other	2.2.1
ERR502937	NA	NA	Other	2.2.1
ERR502941	NA	NA	MDR	2.2.1
ERR502944	NA	NA	MDR	2.2.1
ERR502945	NA	NA	Susceptible	2.2.1
ERR502946	NA	NA	MDR	2.2.2
ERR502947	NA	NA	MDR	2.2.1
ERR502948	NA	NA	Susceptible	2.2.1
ERR502949	NA	NA	MDR	2.2.1
ERR502952	NA	NA	MDR	2.2.1
ERR502953	NA	NA	Other	2.2.1
ERR502954	NA	NA	MDR	2.2.1
ERR502955	NA	NA	MDR	2.2.1
ERR502956	NA	NA	MDR	2.2.1
ERR502957	NA	NA	Other	2.2.1
ERR502958	NA	NA	Other	2.2.1
ERR538422	NA	NA	Susceptible	2.2.1.1
ERR538423	NA	NA	Susceptible	2.2.1
ERR538424	NA	NA	Susceptible	2.2.1.1
ERR538425	NA	NA	Susceptible	2.2.1
ERR538426	NA	NA	Susceptible	2.2.1
ERR538427	NA	NA	Susceptible	2.2.1.2
ERR538428	NA	NA	Susceptible	2.2.2
ERR538429	NA	NA	Susceptible	2.2.1.2
ERR538430	NA	NA	Susceptible	2.2.1.2
ERR538431	NA	NA	Susceptible	2.2.2
ERR538432	NA	NA	Susceptible	2.1
ERR550405	NA	NA	MDR	2.2.1
ERR562804	Singapore	NA	MDR	2.2.1
ERR562805	Singapore	NA	MDR	2.2.1
ERR562806	Singapore	NA	MDR	2.2.1
ERR562807	Singapore	NA	MDR	2.2.1
ERR562808	Singapore	NA	MDR	2.2.1
ERR562809	Singapore	NA	MDR	2.2.1
ERR718193	NA	NA	MDR	2.2.1
ERR718194	NA	NA	Susceptible	2.2.1
ERR718195	NA	NA	Susceptible	2.2.1.1
ERR718199	NA	NA	Susceptible	2.2.1
ERR718200	NA	NA	Susceptible	2.2.1
ERR718203	NA	NA	Susceptible	2.2.1
ERR718206	NA	NA	Susceptible	2.2.1
ERR718207	NA	NA	Susceptible	2.2.1
ERR718208	NA	NA	Susceptible	2.2.1

ERR718211	NA	NA	Other	2.2.1.1
ERR718212	NA	NA	Susceptible	2.2.1
ERR718213	NA	NA	Susceptible	2.2.1
ERR718214	NA	NA	Other	2.2.1
ERR718215	NA	NA	MDR	2.2.1
ERR718216	NA	NA	Susceptible	2.2.1
ERR718218	NA	NA	Other	2.2.1
ERR718221	NA	NA	Susceptible	2.2.2
ERR718225	NA	NA	Susceptible	2.2.1
ERR718226	NA	NA	Susceptible	2.2.1
ERR718228	NA	NA	Susceptible	2.2.1
ERR718230	NA	NA	Susceptible	2.2.1
ERR718232	NA	NA	Susceptible	2.2.1
ERR718238	NA	NA	Susceptible	2.2.1
ERR718241	NA	NA	MDR	2.2.1
ERR718244	NA	NA	Susceptible	2.2.1
ERR718245	NA	NA	Other	2.2.1
ERR718246	NA	NA	Susceptible	2.2.1
ERR718250	NA	NA	Susceptible	2.2.1
ERR718251	NA	NA	Susceptible	2.2.1
ERR718252	NA	NA	Susceptible	2.2.1
ERR718253	NA	NA	Susceptible	2.2.1
ERR718254	NA	NA	Susceptible	2.2.1.1
ERR718255	NA	NA	Susceptible	2.2.1
ERR718256	NA	NA	Other	2.2.1
ERR718258	NA	NA	Susceptible	2.2.1
ERR718261	NA	NA	Other	2.2.1
ERR718266	NA	NA	Other	2.2.1
ERR718267	NA	NA	Susceptible	2.2.1
ERR718273	NA	NA	MDR	2.2.1
ERR718274	NA	NA	Other	2.2.1
ERR718275	NA	NA	Susceptible	2.2.1
ERR718276	NA	NA	Susceptible	2.1
ERR718277	NA	NA	Other	2.2.1.1
ERR718279	NA	NA	Other	2.2.1
ERR718280	NA	NA	Other	2.2.1
ERR718282	NA	NA	Susceptible	2.2.1.1
ERR718290	NA	NA	Susceptible	2.2.1
ERR718291	NA	NA	Susceptible	2.2.1
ERR718294	NA	NA	Other	2.2.1
ERR718297	NA	NA	Susceptible	2.2.1
ERR718299	NA	NA	Susceptible	2.2.1
ERR718300	NA	NA	Susceptible	2.2.1
ERR718302	NA	NA	Susceptible	2.2.1
ERR718304	NA	NA	Susceptible	2.2.1
ERR718305	NA	NA	Susceptible	2.2.1
ERR718308	NA	NA	Susceptible	2.2.1
ERR718309	NA	NA	Susceptible	2.2.1
ERR718311	NA	NA	Susceptible	2.2.1
ERR718312	NA	NA	Susceptible	2.2.1
ERR718313	NA	NA	Other	2.2.1
ERR718314	NA	NA	Other	2.2.1
ERR718316	NA	NA	Other	2.2.1
ERR718317	NA	NA	Susceptible	2.2.1
ERR718319	NA	NA	Susceptible	2.2.1
ERR718320	NA	NA	Susceptible	2.2.1
ERR718321	NA	NA	Susceptible	2.2.1
ERR718324	NA	NA	Other	2.2.1.2
ERR718325	NA	NA	Other	2.2.1
ERR718330	NA	NA	Susceptible	2.2.1
ERR718337	NA	NA	Susceptible	2.2.1

ERR718338	NA	NA	Susceptible	2.2.1
ERR718345	NA	NA	Other	2.2.1
ERR718347	NA	NA	Susceptible	2.2.1
ERR718350	NA	NA	Susceptible	2.2.1
ERR718351	NA	NA	Susceptible	2.2.1
ERR718352	NA	NA	Susceptible	2.2.1
ERR718355	NA	NA	Other	2.2.2
ERR718357	NA	NA	Other	2.2.1
ERR718359	NA	NA	Susceptible	2.2.1
ERR718364	NA	NA	Other	2.2.1
ERR718365	NA	NA	Other	2.2.1
ERR718368	NA	NA	Susceptible	2.2.1
ERR718369	NA	NA	Other	2.2.1
ERR718372	NA	NA	Susceptible	2.2.1
ERR718373	NA	NA	Other	2.2.1
ERR718377	NA	NA	Susceptible	2.2.1
ERR718378	NA	NA	Susceptible	2.2.1
ERR718379	NA	NA	Susceptible	2.2.1
ERR718383	NA	NA	Susceptible	2.2.1
ERR718385	NA	NA	Susceptible	2.2.1
ERR718392	NA	NA	Susceptible	2.2.1
ERR718393	NA	NA	Susceptible	2.2.1
ERR718396	NA	NA	Susceptible	2.2.1
ERR718399	NA	NA	Susceptible	2.2.1
ERR718401	NA	NA	Other	2.2.1
ERR718403	NA	NA	Susceptible	2.2.1
ERR718405	NA	NA	Other	2.2.1
ERR718409	NA	NA	Susceptible	2.2.1
ERR718411	NA	NA	Susceptible	2.2.1
ERR718412	NA	NA	Susceptible	2.2.2
ERR718413	NA	NA	Susceptible	2.2.1
ERR718414	NA	NA	Other	2.2.1
ERR718416	NA	NA	Susceptible	2.2.1
ERR718417	NA	NA	Susceptible	2.2.1
ERR718420	NA	NA	Other	2.2.1
ERR718425	NA	NA	Susceptible	2.2.1
ERR718426	NA	NA	Susceptible	2.2.1
ERR718429	NA	NA	Susceptible	2.2.1.1
ERR718430	NA	NA	Other	2.2.1
ERR718431	NA	NA	Susceptible	2.2.1
ERR718432	NA	NA	Susceptible	2.2.1
ERR718435	NA	NA	Susceptible	2.2.1
ERR718440	NA	NA	Susceptible	2.2.1
ERR718444	NA	NA	Susceptible	2.2.1
ERR718446	NA	NA	Susceptible	2.2.1
ERR718454	NA	NA	Susceptible	2.2.1
ERR718455	NA	NA	Susceptible	2.2.1
ERR718456	NA	NA	Other	2.2.1
ERR718457	NA	NA	Susceptible	2.2.1
ERR718459	NA	NA	Other	2.2.1
ERR718460	NA	NA	Other	2.2.1
ERR718462	NA	NA	Other	2.2.1
ERR718469	NA	NA	Susceptible	2.2.1
ERR718474	NA	NA	Susceptible	2.2.1
ERR718475	NA	NA	Susceptible	2.2.1
ERR718476	NA	NA	Susceptible	2.2.1
ERR718478	NA	NA	Susceptible	2.2.1
ERR718479	NA	NA	Susceptible	2.2.1
ERR718482	NA	NA	Susceptible	2.2.1
ERR718483	NA	NA	Susceptible	2.2.1
ERR718484	NA	NA	Other	2.2.1

ERR718486	NA	NA	Susceptible	2.2.1
ERR718490	NA	NA	Susceptible	2.2.1
ERR718492	NA	NA	Susceptible	2.2.1
ERR718493	NA	NA	MDR	2.2.1
ERR718496	NA	NA	Susceptible	2.2.1
ERR718498	NA	NA	Susceptible	2.2.1
ERR718500	NA	NA	Other	2.2.1
ERR718503	NA	NA	Susceptible	2.2.1
ERR718504	NA	NA	MDR	2.2.1
ERR718505	NA	NA	Susceptible	2.2.1
ERR718506	NA	NA	Susceptible	2.2.1
ERR718507	NA	NA	Other	2.2.1
ERR718508	NA	NA	Susceptible	2.2.1
ERR718509	NA	NA	Susceptible	2.2.1
ERR718510	NA	NA	Susceptible	2.2.1
ERR718513	NA	NA	Other	2.2.1
ERR718516	NA	NA	Susceptible	2.2.1
ERR718518	NA	NA	Susceptible	2.2.1
ERR718519	NA	NA	Susceptible	2.2.1
ERR718521	NA	NA	MDR	2.2.1
ERR718524	NA	NA	Susceptible	2.2.1
ERR718525	NA	NA	Susceptible	2.2.1
ERR718526	NA	NA	Susceptible	2.2.1
ERR718528	NA	NA	Susceptible	2.2.1
ERR718530	NA	NA	Susceptible	2.2.1
ERR718535	NA	NA	Other	2.2.1
ERR718537	NA	NA	Susceptible	2.2.1
ERR718540	NA	NA	Susceptible	2.2.2
ERR718541	NA	NA	MDR	2.2.1
ERR718548	NA	NA	Susceptible	2.2.1
ERR718551	NA	NA	Susceptible	2.2.1
ERR718561	NA	NA	Susceptible	2.2.1
ERR718563	NA	NA	Other	2.2.1
ERR736805	NA	NA	Susceptible	2.2.1
ERR736808	NA	NA	Susceptible	2.2.1
ERR751360	Peru	2008	MDR	2.2.1
ERR751361	Peru	2009	MDR	2.2.1
ERR751376	Peru	2009	Susceptible	2.2.1
ERR751378	Peru	2009	MDR	2.2.1
ERR751385	Peru	2009	Other	2.2.1
ERR751390	Peru	2009	Other	2.2.1
ERR751395	Peru	2009	XDR	2.2.1
ERR751419	Peru	2009	Other	2.2.1
ERR751430	Peru	2010	Susceptible	2.2.1
ERR751453	Peru	2012	MDR	2.2.1
ERR751454	Peru	2012	Other	2.2.1
ERR751461	Peru	2012	MDR	2.2.1
ERR751471	Peru	2012	Susceptible	2.2.1
ERR751483	Peru	2008	MDR	2.2.1
ERR751506	Peru	2009	MDR	2.2.1
ERR751522	Peru	2009	MDR	2.2.1
ERR751546	Peru	2011	MDR	2.2.1
ERR751552	Peru	2011	MDR	2.2.1
ERR751594	Peru	2011	MDR	2.2.1
ERR751603	Peru	2007	MDR	2.2.1
ERR751635	NA	NA	Susceptible	2.2.1
ERR751636	NA	NA	Susceptible	2.2.1
ERR751637	NA	NA	Susceptible	2.2.1
ERR751638	NA	NA	Susceptible	2.2.1
ERR751640	NA	NA	Other	2.2.1
ERR751641	NA	NA	Other	2.2.1

ERR751642	NA	NA	MDR	2.2.1
ERR751644	NA	NA	Susceptible	2.2.1
ERR751646	NA	NA	Susceptible	2.2.1
ERR751647	NA	NA	Susceptible	2.2.1
ERR751648	NA	NA	Susceptible	2.2.1
ERR751649	NA	NA	Susceptible	2.2.1
ERR751650	NA	NA	Other	2.2.1
ERR751651	NA	NA	Susceptible	2.2.1
ERR751652	NA	NA	Susceptible	2.2.1.2
ERR751654	NA	NA	Other	2.2.1
ERR751656	NA	NA	Susceptible	2.2.1
ERR751658	NA	NA	Susceptible	2.2.1
ERR751661	NA	NA	Susceptible	2.2.1
ERR751662	NA	NA	Susceptible	2.2.1
ERR751663	NA	NA	Susceptible	2.2.1
ERR751664	NA	NA	Susceptible	2.2.1
ERR751665	NA	NA	Susceptible	2.2.1
ERR751666	NA	NA	Susceptible	2.2.1
ERR751671	NA	NA	Other	2.2.1
ERR751672	NA	NA	Susceptible	2.2.1
ERR751674	NA	NA	Susceptible	2.2.1
ERR751675	NA	NA	Other	2.2.1
ERR751677	NA	NA	Other	2.2.1
ERR751678	NA	NA	Susceptible	2.2.1
ERR751682	NA	NA	Susceptible	2.2.1
ERR751683	NA	NA	Other	2.2.1
ERR751686	NA	NA	Susceptible	2.2.1
ERR751687	NA	NA	Susceptible	2.2.1
ERR751691	NA	NA	Susceptible	2.2.1
ERR751692	NA	NA	Other	2.2.1
ERR751694	NA	NA	Susceptible	2.2.1
ERR751696	NA	NA	Other	2.2.1
ERR751697	NA	NA	Susceptible	2.2.2
ERR751699	NA	NA	Susceptible	2.2.1.1
ERR751700	NA	NA	MDR	2.2.1
ERR751701	NA	NA	Susceptible	2.2.1
ERR751702	NA	NA	Susceptible	2.2.1
ERR751705	NA	NA	Susceptible	2.2.1
ERR751706	NA	NA	Susceptible	2.2.1
ERR751708	NA	NA	Susceptible	2.2.1
ERR751710	NA	NA	Susceptible	2.2.1
ERR751711	NA	NA	Susceptible	2.2.1
ERR751715	NA	NA	Susceptible	2.2.1
ERR751716	NA	NA	Susceptible	2.2.1
ERR751718	NA	NA	Susceptible	2.2.1
ERR751719	NA	NA	Susceptible	2.2.1
ERR751720	NA	NA	Other	2.2.1
ERR751723	NA	NA	Other	2.2.1
ERR751725	NA	NA	Other	2.2.1
ERR751727	NA	NA	Susceptible	2.2.1
ERR751728	NA	NA	Susceptible	2.2.1
ERR751731	NA	NA	Susceptible	2.2.1
ERR751733	NA	NA	Susceptible	2.2.1
ERR751734	NA	NA	Susceptible	2.2.1
ERR751735	NA	NA	Susceptible	2.2.1
ERR751736	NA	NA	Other	2.2.1
ERR751740	NA	NA	MDR	2.2.1
ERR751741	NA	NA	Susceptible	2.2.1
ERR751742	NA	NA	Other	2.2.1
ERR751743	NA	NA	Susceptible	2.2.1
ERR751745	NA	NA	Other	2.2.2

ERR751747	NA	NA	Other	2.2.1
ERR751751	NA	NA	MDR	2.2.1
ERR751753	NA	NA	Susceptible	2.2.1
ERR751755	NA	NA	Susceptible	2.2.1
ERR751761	NA	NA	Susceptible	2.2.1.1
ERR751763	NA	NA	Susceptible	2.2.1
ERR751767	NA	NA	MDR	2.2.1
ERR751768	NA	NA	Susceptible	2.2.1
ERR751770	NA	NA	Other	2.2.1
ERR751773	NA	NA	Susceptible	2.2.1
ERR751774	NA	NA	Susceptible	2.2.1
ERR751775	NA	NA	Other	2.2.1
ERR751776	NA	NA	Susceptible	2.2.1
ERR751781	NA	NA	Other	2.2.1
ERR751784	NA	NA	Susceptible	2.2.1.1
ERR751785	NA	NA	Susceptible	2.2.1
ERR751786	NA	NA	Other	2.2.1
ERR751787	NA	NA	Other	2.2.1
ERR751789	NA	NA	Susceptible	2.2.1
ERR751791	NA	NA	Susceptible	2.2.1
ERR751796	NA	NA	Susceptible	2.2.1
ERR751798	NA	NA	Susceptible	2.2.1
ERR751799	NA	NA	Susceptible	2.2.1
ERR751804	NA	NA	Other	2.2.1
ERR751805	NA	NA	Susceptible	2.2.1
ERR751807	NA	NA	Other	2.2.1
ERR751809	NA	NA	Susceptible	2.2.1
ERR751810	NA	NA	Susceptible	2.2.1
ERR751811	NA	NA	Susceptible	2.2.1
ERR751812	NA	NA	Other	2.2.1
ERR751813	NA	NA	Susceptible	2.2.1
ERR751814	NA	NA	Susceptible	2.2.1
ERR751815	NA	NA	Other	2.2.1
ERR751816	NA	NA	Other	2.2.1
ERR751819	NA	NA	Other	2.2.1
ERR751822	NA	NA	Susceptible	2.2.1
ERR751828	NA	NA	Other	2.2.1
ERR751830	NA	NA	Susceptible	2.2.1
ERR751831	NA	NA	Susceptible	2.2.1.1
ERR751832	NA	NA	Other	2.2.1
ERR751833	NA	NA	Susceptible	2.2.1
ERR751834	NA	NA	Susceptible	2.2.1
ERR751837	NA	NA	MDR	2.2.1
ERR751839	NA	NA	Other	2.2.1
ERR751840	NA	NA	Susceptible	2.2.1
ERR751845	NA	NA	Susceptible	2.2.1
ERR751846	NA	NA	Susceptible	2.2.1
ERR751847	NA	NA	Susceptible	2.2.1
ERR751850	NA	NA	Susceptible	2.2.1
ERR751851	NA	NA	Other	2.2.2
ERR751854	NA	NA	Susceptible	2.2.1
ERR751855	NA	NA	Susceptible	2.2.1
ERR751856	NA	NA	Susceptible	2.2.1
ERR751858	NA	NA	Susceptible	2.2.1
ERR751860	NA	NA	Other	2.2.1
ERR751862	NA	NA	Other	2.2.1
ERR751863	NA	NA	Susceptible	2.2.1
ERR751864	NA	NA	Susceptible	2.2.1
ERR751866	NA	NA	Susceptible	2.2.1
ERR751868	NA	NA	Susceptible	2.2.1
ERR751871	NA	NA	Susceptible	2.2.1.1

ERR751872	NA	NA	Other	2.2.1
ERR751873	NA	NA	Susceptible	2.2.1
ERR751876	NA	NA	Susceptible	2.2.1
ERR751881	NA	NA	Other	2.2.1
ERR751886	NA	NA	Other	2.2.1
ERR751887	NA	NA	Susceptible	2.2.1
ERR751888	NA	NA	Other	2.2.1
ERR751890	NA	NA	Susceptible	2.2.1
ERR751891	NA	NA	Susceptible	2.2.1.1
ERR751893	NA	NA	Susceptible	2.2.1
ERR751896	NA	NA	Susceptible	2.2.1
ERR751898	NA	NA	Susceptible	2.2.1
ERR751901	NA	NA	Susceptible	2.2.1
ERR751902	NA	NA	Susceptible	2.2.1
ERR751903	NA	NA	Susceptible	2.2.1
ERR751904	NA	NA	Other	2.2.1
ERR751908	NA	NA	Susceptible	2.2.1
ERR751911	NA	NA	Susceptible	2.2.1
ERR751912	NA	NA	Susceptible	2.2.1
ERR751917	NA	NA	Susceptible	2.2.1
ERR751920	NA	NA	Susceptible	2.2.1
ERR751926	NA	NA	Susceptible	2.2.1
ERR751933	NA	NA	Susceptible	2.2.2
ERR751934	NA	NA	Other	2.2.1
ERR751936	NA	NA	Susceptible	2.2.1
ERR751939	NA	NA	Susceptible	2.2.1.1
ERR751941	NA	NA	Susceptible	2.2.1
ERR751943	NA	NA	Susceptible	2.2.1
ERR751944	NA	NA	Other	2.2.1
ERR751947	NA	NA	Susceptible	2.2.1.1
ERR751949	NA	NA	Other	2.2.1
ERR751954	NA	NA	MDR	2.2.1
ERR751959	NA	NA	Susceptible	2.2.1
ERR751966	NA	NA	Susceptible	2.2.1
ERR751970	NA	NA	Susceptible	2.2.1
ERR751972	NA	NA	Susceptible	2.2.1
ERR751973	NA	NA	Other	2.2.1
ERR751977	NA	NA	Susceptible	2.2.1
ERR751979	NA	NA	Susceptible	2.2.1
ERR751981	NA	NA	Susceptible	2.2.1
ERR751988	NA	NA	Other	2.2.1
ERR751992	NA	NA	Susceptible	2.2.1
ERR751993	NA	NA	Susceptible	2.1
ERR751995	NA	NA	Susceptible	2.2.1
ERR751997	NA	NA	Susceptible	2.2.1
ERR752010	NA	NA	Susceptible	2.2.1
ERR752015	NA	NA	Other	2.2.1
ERR752016	NA	NA	Other	2.2.1
ERR752020	NA	NA	Susceptible	2.2.1
ERR752023	NA	NA	Susceptible	2.2.1
ERR752024	NA	NA	Susceptible	2.2.1
ERR752025	NA	NA	Other	2.2.1
ERR752027	NA	NA	Other	2.2.1
ERR752029	NA	NA	Susceptible	2.2.1
ERR752030	NA	NA	Susceptible	2.2.1
ERR752032	NA	NA	Susceptible	2.2.1
ERR752033	NA	NA	Susceptible	2.2.1
ERR752034	NA	NA	Susceptible	2.2.1
ERR752036	NA	NA	Susceptible	2.2.1
ERR752038	NA	NA	Susceptible	2.2.1.1
ERR752040	NA	NA	Susceptible	2.2.1

ERR752045	NA	NA	Other	2.2.1
ERR752046	NA	NA	Susceptible	2.2.1
ERR752050	NA	NA	Susceptible	2.2.1
ERR752051	NA	NA	Susceptible	2.2.1
ERR752053	NA	NA	Susceptible	2.2.1
ERR752056	NA	NA	Susceptible	2.2.1
ERR752057	NA	NA	Susceptible	2.2.1
ERR752061	NA	NA	Susceptible	2.2.1.1
ERR752064	NA	NA	Susceptible	2.2.1
ERR752070	NA	NA	Other	2.2.1
ERR752073	NA	NA	Susceptible	2.2.1
ERR752076	NA	NA	Susceptible	2.2.1
ERR752077	NA	NA	Susceptible	2.2.1
ERR752078	NA	NA	Other	2.2.1
ERR752080	NA	NA	Susceptible	2.2.1
ERR752082	NA	NA	Susceptible	2.2.1
ERR752086	NA	NA	Susceptible	2.2.1
ERR752087	NA	NA	Other	2.2.1
ERR752088	NA	NA	Susceptible	2.2.1
ERR752089	NA	NA	Susceptible	2.2.1
ERR752091	NA	NA	Susceptible	2.2.1
ERR752095	NA	NA	Susceptible	2.2.1
ERR752099	NA	NA	Other	2.2.1
ERR752104	NA	NA	Susceptible	2.2.1
ERR752106	NA	NA	Susceptible	2.2.1
ERR752107	NA	NA	Other	2.2.1
ERR752110	NA	NA	Other	2.2.1
ERR752112	NA	NA	Other	2.2.1
ERR752115	NA	NA	Susceptible	2.2.1
ERR752125	NA	NA	Susceptible	2.2.1
ERR752127	NA	NA	Susceptible	2.2.1
ERR752130	NA	NA	Susceptible	2.2.1
ERR752132	NA	NA	Susceptible	2.2.1
ERR752135	NA	NA	Susceptible	2.2.1
ERR752136	NA	NA	Susceptible	2.2.1
ERR752137	NA	NA	Susceptible	2.2.1
ERR752139	NA	NA	Susceptible	2.2.1
ERR752140	NA	NA	Susceptible	2.2.1
ERR752141	NA	NA	Other	2.2.1
ERR752142	NA	NA	Susceptible	2.2.1
ERR752143	NA	NA	Susceptible	2.2.1
ERR752144	NA	NA	Susceptible	2.2.1
ERR752146	NA	NA	Susceptible	2.2.1
ERR752147	NA	NA	Other	2.2.1
ERR752148	NA	NA	Susceptible	2.2.1
ERR752149	NA	NA	MDR	2.2.1.1
ERR752152	NA	NA	Susceptible	2.2.1
ERR752155	NA	NA	Other	2.2.1
ERR752156	NA	NA	Other	2.2.1
ERR752165	NA	NA	Susceptible	2.2.1
ERR752167	NA	NA	Other	2.2.1
ERR752169	NA	NA	Susceptible	2.2.1
ERR752173	NA	NA	Other	2.2.1
ERR752175	NA	NA	Susceptible	2.2.1
ERR752180	NA	NA	Susceptible	2.2.1
ERR752182	NA	NA	Susceptible	2.2.1
ERR752190	NA	NA	Susceptible	2.2.1
ERR752191	NA	NA	MDR	2.2.1
ERR752193	NA	NA	Susceptible	2.2.1
ERR752196	NA	NA	Susceptible	2.2.1.1
ERR752199	NA	NA	MDR	2.2.1

ERR752200	NA	NA	Susceptible	2.2.1
ERR752203	NA	NA	Susceptible	2.2.1
ERR752208	NA	NA	Susceptible	2.2.1
ERR752211	NA	NA	Other	2.2.1
ERR752212	NA	NA	Susceptible	2.2.1
ERR752215	NA	NA	Susceptible	2.2.1
ERR752216	NA	NA	Susceptible	2.2.1
ERR752217	NA	NA	Susceptible	2.2.1
ERR752220	NA	NA	Susceptible	2.2.1
ERR752221	NA	NA	Susceptible	2.2.1
ERR752222	NA	NA	Other	2.2.1
ERR752223	NA	NA	Susceptible	2.2.1
ERR752224	NA	NA	Susceptible	2.2.1
ERR752225	NA	NA	MDR	2.2.1
ERR752227	NA	NA	Susceptible	2.2.1
ERR752228	NA	NA	Other	2.2.1
ERR752231	NA	NA	Susceptible	2.2.1
ERR752232	NA	NA	Susceptible	2.2.1
ERR752234	NA	NA	MDR	2.1
ERR752236	NA	NA	Susceptible	2.2.1
ERR752239	NA	NA	Susceptible	2.2.1
ERR752245	NA	NA	Susceptible	2.2.1
ERR752246	NA	NA	Susceptible	2.2.1
ERR752251	NA	NA	Susceptible	2.2.1
ERR752252	NA	NA	Susceptible	2.2.1
ERR752254	NA	NA	Susceptible	2.2.1
ERR752255	NA	NA	Other	2.2.1
ERR752257	NA	NA	Other	2.2.1
ERR752258	NA	NA	Susceptible	2.2.1
ERR752259	NA	NA	Susceptible	2.2.1
ERR752260	NA	NA	Susceptible	2.2.1
ERR752261	NA	NA	Susceptible	2.2.1
ERR752262	NA	NA	Susceptible	2.1
ERR752264	NA	NA	Other	2.2.1
ERR752265	NA	NA	Susceptible	2.2.1
ERR752266	NA	NA	Susceptible	2.2.1
ERR752269	NA	NA	Susceptible	2.2.1.1
ERR752270	NA	NA	Susceptible	2.2.1
ERR752271	NA	NA	Susceptible	2.2.1
ERR752273	NA	NA	Susceptible	2.2.1
ERR752274	NA	NA	Susceptible	2.2.1
ERR752277	NA	NA	Other	2.2.1
ERR752278	NA	NA	Susceptible	2.2.1
ERR752279	NA	NA	Other	2.2.1
ERR752281	NA	NA	Susceptible	2.2.1
ERR752282	NA	NA	Susceptible	2.2.1
ERR752283	NA	NA	Other	2.2.1
ERR752286	NA	NA	Susceptible	2.2.1
ERR752287	NA	NA	Other	2.2.1
ERR752288	NA	NA	Susceptible	2.2.1
ERR767959	NA	NA	Susceptible	2.2.1
ERR767960	NA	NA	Susceptible	2.2.1
ERR767969	NA	NA	Susceptible	2.2.1
ERR767970	NA	NA	Susceptible	2.2.1
ERR767973	NA	NA	Susceptible	2.2.1.1
ERR767974	NA	NA	Susceptible	2.2.1
ERR767978	NA	NA	Susceptible	2.2.1
ERR767982	NA	NA	Other	2.2.1
ERR767983	NA	NA	Susceptible	2.2.1
ERR767984	NA	NA	Other	2.2.1
ERR767988	NA	NA	Susceptible	2.2.1

ERR767989	NA	NA	Susceptible	2.2.1
ERR767990	NA	NA	Susceptible	2.2.1
ERR767994	NA	NA	Other	2.2.1
ERR767996	NA	NA	Susceptible	2.2.1
ERR768002	NA	NA	Other	2.2.1
ERR768004	NA	NA	Susceptible	2.2.1
ERR768017	NA	NA	Susceptible	2.2.1
ERR768020	NA	NA	Susceptible	2.2.1
ERR768023	NA	NA	Susceptible	2.2.1
ERR768026	NA	NA	Susceptible	2.2.1
ERR768028	NA	NA	Other	2.2.1
ERR768029	NA	NA	Other	2.2.1.1
ERR768031	NA	NA	Susceptible	2.2.1
ERR768032	NA	NA	Other	2.1
ERR768034	NA	NA	Susceptible	2.2.1
ERR768036	NA	NA	Susceptible	2.2.1
ERR768040	NA	NA	Susceptible	2.2.1
ERR768042	NA	NA	Susceptible	2.2.1
ERR768043	NA	NA	Susceptible	2.2.1
ERR768045	NA	NA	Susceptible	2.2.1
ERR768046	NA	NA	Other	2.2.1
ERR768047	NA	NA	Other	2.2.1
ERR768048	NA	NA	Susceptible	2.2.1
ERR768051	NA	NA	Susceptible	2.2.1
ERR773792	NA	NA	Susceptible	2.2.1
ERR773807	NA	NA	Susceptible	2.2.1
ERR775346	Peru	2009	Other	2.2.1
ERR775347	Peru	2009	Other	2.2.1
ERR775370	Peru	2011	Susceptible	2.2.1
ERR775373	Peru	2011	MDR	2.2.1
ERR775375	Peru	2011	MDR	2.2.1
ERR775378	Peru	2011	MDR	2.2.1
ERR775381	Peru	2011	MDR	2.2.1
ERR775382	Peru	2011	MDR	2.2.1
ERR775753	NA	NA	Other	2.2.1
ERR775756	NA	NA	Susceptible	2.2.1
ERR775757	NA	NA	Susceptible	2.2.1
ERR775761	NA	NA	Susceptible	2.1
ERR775763	NA	NA	Susceptible	2.2.1
ERR775764	NA	NA	Susceptible	2.2.1.1
ERR775766	NA	NA	Other	2.1
ERR775767	NA	NA	Susceptible	2.2.1
ERR775769	NA	NA	Other	2.2.1
ERR775771	NA	NA	Susceptible	2.2.1
ERR775772	NA	NA	Susceptible	2.2.1
ERR775773	NA	NA	Susceptible	2.1
ERR775775	NA	NA	Susceptible	2.2.1
ERR775784	NA	NA	MDR	2.1
ERR775787	NA	NA	Susceptible	2.1
ERR775788	NA	NA	Susceptible	2.2.1
ERR775791	NA	NA	Susceptible	2.1
ERR775793	NA	NA	Susceptible	2.2.1
ERR775794	NA	NA	MDR	2.1
ERR775799	NA	NA	Other	2.2.1
ERR779843	Peru	2009	Other	2.2.1
ERR779845	Peru	2009	MDR	2.2.1
ERR779851	Peru	2009	MDR	2.2.1
ERR779894	Peru	2011	MDR	2.2.1
ERR779910	Peru	2011	MDR	2.2.1
ERR779914	Peru	2011	Other	2.2.1
ERR789234	Switzerland	NA	MDR	2.2.1

ERR789235	Switzerland	NA	MDR	2.2.1
ERR789236	Switzerland	NA	MDR	2.2.1
ERR789237	Switzerland	NA	MDR	2.2.1
ERR789238	Switzerland	NA	MDR	2.2.1
ERR789239	Switzerland	NA	MDR	2.2.1
ERR841493	NA	NA	Susceptible	2.1
ERR845932	NA	NA	MDR	2.2.1
ERR846992	NA	NA	Susceptible	2.2.1
ERR846993	NA	NA	Susceptible	2.2.1
ERR846997	NA	NA	MDR	2.2.1
ERR867521	United Kingdom	04-01-2012	MDR	2.2.1
ERR867522	United Kingdom	05-01-2012	Other	2.2.1
ERR867523	United Kingdom	06-01-2012	Other	2.2.1
ERR867525	United Kingdom	08-01-2012	Other	2.2.1
ERR867526	United Kingdom	09-01-2012	MDR	2.2.1
ERR867527	United Kingdom	10-01-2012	Other	2.2.1
ERR867531	United Kingdom	14-01-2012	MDR	2.2.1
ERR867532	United Kingdom	15-01-2012	MDR	2.2.1
ERR867533	United Kingdom	16-01-2012	MDR	2.2.1
ERR867535	United Kingdom	18-01-2012	MDR	2.2.1
ERR867536	United Kingdom	19-01-2012	MDR	2.2.1
ERR867537	United Kingdom	20-01-2012	MDR	2.2.1
ERR867540	United Kingdom	23-01-2012	Other	2.2.1
ERR867548	United Kingdom	31-01-2012	MDR	2.2.1
ERR867556	United Kingdom	2012-01-39	Susceptible	2.2.2
ERR867562	United Kingdom	2012-01-45	Susceptible	2.2.2
ERR970413	NA	NA	MDR	2.2.1
ERR970414	NA	NA	Other	2.2.1.1
ERR970441	NA	NA	Other	2.2.1.1
ERR970442	NA	NA	Other	2.2.1.1
ERR970443	NA	NA	Other	2.2.1.1
ERR970444	NA	NA	Other	2.2.1.1
ERR970445	NA	NA	MDR	2.2.1
ERR970447	NA	NA	MDR	2.2.1
ERR970456	NA	NA	Other	2.2.1.1
ERR970460	NA	NA	Other	2.2.1
ERR970461	NA	NA	Other	2.2.1
ERR970462	NA	NA	Other	2.2.1
ERR970463	NA	NA	Other	2.2.1.1
ERR972795	NA	NA	MDR	2.2.1
ERR972800	NA	NA	Other	2.2.1
ERR972801	NA	NA	Other	2.2.1.1
ERR972803	NA	NA	Other	2.2.1
ERR972804	NA	NA	Other	2.2.1.1
ERR983228	Myanmar	2013	MDR	2.2.1
ERR983229	Myanmar	2013	MDR	2.2.1.1
ERR983230	Myanmar	2013	MDR	2.2.1.1
ERR983231	Myanmar	2013	MDR	2.2.1.1
ERR983234	Myanmar	2013	MDR	2.2.1
ERR983236	Myanmar	2013	MDR	2.2.1
ERR983238	Myanmar	2013	MDR	2.2.1.1
ERR983239	Myanmar	2013	MDR	2.2.1
ERR983240	Myanmar	2013	MDR	2.2.1
ERR983241	Myanmar	2013	MDR	2.2.1
ERR983255	NA	NA	Other	2.2.1.1
ERR983256	NA	NA	Other	2.2.1.1
ERR985422	NA	NA	Other	2.2.1.1
ERR985423	NA	NA	Other	2.2.1
ERR985424	NA	NA	Other	2.2.1.1
ERR985425	NA	NA	Other	2.2.1.1
ERR987714	Uganda	NA	Susceptible	2.2.1

ERR987746	Uganda	NA	MDR	2.2.1
ERR987756	Uganda	NA	MDR	2.2.1
ERR987760	Uganda	NA	MDR	2.2.1
ERR987768	Uganda	NA	Susceptible	2.2.1
H37Rv	NA	NA	Susceptible	NA
SRR023449	NA	NA	Other	2.2.1
SRR023450	NA	NA	Other	2.2.1
SRR023451	NA	NA	Other	2.2.1
SRR023477	NA	NA	Other	2.2.1
SRR023487	NA	NA	Other	2.2.1
SRR023492	NA	NA	Susceptible	2.2.1
SRR026442	NA	NA	MDR	2.2.1
SRR1187296	Canada	2011	Susceptible	2.2.1.2
SRR1200797	2009	May 6	Susceptible	Canada
SRR1200798	NA	missing	Susceptible	2.2.1.1
SRR1200811	2009	May 6	Susceptible	Canada
SRR1367196	China	not collected	MDR	2.2.1
SRR1367197	China	not collected	MDR	2.2.1
SRR1367216	China	not collected	MDR	2.2.1
SRR1367217	China	not collected	MDR	2.2.1
SRR1367219	China	not collected	XDR	2.2.1
SRR1367220	China	not collected	Susceptible	2.2.1
SRR1367221	China	not collected	Other	2.2.1
SRR1367225	China	not collected	Susceptible	2.2.1
SRR1367226	China	not collected	XDR	2.2.1
SRR1368332	China	not collected	XDR	2.2.1
SRR1368333	China	not collected	MDR	2.2.1
SRR1368334	China	not collected	MDR	2.2.1
SRR1368335	China	not collected	MDR	2.2.1
SRR1368336	China	not collected	MDR	2.2.1
SRR1368337	China	not collected	XDR	2.2.1
SRR1368338	China	not collected	XDR	2.2.1
SRR1368339	China	not collected	XDR	2.2.1
SRR1368340	China	not collected	XDR	2.2.1
SRR1368341	China	not collected	Other	2.2.1
SRR1564305	Thailand	26-Aug-2003	MDR	2.2.1
SRR1577806	Mali	2008-06-20T00:00:00	MDR	2.2.1
SRR1577808	Mali	2010-03-08T00:00:00	Other	2.2.1
SRR1577822	Mali	2010-03-08T00:00:00	Other	2.2.1
SRR1577830	Mali	2008-06-20T00:00:00	MDR	2.2.1
SRR1595970	Thailand	18-mar-08	MDR	2.2.1
SRR1595971	Thailand	18-mar-08	MDR	2.2.1
SRR1595972	Thailand	11-mar-08	MDR	2.2.1
SRR1710057	China	06-nov-10	Susceptible	2.1
SRR1710058	China	27-jul-10	Susceptible	2.1
SRR1710059	China	12-jan-11	Susceptible	2.1
SRR1710060	China	12-Aug-2010	Susceptible	2.1
SRR1710061	China	12-jul-10	Susceptible	2.1
SRR1710062	China	26-Aug-1973	Other	2.1
SRR1710063	China	09-Feb-1922	Susceptible	2.1
SRR1710064	China	10-Sep-2010	Susceptible	2.1
SRR1710065	China	03-Oct-2010	Susceptible	2.1
SRR1710066	China	04-Sep-2010	Susceptible	2.1
SRR1710067	China	29-Apr-2011	Susceptible	2.1
SRR1710068	China	22-jan-11	Susceptible	2.1
SRR1710069	China	08-Oct-2010	Susceptible	2.1
SRR1710070	China	06-Sep-2010	Susceptible	2.1
SRR1710071	China	26-Sep-2010	Other	2.1
SRR1710072	China	01-Aug-2010	Susceptible	2.1
SRR1710073	China	21-jan-08	Susceptible	2.2.1
SRR1710074	China	05-Feb-2009	Susceptible	2.2.2

SRR1710075	China	16-jan-09	Susceptible	2.2.1
SRR1710076	China	19-mar-09	MDR	2.2.2
SRR1710077	China	03-jun-09	Susceptible	2.2.2
SRR1710078	China	17-jun-09	Susceptible	2.2.1
SRR1710079	China	23-Sep-2009	Susceptible	2.2.2
SRR1710080	China	21-Aug-2009	Susceptible	2.2.1
SRR1710081	China	21-Sep-2009	Susceptible	2.2.1
SRR1710082	China	27-Oct-2009	Susceptible	2.2.1
SRR1710083	China	08-Apr-2010	Susceptible	2.2.2
SRR1710084	China	31-mar-10	Susceptible	2.2.1
SRR1710085	China	12-May-2010	Susceptible	2.2.1
SRR1710086	China	24-May-2010	Susceptible	2.2.1
SRR1710087	China	07-jun-10	Susceptible	2.2.1
SRR1710089	China	23-nov-10	Susceptible	2.2.1
SRR1710090	China	11-jun-10	Other	2.2.1
SRR1710091	China	28-jan-11	Susceptible	2.2.2
SRR1710092	China	20-jan-11	Susceptible	2.2.1
SRR1710093	China	30-mar-11	Other	2.2.2
SRR1710094	China	04-jun-11	Susceptible	2.2.1
SRR1710095	China	17-Sep-2010	Susceptible	2.2.2
SRR1710096	China	18-jun-10	MDR	2.2.1
SRR1710097	China	13-Sep-2010	Susceptible	2.2.1
SRR1710098	China	26-Oct-2010	Susceptible	2.2.1
SRR1710099	China	01-jun-10	MDR	2.2.1
SRR1710100	China	20-jul-10	Susceptible	2.2.1
SRR1710101	China	04-Oct-2010	Susceptible	2.2.1
SRR1710102	China	28-mar-11	Susceptible	2.2.2
SRR1710103	China	10-May-2010	Susceptible	2.2.1
SRR1710104	China	11-mar-10	Susceptible	2.2.1
SRR1710105	China	20-jan-10	Susceptible	2.2.2
SRR1710106	China	02-mar-10	Susceptible	2.2.1
SRR1710107	China	26-mar-12	Susceptible	2.2.1
SRR1710108	China	19-Feb-2010	Other	2.2.1
SRR1710109	China	14-jul-10	Susceptible	2.2.1
SRR1710110	China	14-Feb-2011	Susceptible	2.2.1
SRR1710111	China	29-Apr-2009	Other	2.2.2
SRR1710112	China	16-jul-09	Other	2.2.1
SRR1735581	United Kingdom	29-jun-13	Susceptible	2.2.1
SRR1735582	United Kingdom	02-jul-13	Susceptible	2.2.1
SRR1735586	United Kingdom	25-jul-13	Susceptible	2.2.1
SRR1735588	United Kingdom	17-jul-13	Susceptible	2.2.1
SRR1765871	Guatemala	2010	Susceptible	2.2.1
SRR1765872	Guatemala	2010	Susceptible	2.2.1
SRR1765874	Guatemala	2011	Susceptible	2.2.1
SRR1765877	Guatemala	2012	Susceptible	2.2.1
SRR1765879	Guatemala	2011	Susceptible	2.2.1
SRR2010299	China	2011	Other	2.2.1
SRR2024879	China	23-Dec-2008	MDR	2.2.1
SRR2024880	China	23-Dec-2008	MDR	2.2.1
SRR2024881	China	06-May-2009	MDR	2.2.1
SRR2024882	China	07-mar-12	MDR	2.2.1
SRR2024883	China	19-mar-12	MDR	2.2.1
SRR2024884	China	11-Apr-2012	MDR	2.2.1
SRR2024885	China	11-Apr-2012	MDR	2.2.1
SRR2024886	China	20-mar-12	MDR	2.2.1
SRR2024887	China	16-Apr-2012	MDR	2.2.1
SRR2024888	China	18-Apr-2012	MDR	2.2.1
SRR2024889	China	23-Apr-2012	MDR	2.2.1
SRR2024890	China	09-May-2012	MDR	2.2.1.1
SRR2024891	China	11-May-2012	MDR	2.2.1
SRR2024892	China	08-May-2009	MDR	2.2.1

SRR2024893	China	16-May-2012	MDR	2.2.2
SRR2024894	China	26-jun-12	MDR	2.2.1
SRR2024895	China	26-jun-12	MDR	2.2.1
SRR2024897	China	28-Aug-2012	MDR	2.2.1
SRR2024898	China	03-Sep-2012	MDR	2.2.1
SRR2024899	China	26-Sep-2012	XDR	2.2.1
SRR2024900	China	11-Oct-2012	MDR	2.2.1
SRR2024902	China	08-Oct-2012	Other	2.2.1
SRR2024904	China	05-nov-12	MDR	2.2.1
SRR2024905	China	21-nov-12	MDR	2.2.1
SRR2024906	China	02-jun-09	MDR	2.2.1
SRR2024907	China	03-jun-09	MDR	2.2.2
SRR2024908	China	27-May-2009	MDR	2.2.1
SRR2024909	China	22-jun-09	MDR	2.2.1
SRR2024910	China	29-jun-09	MDR	2.2.1
SRR2024911	China	19-jun-09	MDR	2.2.1
SRR2024912	China	08-jul-09	MDR	2.2.1.1
SRR2024913	China	14-jan-09	MDR	2.2.1
SRR2024915	China	15-jul-09	MDR	2.2.1
SRR2024916	China	15-jul-09	MDR	2.2.1
SRR2024917	China	29-jul-09	XDR	2.2.1
SRR2024918	China	12-Aug-2009	MDR	2.2.1
SRR2024919	China	18-Aug-2009	MDR	2.2.1
SRR2024920	China	25-Aug-2009	MDR	2.2.1
SRR2024921	China	17-Sep-2009	MDR	2.2.1
SRR2024922	China	23-Sep-2009	MDR	2.2.1
SRR2024923	China	23-Sep-2009	MDR	2.2.2
SRR2024924	China	18-Feb-2009	MDR	2.2.1
SRR2024925	China	12-Oct-2009	MDR	2.2.1
SRR2024926	China	14-Oct-2009	XDR	2.2.1
SRR2024927	China	02-nov-09	MDR	2.2.1
SRR2024928	China	09-nov-09	MDR	2.2.1
SRR2024929	China	11-nov-09	MDR	2.2.1
SRR2024930	China	25-nov-09	MDR	2.2.1
SRR2024931	China	17-Dec-2009	Other	2.2.1
SRR2024932	China	28-Dec-2009	MDR	2.2.1
SRR2024933	China	06-jan-10	MDR	2.2.1
SRR2024934	China	11-jan-10	MDR	2.2.1
SRR2024935	China	18-Feb-2009	MDR	2.2.1
SRR2024936	China	21-jan-10	MDR	2.2.1
SRR2024938	China	08-Feb-2010	MDR	2.2.1
SRR2024939	China	24-Feb-2010	MDR	2.2.1
SRR2024940	China	24-Feb-2010	MDR	2.2.1
SRR2024941	China	24-Feb-2010	XDR	2.2.1
SRR2024942	China	29-mar-10	MDR	2.2.1
SRR2024943	China	08-Apr-2010	MDR	2.2.1.1
SRR2024944	China	15-Apr-2010	MDR	2.2.1
SRR2024945	China	20-Apr-2010	MDR	2.2.1
SRR2024946	China	08-Apr-2009	MDR	2.2.1
SRR2024947	China	23-Apr-2010	Other	2.2.1
SRR2024948	China	21-Apr-2010	MDR	2.2.1
SRR2024950	China	11-May-2010	MDR	2.2.1
SRR2024951	China	12-May-2010	MDR	2.2.1
SRR2024952	China	25-May-2010	MDR	2.2.1
SRR2024953	China	28-May-2010	MDR	2.2.1
SRR2024954	China	23-jun-10	MDR	2.2.1
SRR2024955	China	25-jun-10	XDR	2.2.1
SRR2024956	China	08-jul-10	MDR	2.2.1
SRR2024957	China	22-Apr-2009	MDR	2.2.1
SRR2024958	China	13-jul-10	MDR	2.2.1
SRR2024959	China	25-Aug-2010	MDR	2.2.1

SRR2024960	China	20-Aug-2010	MDR	2.2.1
SRR2024961	China	30-Aug-2010	XDR	2.2.1
SRR2024962	China	08-Sep-2010	MDR	2.2.2
SRR2024963	China	14-Sep-2010	MDR	2.2.1
SRR2024964	China	14-Sep-2010	MDR	2.2.1
SRR2024965	China	20-Sep-2010	MDR	2.2.1
SRR2024966	China	15-Sep-2010	MDR	2.2.1
SRR2024967	China	06-Oct-2010	MDR	2.2.1
SRR2024968	China	29-Apr-2009	MDR	2.2.1
SRR2024969	China	25-Oct-2010	MDR	2.2.1
SRR2024970	China	03-nov-10	Susceptible	2.2.1
SRR2024971	China	08-nov-10	MDR	2.2.1
SRR2024972	China	15-nov-10	MDR	2.2.1
SRR2024973	China	29-nov-10	MDR	2.2.1
SRR2024974	China	01-Dec-2010	MDR	2.2.1
SRR2024975	China	08-Dec-2010	MDR	2.2.1
SRR2024976	China	17-Dec-2010	MDR	2.2.1
SRR2024977	China	11-jan-11	MDR	2.2.1
SRR2024978	China	31-jan-11	MDR	2.2.1
SRR2024979	China	29-Apr-2009	MDR	2.2.1
SRR2024980	China	26-jan-11	MDR	2.2.1.1
SRR2024981	China	01-mar-11	MDR	2.2.1
SRR2024982	China	28-Feb-2011	MDR	2.2.1
SRR2024983	China	08-mar-11	MDR	2.2.1
SRR2024984	China	15-mar-11	MDR	2.2.1
SRR2024985	China	03-mar-11	MDR	2.2.1
SRR2024986	China	28-mar-11	MDR	2.2.1.1
SRR2024987	China	24-Apr-2011	MDR	2.2.1
SRR2024988	China	24-May-2011	Other	2.2.1
SRR2024989	China	11-jun-11	MDR	2.2.1
SRR2024990	China	29-Apr-2009	MDR	2.2.1
SRR2024991	China	18-May-2011	MDR	2.2.1.1
SRR2024992	China	13-jun-11	MDR	2.2.1
SRR2024993	China	11-Aug-2011	MDR	2.2.1
SRR2024994	China	01-Sep-2011	MDR	2.2.1
SRR2024995	China	17-Oct-2011	MDR	2.2.1
SRR2024996	China	21-nov-11	MDR	2.2.1
SRR2024997	China	12-Dec-2011	MDR	2.2.1
SRR2024998	China	30-Dec-2011	MDR	2.2.1
SRR2024999	China	17-jan-12	Other	2.2.1
SRR2025000	China	02-Feb-2012	MDR	2.2.1
SRR2062305	China	11-jan-11	Other	2.2.2
SRR2063840	China	28-Dec-2010	Other	2.2.2
SRR2063842	China	24-jan-11	Other	2.2.2
SRR2063843	China	20-Feb-2011	Susceptible	2.2.2
SRR2063844	China	24-Feb-2011	Other	2.2.2
SRR2075817	Korea	2013	XDR	2.2.2
SRR2075818	Korea	2013	XDR	2.2.2
SRR2075820	Korea	2013	XDR	2.2.2
SRR2075822	Korea	2013	XDR	2.2.2
SRR2075823	Korea	2013	XDR	2.2.2
SRR2099943	NA	missing	Susceptible	2.2.1
SRR2099969	NA	missing	MDR	2.2.1
SRR2099982	NA	missing	XDR	2.2.2
SRR2100026	NA	missing	MDR	2.2.1
SRR2100034	NA	missing	Susceptible	2.2.1
SRR2100056	NA	missing	Susceptible	2.2.1
SRR2100060	NA	missing	Susceptible	2.2.1
SRR2100165	NA	missing	Susceptible	2.2.1
SRR2100182	NA	missing	Susceptible	2.2.1
SRR2100203	NA	missing	Susceptible	2.2.1

SRR2100227	NA	missing	MDR	2.2.1
SRR2100250	NA	missing	XDR	2.2.2
SRR2100257	NA	missing	MDR	2.2.1
SRR2100267	NA	missing	MDR	2.2.1
SRR2100285	NA	missing	Other	2.2.1
SRR2100306	NA	missing	MDR	2.2.1
SRR2100322	NA	missing	Susceptible	2.2.1
SRR2100333	NA	missing	Susceptible	2.2.1
SRR2100335	NA	missing	Other	2.2.1
SRR2100367	NA	missing	Other	2.2.1
SRR2100373	NA	missing	Susceptible	2.2.1
SRR2100387	NA	missing	MDR	2.2.1
SRR2100390	NA	missing	Other	2.2.1
SRR2100391	NA	missing	Other	2.2.1
SRR2100419	NA	missing	XDR	2.2.1
SRR2100429	NA	missing	MDR	2.2.1
SRR2100432	NA	missing	MDR	2.2.1
SRR2100435	NA	missing	Susceptible	2.2.1
SRR2100469	NA	missing	Other	2.2.1
SRR2100490	NA	missing	Other	2.2.1
SRR2100536	NA	missing	Susceptible	2.2.1
SRR2100541	NA	missing	Other	2.2.1
SRR2100543	NA	missing	Other	2.2.1
SRR2100549	NA	missing	Susceptible	2.2.1
SRR2100559	NA	missing	Other	2.2.1
SRR2100580	NA	missing	Susceptible	2.2.1
SRR2100591	NA	missing	Susceptible	2.2.1
SRR2100623	NA	missing	Susceptible	2.2.1.1
SRR2100627	NA	missing	Susceptible	2.2.1
SRR2100670	NA	missing	Other	2.2.1
SRR2100680	NA	missing	Susceptible	2.2.1
SRR2100686	NA	missing	Susceptible	2.2.1
SRR2100704	NA	missing	Susceptible	2.2.1
SRR2100709	NA	missing	Susceptible	2.2.1
SRR2100724	NA	missing	Susceptible	2.2.1.1
SRR2100733	NA	missing	Susceptible	2.2.1
SRR2100740	NA	missing	MDR	2.2.1
SRR2100757	NA	missing	Susceptible	2.2.1
SRR2100769	NA	missing	Other	2.2.1
SRR2100794	NA	missing	Susceptible	2.2.1.1
SRR2100828	NA	missing	MDR	2.2.2
SRR2100830	NA	missing	XDR	2.2.1
SRR2100870	NA	missing	Other	2.2.1
SRR2100873	NA	missing	Other	2.2.1
SRR2100874	NA	missing	Other	2.2.1
SRR2100880	NA	missing	Other	2.2.1
SRR2100889	NA	missing	Other	2.2.1
SRR2100909	NA	missing	MDR	2.2.1
SRR2100911	NA	missing	Other	2.2.1
SRR2100950	NA	missing	MDR	2.2.1
SRR2101008	NA	missing	Susceptible	2.2.1.1
SRR2101053	NA	missing	XDR	2.2.1
SRR2101055	NA	missing	MDR	2.2.1
SRR2101066	NA	missing	Susceptible	2.2.1
SRR2101085	NA	missing	Susceptible	2.2.1
SRR2101110	NA	missing	Other	2.2.1
SRR2101126	NA	missing	Susceptible	2.2.1
SRR2101149	NA	missing	Other	2.2.1
SRR2101178	NA	missing	Susceptible	2.2.1
SRR2101233	NA	missing	Other	2.2.1.1
SRR2101257	NA	missing	Susceptible	2.2.1

SRR2101265	NA	missing	Susceptible	2.2.1
SRR2101279	NA	missing	Susceptible	2.2.1
SRR2101284	NA	missing	Susceptible	2.2.1
SRR2101297	NA	missing	Susceptible	2.2.1
SRR2101298	NA	missing	Susceptible	2.2.1
SRR2101301	NA	missing	Susceptible	2.2.1
SRR2101304	NA	missing	Susceptible	2.2.1
SRR2101309	NA	missing	Other	2.2.1
SRR2101314	NA	missing	Susceptible	2.2.1
SRR2101320	NA	missing	Susceptible	2.2.1
SRR2101321	NA	missing	Susceptible	2.2.1
SRR2101323	NA	missing	Susceptible	2.2.1
SRR2101326	NA	missing	Susceptible	2.2.1.1
SRR2101332	NA	missing	Susceptible	2.2.1
SRR2101335	NA	missing	Susceptible	2.2.1
SRR2101338	NA	missing	Susceptible	2.2.1
SRR2101344	NA	missing	MDR	2.2.2
SRR2101365	NA	missing	Susceptible	2.2.1
SRR2101366	NA	missing	Other	2.2.1
SRR2101374	NA	missing	Susceptible	2.2.1
SRR2101383	NA	missing	Susceptible	2.2.1
SRR2101384	NA	missing	MDR	2.2.2
SRR2101392	NA	missing	Susceptible	2.2.1
SRR2101401	NA	missing	Susceptible	2.2.1
SRR2101402	NA	missing	Susceptible	2.2.1
SRR2101411	NA	missing	Susceptible	2.2.1
SRR2101412	NA	missing	Susceptible	2.2.1
SRR2101413	NA	missing	Susceptible	2.2.1
SRR2101415	NA	missing	Susceptible	2.2.1
SRR2101428	NA	missing	Susceptible	2.2.1
SRR2101439	NA	missing	Susceptible	2.2.1.1
SRR2101444	NA	missing	Susceptible	2.2.1
SRR2101448	NA	missing	Susceptible	2.2
SRR2101452	NA	missing	Susceptible	2.2.1
SRR2101456	NA	missing	Susceptible	2.2.1
SRR2101460	NA	missing	MDR	2.2.1
SRR2101461	NA	missing	Susceptible	2.2.1
SRR2101467	NA	missing	Susceptible	2.2.1
SRR2101470	NA	missing	MDR	2.2.1.1
SRR2101502	NA	missing	Susceptible	2.2.1
SRR2101513	NA	missing	Susceptible	2.2.1
SRR2101518	NA	missing	Susceptible	2.2.1
SRR2101524	NA	missing	Susceptible	2.2.1
SRR2101530	NA	missing	Other	2.2.1
SRR2101531	NA	missing	Susceptible	2.2.1
SRR2101533	NA	missing	Susceptible	2.2.1
SRR2101535	NA	missing	Susceptible	2.2.1
SRR2101542	NA	missing	Susceptible	2.2.1
SRR2101543	NA	missing	Susceptible	2.2.1
SRR2101548	NA	missing	Susceptible	2.2.1
SRR2101567	NA	missing	Susceptible	2.2.1.1
SRR2101570	NA	missing	Susceptible	2.2.1
SRR2101579	NA	missing	Susceptible	2.2.1
SRR2101584	NA	missing	Susceptible	2.2.1
SRR2101586	NA	missing	Susceptible	2.2.2
SRR2101587	NA	missing	Susceptible	2.2.1
SRR2101589	NA	missing	Susceptible	2.2.1
SRR2101601	NA	missing	Susceptible	2.2.1
SRR2101605	NA	missing	Susceptible	2.2.1
SRR2101610	NA	missing	Susceptible	2.2.1
SRR2101615	NA	missing	Susceptible	2.2.1

SRR2101620	NA	missing	Susceptible	2.2.1
SRR2101629	NA	missing	Susceptible	2.2.1
SRR2101631	NA	missing	Susceptible	2.2.1
SRR2101633	NA	missing	Susceptible	2.2.1
SRR2101638	NA	missing	Susceptible	2.2.1
SRR2101643	NA	missing	Other	2.2.1
SRR2101645	NA	missing	Susceptible	2.2.1
SRR2101655	NA	missing	Susceptible	2.2.1
SRR2101660	NA	missing	Susceptible	2.2.1.1
SRR2101665	NA	missing	Susceptible	2.2.1
SRR2101667	NA	missing	Susceptible	2.2.1
SRR2101669	NA	missing	Susceptible	2.2.1
SRR2101672	NA	missing	Susceptible	2.2.1
SRR2101676	NA	missing	Susceptible	2.2.1
SRR2101677	NA	missing	Susceptible	2.2.1
SRR2101691	NA	missing	Susceptible	2.2.1
SRR2101695	NA	missing	Susceptible	2.2.1.1
SRR2101707	NA	missing	Susceptible	2.2.1
SRR2101708	NA	missing	Other	2.2.1
SRR2101716	NA	missing	Susceptible	2.2.1.1
SRR2101718	NA	missing	Susceptible	2.2.1
SRR2101719	NA	missing	Susceptible	2.2.1
SRR2101724	NA	missing	Susceptible	2.2.1
SRR2101731	NA	missing	MDR	2.2.2
SRR2101733	NA	missing	Susceptible	2.2.1
SRR2101734	NA	missing	Susceptible	2.2.1
SRR2101737	NA	missing	Susceptible	2.2.1.1
SRR2101741	NA	missing	Susceptible	2.2.1
SRR2101742	NA	missing	Susceptible	2.2.1
SRR2101746	NA	missing	Susceptible	2.2.1
SRR2101750	NA	missing	Susceptible	2.2.1
SRR2101751	NA	missing	Susceptible	2.2.1
SRR2101760	NA	missing	Susceptible	2.2.1
SRR2101762	NA	missing	Susceptible	2.2.1
SRR2101771	NA	missing	Susceptible	2.2.1
SRR2101776	NA	missing	Susceptible	2.2.1
SRR2101782	NA	missing	Susceptible	2.2.1
SRR2101785	NA	missing	Susceptible	2.2.1
SRR2101792	NA	missing	Susceptible	2.2.1
SRR2101797	NA	missing	Susceptible	2.2.1
SRR2101801	NA	missing	Other	2.2.1.1
SRR2101814	NA	missing	Susceptible	2.2.1
SRR2101828	NA	missing	Susceptible	2.2.1
SRR2328057	Australia	2009	MDR	2.2.1
SRR2333215	Australia	2011	Other	2.2.2
SRR2469371	NA	missing	Susceptible	2.2.1
SRR3055243	NA	missing	Other	2.2.1
SRR3055715	South Africa	2002	Susceptible	2.2.1
SRR3055716	South Africa	2005	MDR	2.2.1
SRR3082071	Tajikistan	2010	Susceptible	2.2.1
SRR3082082	Djibouti	2014	MDR	2.2.1
SRR3082088	Djibouti	2014	Other	2.2.1
SRR3082095	Tajikistan	2009	MDR	2.2.1
SRR3082102	Tajikistan	2009	Susceptible	2.2.1
SRR3082105	Tajikistan	2010	MDR	2.2.1
SRR3082106	Tajikistan	2010	MDR	2.2.1
SRR3082108	Tajikistan	2009	MDR	2.2.1
SRR3082109	Tajikistan	2010	MDR	2.2.1
SRR3082113	Italy	2011	MDR	2.2.1
SRR3082115	Tajikistan	2010	Susceptible	2.2.1
SRR3082116	Tajikistan	2009	MDR	2.2.1

SRR3082117	Tajikistan	2009	MDR	2.2.1
SRR3082118	Tajikistan	2009	MDR	2.2.1
SRR3082121	Italy	2015	MDR	2.2.1
SRR3082123	Italy	2011	MDR	2.2.1
SRR3082127	Tajikistan	2010	Susceptible	2.2.1
SRR3082128	Tajikistan	2010	MDR	2.2.1
SRR3082130	Tajikistan	2010	XDR	2.2.1
SRR3085246	Tajikistan	2010	Susceptible	2.2.1
SRR3085247	Tajikistan	2010	MDR	2.2.1
SRR3085248	Italy	2010	MDR	2.2.1
SRR3085252	Italy	2014	MDR	2.2.1
SRR3085254	Italy	2009	MDR	2.2.1
SRR3085255	Tajikistan	2010	Susceptible	2.2.1
SRR3085256	Tajikistan	2010	MDR	2.2.1
SRR3085257	Tajikistan	2009	MDR	2.2.1
SRR3085258	Tajikistan	2010	MDR	2.2.1
SRR3085261	Tajikistan	2010	MDR	2.2.1
SRR3085263	Tajikistan	2009	Other	2.2.1
SRR3085264	Tajikistan	2009	MDR	2.2.1
SRR3085265	Tajikistan	2009	MDR	2.2.1
SRR3085268	Tajikistan	2010	MDR	2.2.1
SRR3085269	Tajikistan	2010	MDR	2.2.1
SRR3085270	Tajikistan	2010	MDR	2.2.1
SRR3085271	Tajikistan	2010	MDR	2.2.1
SRR3085273	Tajikistan	2009	Susceptible	2.2.1
SRR3085274	Tajikistan	2009	MDR	2.2.1
SRR3085275	Italy	2012	MDR	2.2.1
SRR3085276	Tajikistan	2010	Susceptible	2.2.1
SRR3085277	Tajikistan	2010	Susceptible	2.2.1
SRR3085278	Tajikistan	2009	Susceptible	2.2.1
SRR3085281	Italy	2012	MDR	2.2.1
SRR3085282	Italy	2011	MDR	2.2.1
SRR3085285	Tajikistan	Missing	MDR	2.2.1
SRR3085287	Tajikistan	Missing	MDR	2.2.1
SRR3085288	Tajikistan	Missing	MDR	2.2.1
SRR3085289	Tajikistan	2010	MDR	2.2.1
SRR3085290	Italy	2015	XDR	2.2.1
SRR3085291	Tajikistan	2009	Susceptible	2.2.1
SRR3085292	Italy	2015	XDR	2.2.1
SRR3085293	Tajikistan	2009	MDR	2.2.1
SRR3085295	Tajikistan	2010	Other	2.2.1
SRR3085296	Tajikistan	2010	MDR	2.2.1
SRR3085302	Tajikistan	2010	MDR	2.2.1
SRR3085313	Italy	2009	MDR	2.2.1
SRR3085314	Italy	2013	MDR	2.2.1
SRR3085315	Italy	2013	MDR	2.2.1
SRR3085324	Italy	2010	MDR	2.2.1
SRR3085325	Italy	2012	MDR	2.2.1
SRR3085329	Tajikistan	2010	Susceptible	2.2.1
SRR3085339	Djibouti	2014	Other	2.2.1
SRR3085347	Tajikistan	2010	MDR	2.2.1
SRR3085349	Tajikistan	2009	MDR	2.2.1
SRR3085353	Tajikistan	2009	MDR	2.2.1
SRR3085356	Italy	2015	MDR	2.2.1
SRR3085358	Italy	2011	MDR	2.2.1
SRR3085361	Tajikistan	2010	XDR	2.2.1
SRR3086350	Tajikistan	2010	Susceptible	2.2.1
SRR3086353	Tajikistan	2010	MDR	2.2.1
SRR3086356	Tajikistan	2010	MDR	2.2.1
SRR3086357	Tajikistan	2010	MDR	2.2.1
SRR3086358	Tajikistan	2009	MDR	2.2.1

SRR3086359	Italy	2012	MDR	2.2.1
SRR3086360	Tajikistan	2009	Susceptible	2.2.1
SRR3086364	Tajikistan	2010	MDR	2.2.1
SRR3086365	Italy	2015	XDR	2.2.1
SRR3086366	Tajikistan	2010	Other	2.2.1
SRR3086367	Tajikistan	2010	MDR	2.2.1
SRR3086373	Tajikistan	2010	MDR	2.2.1
SRR3086379	Italy	2013	MDR	2.2.1
SRR3086406	Italy	2010	MDR	2.2.1
SRR3086407	Italy	2012	MDR	2.2.1
SRR3105738	India	2009	Other	2.2.1
SRR3105751	India	2009	MDR	2.2.1
SRR3105778	India	2009	MDR	2.2.1.1
SRR3105786	India	2009	Other	2.2.1
SRR3105790	India	2009	MDR	2.2.1.1
SRR3130008	Israel	not applicable	Susceptible	2.2.1
SRR3136446	Israel	not applicable	Susceptible	2.2.1
SRR3205958	Thailand	09-nov-07	MDR	2.1
SRR3205959	Thailand	19-jan-08	MDR	2.1
SRR3205960	Thailand	31-Oct-2008	MDR	2.2.1
SRR3205961	Thailand	24-jul-09	MDR	2.2.1
SRR3205962	Thailand	12-Sep-2009	MDR	2.2.1
SRR3205963	Thailand	14-Oct-2011	MDR	2.2.1
SRR3205964	Thailand	15-jun-12	MDR	2.2.1
SRR3416776	China	06-nov-10	Susceptible	2.1
SRR3510602	Georgia	14-jan-15	Other	2.2.1
SRR3544716	Georgia	12-jun-14	XDR	2.2.1
SRR3544718	Georgia	2015	MDR	2.2.1
SRR3544724	Georgia	2015	MDR	2.2.1
SRR3544725	Georgia	2014	Other	2.2.1
SRR3544726	Georgia	2014	Other	2.2.1
SRR3544727	Georgia	2014	MDR	2.2.1
SRR3544728	Georgia	2013	MDR	2.2.1
SRR3544729	Georgia	2014	XDR	2.2.1
SRR3544730	Georgia	2014	Other	2.2.1
SRR3544732	Georgia	2014	MDR	2.2.1
SRR3544733	Georgia	2014	MDR	2.2.1
SRR3544734	Georgia	2013	MDR	2.2.1
SRR3544735	Georgia	2014	MDR	2.2.1
SRR3544737	Georgia	2014	Other	2.2.1
SRR3544739	Georgia	2015	MDR	2.2.1
SRR3544741	Georgia	2015	MDR	2.2.1
SRR3544743	Georgia	2014	XDR	2.2.1
SRR3544745	Georgia	09-jan-15	Susceptible	2.2.1
SRR3544746	Georgia	09-Dec-2014	Susceptible	2.2.1
SRR3544747	Georgia	2014	XDR	2.2.1
SRR3544748	Georgia	2014	MDR	2.2.1
SRR3544749	Georgia	2014	Susceptible	2.2.1
SRR3544751	Georgia	2015	MDR	2.2.1
SRR3587419	United States of America	jan-16	Susceptible	2.2.1
SRR3587420	United States of America	jan-16	Susceptible	2.2.1
SRR3587421	United States of America	jan-16	Susceptible	2.2.1
SRR3587422	United States of America	jan-16	Susceptible	2.2.1
SRR3587423	United States of America	jan-16	Susceptible	2.2.1
SRR3587425	United States of America	jan-16	Susceptible	2.2.1
SRR3587426	United States of America	jan-16	Susceptible	2.2.1
SRR3587439	United States of America	jan-16	Susceptible	2.2.1
SRR3587486	United States of America	jan-16	Susceptible	2.2.1
SRR3619841	Myanmar	Aug-2013	Other	2.2.1
SRR3675220	United Kingdom	01-jan-15	MDR	2.2.1
SRR3675225	United Kingdom	01-jan-15	Susceptible	2.2.1.2

SRR3675262	United Kingdom	01-jan-15	MDR	2.2.1
SRR3675285	United Kingdom	01-jan-15	MDR	2.2.1
SRR3675289	United Kingdom	01-jan-15	MDR	2.2.1
SRR3675290	United Kingdom	01-jan-15	Susceptible	2.2.1
SRR3675314	United Kingdom	01-jan-15	Susceptible	2.2.1
SRR3675485	United Kingdom	01-jan-15	Other	2.2.1
SRR3675512	United Kingdom	01-jan-15	Other	2.2.1
SRR3675523	United Kingdom	01-jan-15	MDR	2.2.1
SRR3732568	Peru	missing	Susceptible	2.2.1
SRR3732588	Peru	missing	MDR	2.2.1
SRR3732589	Peru	missing	MDR	2.2.1
SRR3742653	China	12-01-2010	MDR	2.2.1
SRR3742654	China	08-09-2010	MDR	2.2.1
SRR3742655	China	03-11-2009	MDR	2.2.1
SRR3742656	China	31-08-2011	MDR	2.2.1
SRR3742657	China	20-09-2012	Other	2.2.1
SRR3742658	China	09-11-2010	Other	2.2.1
SRR3742659	China	05-07-2011	XDR	2.2.1
SRR3742660	China	29-12-2011	XDR	2.2.1
SRR3742661	China	19-07-2012	XDR	2.2.1
SRR3742662	China	12-11-2012	XDR	2.2.1
SRR3742663	China	14-02-2011	MDR	2.2.1
SRR3742664	China	11-08-2011	MDR	2.2.1
SRR3742666	China	21-10-2008	Susceptible	2.2.1
SRR3742668	China	15-08-2012	XDR	2.2.1
SRR3742669	China	20-06-2007	XDR	2.2.1
SRR3742670	China	09-10-2008	MDR	2.2.1
SRR3743203	Georgia	2015	MDR	2.2.1
SRR3743368	Moldova	16-jun-15	Other	2.2.1
SRR3743371	Moldova	12-jan-15	Susceptible	2.2.1
SRR3743375	Moldova	24-Aug-2015	XDR	2.2.1
SRR3743384	Moldova	15-jun-15	MDR	2.2.1
SRR3743388	Moldova	12-May-2015	MDR	2.2.1
SRR3743392	Moldova	15-Apr-2015	MDR	2.2.1
SRR3743393	Moldova	16-mar-15	MDR	2.2.1
SRR3743397	Moldova	08-Apr-2015	MDR	2.2.1
SRR3743399	Moldova	02-Oct-2015	MDR	2.2.1
SRR3743400	Moldova	25-May-2015	MDR	2.2.1
SRR3743401	Moldova	09-nov-15	MDR	2.2.1
SRR3743404	Moldova	06-May-2015	MDR	2.2.1
SRR3743406	Moldova	30-jan-15	MDR	2.2.1
SRR3743407	Moldova	09-Sep-2015	MDR	2.2.1
SRR3743409	Moldova	14-May-2015	MDR	2.2.1
SRR3743410	Moldova	29-Dec-2015	MDR	2.2.1
SRR3743411	Moldova	05-jan-16	Susceptible	2.2.1
SRR3743414	Moldova	18-nov-15	Susceptible	2.2.1
SRR3743415	Moldova	11-Feb-2015	MDR	2.2.1
SRR3743433	Moldova	24-Aug-2015	MDR	2.2.1
SRR3743436	Moldova	03-nov-15	Susceptible	2.2.1
SRR3743438	Moldova	18-mar-15	MDR	2.2.1
SRR3743473	Moldova	27-jul-15	MDR	2.2.1
SRR3743474	Moldova	07-May-2014	MDR	2.2.1
SRR3743475	Moldova	05-Sep-2015	MDR	2.2.1
SRR3743481	Moldova	10-nov-15	MDR	2.2.1
SRR3743487	Moldova	06-May-2015	XDR	2.2.1
SRR3743488	Moldova	11-nov-15	MDR	2.2.1
SRR3743489	Moldova	24-jun-15	MDR	2.2.1
SRR3743490	Moldova	11-May-2015	MDR	2.2.1
SRR3743492	Moldova	13-jan-15	XDR	2.2.1
SRR3743494	Moldova	28-jan-15	MDR	2.2.1
SRR3743497	Moldova	13-Feb-2015	MDR	2.2.1

SRR3743499	Moldova	10-nov-14	MDR	2.2.1
SRR3743500	Moldova	12-Feb-2015	XDR	2.2.1
SRR4033089	South Africa	Oct-2012	Susceptible	2.2.1
SRR4033100	South Africa	Oct-2012	Susceptible	2.2.1
SRR4033112	South Africa	Oct-2012	Susceptible	2.2.1
SRR4033123	South Africa	Oct-2012	Susceptible	2.2.1
SRR4033134	South Africa	Oct-2012	Susceptible	2.2.1
SRR4033145	South Africa	Oct-2012	Susceptible	2.2.1
SRR4033152	South Africa	May-2013	Other	2.2.1
SRR4033153	South Africa	May-2013	Other	2.2.1
SRR4033154	South Africa	May-2013	Other	2.2.1
SRR4033155	South Africa	May-2013	Other	2.2.1
SRR4033156	South Africa	Oct-2012	Susceptible	2.2.1
SRR4033157	South Africa	May-2013	Other	2.2.1
SRR4033158	South Africa	May-2013	Other	2.2.1
SRR4033159	South Africa	May-2013	Other	2.2.1
SRR4033160	South Africa	May-2013	Other	2.2.1
SRR4033161	South Africa	May-2013	Other	2.2.1
SRR4033163	South Africa	May-2013	Other	2.2.1
SRR4033164	South Africa	May-2013	Other	2.2.1
SRR4033165	South Africa	May-2013	Other	2.2.1
SRR4033166	South Africa	May-2013	Other	2.2.1
SRR4033167	South Africa	May-2013	Other	2.2.1
SRR4033168	South Africa	Oct-2012	Susceptible	2.2.1
SRR4033169	South Africa	May-2013	Other	2.2.1
SRR4033170	South Africa	May-2013	Other	2.2.1
SRR4033171	South Africa	May-2013	Other	2.2.1
SRR4033172	South Africa	May-2013	Other	2.2.1
SRR4033173	South Africa	May-2013	Other	2.2.1
SRR4033174	South Africa	May-2013	Other	2.2.1
SRR4033175	South Africa	May-2013	Other	2.2.1
SRR4033176	South Africa	May-2013	Other	2.2.1
SRR4033177	South Africa	May-2013	Other	2.2.1
SRR4033178	South Africa	May-2013	Other	2.2.1
SRR4033179	South Africa	Oct-2012	Susceptible	2.2.1
SRR4033180	South Africa	May-2013	Other	2.2.1
SRR4033181	South Africa	May-2013	Other	2.2.1
SRR4033182	South Africa	May-2013	Other	2.2.1
SRR4033183	South Africa	May-2013	Other	2.2.1
SRR4033184	South Africa	May-2013	Other	2.2.1
SRR4033185	South Africa	May-2013	Other	2.2.1
SRR4033186	South Africa	May-2013	Other	2.2.1
SRR4033187	South Africa	May-2013	Other	2.2.1
SRR4033188	South Africa	May-2013	Other	2.2.1
SRR4033189	South Africa	May-2013	Other	2.2.1
SRR4033191	South Africa	May-2013	Other	2.2.1
SRR4033192	South Africa	May-2013	Other	2.2.1
SRR4033193	South Africa	May-2013	Other	2.2.1
SRR4033194	South Africa	May-2013	Other	2.2.1
SRR4033195	South Africa	May-2013	Other	2.2.1
SRR4033196	South Africa	May-2013	Other	2.2.1
SRR4033197	South Africa	May-2013	Other	2.2.1
SRR4033198	South Africa	May-2013	Other	2.2.1
SRR4033199	South Africa	May-2013	Other	2.2.1
SRR4033201	South Africa	May-2013	Other	2.2.1
SRR4033204	South Africa	May-2013	Other	2.2.1
SRR4033205	South Africa	May-2013	Other	2.2.1
SRR4033206	South Africa	May-2013	Other	2.2.1
SRR4033207	South Africa	May-2013	Other	2.2.1
SRR4033208	South Africa	May-2013	Other	2.2.1
SRR4033209	South Africa	May-2013	Other	2.2.1

SRR4033211	South Africa	May-2013	Other	2.2.1
SRR4033212	South Africa	May-2013	Other	2.2.1
SRR4033213	South Africa	May-2013	Other	2.2.1
SRR4033214	South Africa	Oct-2012	Susceptible	2.2.1
SRR4033225	South Africa	Oct-2012	Susceptible	2.2.1
SRR4033237	South Africa	Dec-2012	MDR	2.2.1
SRR4033248	South Africa	Dec-2012	MDR	2.2.1
SRR4033260	South Africa	Dec-2012	MDR	2.2.1
SRR4033271	South Africa	Dec-2012	MDR	2.2.1
SRR4033283	South Africa	Dec-2012	MDR	2.2.1
SRR4033294	South Africa	Dec-2012	MDR	2.2.1
SRR4033295	South Africa	May-2013	Susceptible	2.2.1
SRR4033296	South Africa	May-2013	Susceptible	2.2.1
SRR4033297	South Africa	May-2013	Susceptible	2.2.1
SRR4033298	South Africa	May-2013	Susceptible	2.2.1
SRR4033299	South Africa	May-2013	Susceptible	2.2.1
SRR4033300	South Africa	May-2013	Susceptible	2.2.1
SRR4033304	South Africa	May-2013	Susceptible	2.2.1
SRR4033305	South Africa	Dec-2012	MDR	2.2.1
SRR4033306	South Africa	May-2013	Susceptible	2.2.1
SRR4033307	South Africa	May-2013	Susceptible	2.2.1
SRR4033310	South Africa	May-2013	Susceptible	2.2.1
SRR4033311	South Africa	May-2013	Susceptible	2.2.1
SRR4033312	South Africa	May-2013	Susceptible	2.2.1
SRR4033315	South Africa	May-2013	Susceptible	2.2.1
SRR4033316	South Africa	May-2013	Susceptible	2.2.1
SRR4033318	South Africa	Dec-2012	MDR	2.2.1
SRR4033319	South Africa	May-2013	Susceptible	2.2.1
SRR4033320	South Africa	May-2013	Susceptible	2.2.1
SRR4033321	South Africa	May-2013	Susceptible	2.2.1
SRR4033322	South Africa	May-2013	Susceptible	2.2.1
SRR4033323	South Africa	May-2013	Susceptible	2.2.1
SRR4033324	South Africa	May-2013	Susceptible	2.2.1
SRR4033325	South Africa	May-2013	Susceptible	2.2.1
SRR4033326	South Africa	May-2013	Susceptible	2.2.1
SRR4033328	South Africa	May-2013	Susceptible	2.2.1
SRR4033329	South Africa	May-2013	Susceptible	2.2.1
SRR4033331	South Africa	May-2013	Susceptible	2.2.1
SRR4033332	South Africa	May-2013	Susceptible	2.2.1
SRR4033333	South Africa	May-2013	Susceptible	2.2.1
SRR4033334	South Africa	May-2013	Susceptible	2.2.1
SRR4033335	South Africa	May-2013	Susceptible	2.2.1
SRR4033336	South Africa	May-2013	Susceptible	2.2.1
SRR4033337	South Africa	May-2013	Susceptible	2.2.1
SRR4033338	South Africa	May-2013	Susceptible	2.2.1
SRR4033339	South Africa	May-2013	Susceptible	2.2.1
SRR4033340	South Africa	May-2013	Susceptible	2.2.1
SRR4033342	South Africa	May-2013	Susceptible	2.2.1
SRR4033344	South Africa	May-2013	Susceptible	2.2.1
SRR4033345	South Africa	May-2013	Susceptible	2.2.1
SRR4033346	South Africa	May-2013	Susceptible	2.2.1
SRR4033347	South Africa	May-2013	Susceptible	2.2.1
SRR4033348	South Africa	May-2013	Susceptible	2.2.1
SRR4033349	South Africa	May-2013	Susceptible	2.2.1
SRR4033350	South Africa	May-2013	Susceptible	2.2.1
SRR4033351	South Africa	May-2013	Susceptible	2.2.1
SRR4033352	South Africa	May-2013	Susceptible	2.2.1
SRR4033354	South Africa	May-2013	Susceptible	2.2.1
SRR4033355	South Africa	May-2013	Susceptible	2.2.1
SRR4033356	South Africa	May-2013	Susceptible	2.2.1
SRR4033357	South Africa	May-2013	Susceptible	2.2.1

SRR4033358	South Africa	May-2013	Susceptible	2.2.1
SRR4033359	South Africa	May-2013	Susceptible	2.2.1
SRR4033360	South Africa	May-2013	Susceptible	2.2.1
SRR4033361	South Africa	May-2013	Susceptible	2.2.1
SRR4033362	South Africa	May-2013	Susceptible	2.2.1
SRR4033366	South Africa	May-2013	Susceptible	2.2.1
SRR4033367	South Africa	May-2013	Susceptible	2.2.1
SRR4033368	South Africa	May-2013	Susceptible	2.2.1
SRR4033370	South Africa	May-2013	Susceptible	2.2.1
SRR4033371	South Africa	May-2013	Susceptible	2.2.1
SRR4033372	South Africa	May-2013	Susceptible	2.2.1
SRR4033373	South Africa	May-2013	Susceptible	2.2.1
SRR4033374	South Africa	May-2013	Susceptible	2.2.1
SRR4033375	South Africa	May-2013	Susceptible	2.2.1
SRR4033377	South Africa	May-2013	Susceptible	2.2.1
SRR4033378	South Africa	May-2013	Susceptible	2.2.1
SRR4033379	South Africa	May-2013	Susceptible	2.2.1
SRR4033381	South Africa	May-2013	Susceptible	2.2.1
SRR4033382	South Africa	May-2013	Susceptible	2.2.1
SRR4033383	South Africa	May-2013	Susceptible	2.2.1
SRR4033385	South Africa	May-2013	Susceptible	2.2.1
SRR4033386	South Africa	May-2013	Susceptible	2.2.1
SRR4033389	South Africa	May-2013	Susceptible	2.2.1
SRR4033390	South Africa	May-2013	Susceptible	2.2.1
SRR4033391	South Africa	May-2013	Susceptible	2.2.1
SRR4033392	South Africa	May-2013	Susceptible	2.2.1
SRR4033393	South Africa	May-2013	Susceptible	2.2.1
SRR4033395	South Africa	May-2013	Susceptible	2.2.1
SRR4033398	South Africa	May-2013	Susceptible	2.2.1
SRR4033404	South Africa	May-2013	Susceptible	2.2.1
SRR4033405	South Africa	May-2013	Susceptible	2.2.1
SRR4033406	South Africa	May-2013	Susceptible	2.2.1
SRR4033408	South Africa	May-2013	Susceptible	2.2.1
SRR4033479	South Africa	jan-13	Susceptible	2.2.2
SRR4033490	South Africa	jan-13	Susceptible	2.2.2
SRR4033501	South Africa	jan-13	Susceptible	2.2.2
SRR4033512	South Africa	jan-13	Susceptible	2.2.2
SRR4033534	South Africa	jan-13	Susceptible	2.2.2
SRR4033545	South Africa	jan-13	Susceptible	2.2.2
SRR4033546	South Africa	jan-13	Susceptible	2.2.2
SRR4033547	South Africa	jan-13	Susceptible	2.2.2
SRR4033548	South Africa	jan-13	Susceptible	2.2.2
SRR4033550	South Africa	jan-13	Susceptible	2.2.2
SRR4033551	South Africa	jan-13	Susceptible	2.2.2
SRR4033552	South Africa	jan-13	Susceptible	2.2.2
SRR4033588	South Africa	Oct-2012	Susceptible	2.2.1
SRR4033608	South Africa	Sep-2013	Susceptible	2.2.1
SRR4033609	South Africa	Sep-2013	Susceptible	2.2.1
SRR4033610	South Africa	Sep-2013	Susceptible	2.2.1
SRR4033611	South Africa	Sep-2013	Susceptible	2.2.1
SRR4033612	South Africa	Sep-2013	Susceptible	2.2.1
SRR4033613	South Africa	Sep-2013	Susceptible	2.2.1
SRR4033614	South Africa	Sep-2013	Susceptible	2.2.1
SRR4033615	South Africa	Sep-2013	Susceptible	2.2.1
SRR4033616	South Africa	Sep-2013	Susceptible	2.2.1
SRR4033617	South Africa	Sep-2013	Susceptible	2.2.1
SRR4033619	South Africa	Sep-2013	Susceptible	2.2.1
SRR4033620	South Africa	Sep-2013	Susceptible	2.2.1
SRR4033621	South Africa	Sep-2013	Susceptible	2.2.1
SRR4033622	South Africa	Sep-2013	Susceptible	2.2.1
SRR4033623	South Africa	Sep-2013	Susceptible	2.2.1

SRR4033717	South Africa	Sep-2013	Susceptible	2.2.1
SRR4033719	South Africa	Sep-2013	Susceptible	2.2.1
SRR4033720	South Africa	Sep-2013	Susceptible	2.2.1
SRR4033721	South Africa	Sep-2013	Susceptible	2.2.1
SRR4033723	South Africa	Sep-2013	Susceptible	2.2.1
SRR4033724	South Africa	Sep-2013	Susceptible	2.2.1
SRR4033725	South Africa	Sep-2013	Susceptible	2.2.1
SRR4033726	South Africa	Sep-2013	Susceptible	2.2.1
SRR4033727	South Africa	Sep-2013	Susceptible	2.2.1
SRR4033728	South Africa	Sep-2013	Susceptible	2.2.1
SRR4033729	South Africa	Sep-2013	Susceptible	2.2.1
SRR4033730	South Africa	Sep-2013	Susceptible	2.2.1
SRR4033731	South Africa	Sep-2013	Susceptible	2.2.1
SRR4033732	South Africa	Sep-2013	Susceptible	2.2.1
SRR4033735	South Africa	Sep-2013	Susceptible	2.2.1
SRR4033736	South Africa	Sep-2013	Susceptible	2.2.1
SRR4033737	South Africa	Sep-2013	Susceptible	2.2.1
SRR4033738	South Africa	Sep-2013	Susceptible	2.2.1
SRR4033739	South Africa	Sep-2013	Susceptible	2.2.1
SRR4033740	South Africa	Sep-2013	Susceptible	2.2.1
SRR4033741	South Africa	Sep-2013	Susceptible	2.2.1
SRR4033742	South Africa	Sep-2013	Susceptible	2.2.1
SRR4033743	South Africa	Sep-2013	Susceptible	2.2.1
SRR4033744	South Africa	Sep-2013	Susceptible	2.2.1
SRR4033746	South Africa	Sep-2013	Susceptible	2.2.1
SRR4033747	South Africa	Sep-2013	Susceptible	2.2.1
SRR4033748	South Africa	Sep-2013	Susceptible	2.2.1
SRR4033749	South Africa	Sep-2013	Susceptible	2.2.1
SRR4033751	South Africa	Sep-2013	Susceptible	2.2.1
SRR4033752	South Africa	Sep-2013	Susceptible	2.2.1
SRR4033753	South Africa	Sep-2013	Susceptible	2.2.1
SRR4033754	South Africa	Sep-2013	Susceptible	2.2.1
SRR4033755	South Africa	Sep-2013	Susceptible	2.2.1
SRR4033757	South Africa	Sep-2013	Susceptible	2.2.1
SRR4033758	South Africa	Sep-2013	Susceptible	2.2.1
SRR4033759	South Africa	Oct-2013	Susceptible	2.2.1
SRR4033760	South Africa	Oct-2013	Susceptible	2.2.1
SRR4033761	South Africa	Oct-2013	Susceptible	2.2.1
SRR4033762	South Africa	Oct-2013	Susceptible	2.2.1
SRR4033763	South Africa	Oct-2013	Susceptible	2.2.1
SRR4033764	South Africa	Oct-2013	Susceptible	2.2.1
SRR4033765	South Africa	Oct-2013	Susceptible	2.2.1
SRR4033766	South Africa	Oct-2013	Susceptible	2.2.1
SRR4033768	South Africa	Oct-2013	Susceptible	2.2.1
SRR4033769	South Africa	Oct-2013	Susceptible	2.2.1
SRR4033770	South Africa	Oct-2013	Susceptible	2.2.1
SRR4033771	South Africa	Oct-2013	Susceptible	2.2.1
SRR4033772	South Africa	Oct-2013	Susceptible	2.2.1
SRR4033773	South Africa	Oct-2013	Susceptible	2.2.1
SRR4033774	South Africa	Oct-2013	Susceptible	2.2.1
SRR4033775	South Africa	Oct-2013	Susceptible	2.2.1
SRR4033776	South Africa	Oct-2013	Susceptible	2.2.1
SRR4033777	South Africa	Oct-2013	Susceptible	2.2.1
SRR4033779	South Africa	Oct-2013	Susceptible	2.2.1
SRR4033780	South Africa	Oct-2013	Susceptible	2.2.1
SRR4033781	South Africa	Oct-2013	Susceptible	2.2.1
SRR4033783	South Africa	Oct-2013	Susceptible	2.2.1
SRR4033784	South Africa	Oct-2013	Susceptible	2.2.1
SRR4033785	South Africa	Oct-2013	Susceptible	2.2.1
SRR4033786	South Africa	Oct-2013	Susceptible	2.2.1
SRR4033787	South Africa	Oct-2013	Susceptible	2.2.1

SRR4033790	South Africa	Oct-2013	Susceptible	2.2.1
SRR4033791	South Africa	Oct-2013	Susceptible	2.2.1
SRR4033792	South Africa	Oct-2013	Susceptible	2.2.1
SRR4033793	South Africa	Oct-2013	Susceptible	2.2.1
SRR4033794	South Africa	Oct-2013	Susceptible	2.2.1
SRR4033796	South Africa	Oct-2013	Susceptible	2.2.1
SRR4033797	South Africa	Oct-2013	Susceptible	2.2.1
SRR4033798	South Africa	Oct-2013	Susceptible	2.2.1
SRR4033799	South Africa	Oct-2013	Susceptible	2.2.1
SRR4033801	South Africa	Oct-2013	Susceptible	2.2.1
SRR4033802	South Africa	Oct-2013	Susceptible	2.2.1
SRR4033803	South Africa	Oct-2013	Susceptible	2.2.1
SRR4033804	South Africa	Oct-2013	Susceptible	2.2.1
SRR4033805	South Africa	Oct-2013	Susceptible	2.2.1
SRR4033806	South Africa	Oct-2013	Susceptible	2.2.1
SRR4033807	South Africa	Oct-2013	Susceptible	2.2.1
SRR4033808	South Africa	Oct-2013	Susceptible	2.2.1
SRR4033810	South Africa	Oct-2013	Susceptible	2.2.1
SRR4033812	South Africa	Oct-2013	Susceptible	2.2.1
SRR4033813	South Africa	Oct-2013	Susceptible	2.2.1
SRR4033814	South Africa	Oct-2013	Susceptible	2.2.1
SRR4033815	South Africa	Oct-2013	Susceptible	2.2.1
SRR4033816	South Africa	Oct-2013	Susceptible	2.2.1
SRR4033817	South Africa	Oct-2013	Susceptible	2.2.1
SRR4033818	South Africa	Oct-2013	Susceptible	2.2.1
SRR4033819	South Africa	Oct-2013	Susceptible	2.2.1
SRR4033820	South Africa	Oct-2013	Susceptible	2.2.1
SRR4033821	South Africa	Oct-2013	Susceptible	2.2.1
SRR4033823	South Africa	Oct-2013	Susceptible	2.2.1
SRR4033824	South Africa	Oct-2013	Susceptible	2.2.1
SRR4033825	South Africa	Oct-2013	Susceptible	2.2.1
SRR4033826	South Africa	Oct-2013	Susceptible	2.2.1
SRR4033827	South Africa	Oct-2013	Susceptible	2.2.1
SRR4033828	South Africa	Oct-2013	Susceptible	2.2.1
SRR4033829	South Africa	Oct-2013	Susceptible	2.2.1
SRR4033830	South Africa	Oct-2013	Susceptible	2.2.1
SRR4033831	South Africa	Oct-2013	Susceptible	2.2.1
SRR4033832	South Africa	Oct-2013	Susceptible	2.2.1
SRR4033834	South Africa	Oct-2013	Susceptible	2.2.1
SRR4033835	South Africa	Oct-2013	Susceptible	2.2.1
SRR4033836	South Africa	Oct-2013	Susceptible	2.2.1
SRR4033837	South Africa	Oct-2013	Susceptible	2.2.1
SRR4033838	South Africa	Oct-2013	Susceptible	2.2.1
SRR4033839	South Africa	Oct-2013	Susceptible	2.2.1
SRR4033840	South Africa	Oct-2013	Susceptible	2.2.1
SRR4033841	South Africa	Oct-2013	Susceptible	2.2.1
SRR4033842	South Africa	Oct-2013	Susceptible	2.2.1
SRR4033843	South Africa	Oct-2013	Susceptible	2.2.1
SRR4033846	South Africa	Oct-2013	Susceptible	2.2.1
SRR4033847	South Africa	Oct-2013	Susceptible	2.2.1
SRR4033848	South Africa	Oct-2013	Susceptible	2.2.1
SRR4033849	South Africa	Oct-2013	Susceptible	2.2.1
SRR4033850	South Africa	Oct-2013	Susceptible	2.2.1
SRR4033851	South Africa	Oct-2013	Susceptible	2.2.1
SRR4033852	South Africa	Oct-2013	Susceptible	2.2.1
SRR4033853	South Africa	Oct-2013	Susceptible	2.2.1
SRR4033854	South Africa	Oct-2013	Susceptible	2.2.1
SRR4033855	South Africa	Oct-2013	Susceptible	2.2.1
SRR4033857	South Africa	Oct-2013	Susceptible	2.2.1
SRR4033858	South Africa	Oct-2013	Susceptible	2.2.1
SRR4033859	South Africa	Oct-2013	Susceptible	2.2.1

SRR4033860	South Africa	Oct-2013	Susceptible	2.2.1
SRR4033861	South Africa	Oct-2013	Susceptible	2.2.1
SRR4033864	South Africa	Oct-2013	Susceptible	2.2.1
SRR4033865	South Africa	Oct-2013	Susceptible	2.2.1
SRR4033866	South Africa	Oct-2013	Susceptible	2.2.1
SRR4033868	South Africa	Oct-2013	Susceptible	2.2.1
SRR4033869	South Africa	Oct-2013	Susceptible	2.2.1
SRR4033870	South Africa	Oct-2013	Susceptible	2.2.1
SRR4033871	South Africa	Oct-2013	Susceptible	2.2.1
SRR4033872	South Africa	Oct-2013	Susceptible	2.2.1
SRR4033873	South Africa	Oct-2013	Susceptible	2.2.1
SRR4033874	South Africa	Oct-2013	Susceptible	2.2.1
SRR4033875	South Africa	Oct-2013	Susceptible	2.2.1
SRR4033877	South Africa	Oct-2013	Susceptible	2.2.1
SRR4033878	South Africa	Sep-2013	Susceptible	2.2.1
SRR4033880	South Africa	Oct-2013	Susceptible	2.2.1
SRR4033881	South Africa	Oct-2013	Susceptible	2.2.1
SRR4033882	South Africa	Oct-2013	Susceptible	2.2.1
SRR4033883	South Africa	Oct-2013	Susceptible	2.2.1
SRR4033884	South Africa	Oct-2013	Susceptible	2.2.1
SRR4033885	South Africa	Oct-2013	Susceptible	2.2.1
SRR4033886	South Africa	Oct-2013	Susceptible	2.2.1
SRR4033889	South Africa	Sep-2013	Susceptible	2.2.1
SRR4033911	South Africa	Sep-2013	Susceptible	2.2.1
SRR4033922	South Africa	Sep-2013	Susceptible	2.2.1
SRR4033933	South Africa	Sep-2013	Susceptible	2.2.1
SRR4033944	South Africa	Sep-2013	Susceptible	2.2.1
SRR4033956	South Africa	Sep-2013	Susceptible	2.2.1
SRR4033967	South Africa	Sep-2013	Susceptible	2.2.1
SRR4033978	South Africa	Sep-2013	Susceptible	2.2.1
SRR4033989	South Africa	Sep-2013	Susceptible	2.2.1
SRR4034000	South Africa	Sep-2013	Susceptible	2.2.1
SRR4034011	South Africa	Sep-2013	Susceptible	2.2.1
SRR4034022	South Africa	Sep-2013	Susceptible	2.2.1
SRR4034033	South Africa	Sep-2013	Susceptible	2.2.1
SRR4034044	South Africa	Sep-2013	Susceptible	2.2.1
SRR4034055	South Africa	Sep-2013	Susceptible	2.2.1
SRR4034066	South Africa	Sep-2013	Susceptible	2.2.1
SRR4034067	South Africa	Sep-2013	Susceptible	2.2.1
SRR4034068	South Africa	Sep-2013	Susceptible	2.2.1
SRR4034069	South Africa	Sep-2013	Susceptible	2.2.1
SRR4034070	South Africa	Sep-2013	Susceptible	2.2.1
SRR4034071	South Africa	Sep-2013	Susceptible	2.2.1
SRR4034073	South Africa	Sep-2013	Susceptible	2.2.1
SRR4034074	South Africa	Sep-2013	Susceptible	2.2.1
SRR4034075	South Africa	Sep-2013	Susceptible	2.2.1
SRR4034077	South Africa	Sep-2013	Susceptible	2.2.1
SRR4034078	South Africa	Sep-2013	Susceptible	2.2.1
SRR4034079	South Africa	Sep-2013	Susceptible	2.2.1
SRR4034080	South Africa	Sep-2013	Susceptible	2.2.1
SRR4034081	South Africa	Sep-2013	Susceptible	2.2.1
SRR4034082	South Africa	Sep-2013	Susceptible	2.2.1
SRR4034083	South Africa	Sep-2013	Susceptible	2.2.1
SRR4034084	South Africa	Sep-2013	Susceptible	2.2.1
SRR4034085	South Africa	Sep-2013	Susceptible	2.2.1
SRR4034086	South Africa	Sep-2013	Susceptible	2.2.1
SRR4034088	South Africa	Sep-2013	Susceptible	2.2.1
SRR4034089	South Africa	Sep-2013	Susceptible	2.2.1
SRR4034090	South Africa	Sep-2013	Susceptible	2.2.1
SRR4034091	South Africa	Sep-2013	Susceptible	2.2.1
SRR4034092	South Africa	Sep-2013	Susceptible	2.2.1

SRR4034093	South Africa	Sep-2013	Susceptible	2.2.1
SRR4034094	South Africa	Sep-2013	Susceptible	2.2.1
SRR4034095	South Africa	Sep-2013	Susceptible	2.2.1
SRR4034096	South Africa	Sep-2013	Susceptible	2.2.1
SRR4034097	South Africa	Sep-2013	Susceptible	2.2.1
SRR4034099	South Africa	Sep-2013	Susceptible	2.2.1
SRR4034101	South Africa	Sep-2013	Susceptible	2.2.1
SRR4034102	South Africa	Sep-2013	Susceptible	2.2.1
SRR4034103	South Africa	Sep-2013	Susceptible	2.2.1
SRR4034104	South Africa	Sep-2013	Susceptible	2.2.1
SRR4034105	South Africa	Sep-2013	Susceptible	2.2.1
SRR4034106	South Africa	Sep-2013	Susceptible	2.2.1
SRR4034107	South Africa	Sep-2013	Susceptible	2.2.1
SRR4034108	South Africa	Sep-2013	Susceptible	2.2.1
SRR4034110	South Africa	Sep-2013	Susceptible	2.2.1
SRR4034112	South Africa	Sep-2013	Susceptible	2.2.1
SRR4034114	South Africa	Sep-2013	Susceptible	2.2.1
SRR4034115	South Africa	Sep-2013	Susceptible	2.2.1
SRR4034116	South Africa	Sep-2013	Susceptible	2.2.1
SRR4034117	South Africa	Sep-2013	Susceptible	2.2.1
SRR4034118	South Africa	Sep-2013	Susceptible	2.2.1
SRR4034119	South Africa	Sep-2013	Susceptible	2.2.1
SRR4034650	South Africa	nov-13	Susceptible	2.2.1
SRR4034651	South Africa	nov-13	Susceptible	2.2.1
SRR4034652	South Africa	nov-13	Susceptible	2.2.1
SRR4034654	South Africa	nov-13	Susceptible	2.2.1
SRR4034655	South Africa	nov-13	Susceptible	2.2.1
SRR4034656	South Africa	nov-13	Susceptible	2.2.1
SRR4034657	South Africa	nov-13	Susceptible	2.2.1
SRR4034658	South Africa	nov-13	Susceptible	2.2.1
SRR4034659	South Africa	nov-13	Susceptible	2.2.1
SRR4034661	South Africa	nov-13	Susceptible	2.2.1
SRR4034662	South Africa	nov-13	Susceptible	2.2.1
SRR4034663	South Africa	nov-13	Susceptible	2.2.1
SRR4034664	South Africa	nov-13	Susceptible	2.2.1
SRR4034665	South Africa	nov-13	Susceptible	2.2.1
SRR4034666	South Africa	nov-13	Susceptible	2.2.1
SRR4034667	South Africa	nov-13	Susceptible	2.2.1
SRR4034668	South Africa	nov-13	Susceptible	2.2.1
SRR4034669	South Africa	nov-13	Susceptible	2.2.1
SRR4034670	South Africa	nov-13	Susceptible	2.2.1
SRR4034672	South Africa	nov-13	Susceptible	2.2.1
SRR4034673	South Africa	nov-13	Susceptible	2.2.1
SRR4034674	South Africa	nov-13	Susceptible	2.2.1
SRR4034675	South Africa	nov-13	Susceptible	2.2.1
SRR4034676	South Africa	nov-13	Susceptible	2.2.1
SRR4034677	South Africa	nov-13	Susceptible	2.2.1
SRR4034678	South Africa	nov-13	Susceptible	2.2.1
SRR4034679	South Africa	nov-13	Susceptible	2.2.1
SRR4034680	South Africa	nov-13	Susceptible	2.2.1
SRR4034681	South Africa	nov-13	Susceptible	2.2.1
SRR4034683	South Africa	nov-13	Susceptible	2.2.1
SRR4034684	South Africa	nov-13	Susceptible	2.2.1
SRR4034685	South Africa	nov-13	Susceptible	2.2.1
SRR4034686	South Africa	nov-13	Susceptible	2.2.1
SRR4034687	South Africa	nov-13	Susceptible	2.2.1
SRR4034688	South Africa	nov-13	Susceptible	2.2.1
SRR4034689	South Africa	nov-13	Susceptible	2.2.1
SRR4034690	South Africa	nov-13	Susceptible	2.2.1
SRR4034691	South Africa	nov-13	Susceptible	2.2.1
SRR4034692	South Africa	nov-13	Susceptible	2.2.1

SRR4034694	South Africa	nov-13	Susceptible	2.2.1
SRR4034695	South Africa	nov-13	Susceptible	2.2.1
SRR4034696	South Africa	nov-13	Susceptible	2.2.1
SRR4034697	South Africa	nov-13	Susceptible	2.2.1
SRR4034698	South Africa	nov-13	Susceptible	2.2.1
SRR4034699	South Africa	nov-13	Susceptible	2.2.1
SRR4034700	South Africa	nov-13	Susceptible	2.2.1
SRR4034701	South Africa	nov-13	Susceptible	2.2.1
SRR4034702	South Africa	nov-13	Susceptible	2.2.1
SRR4034703	South Africa	nov-13	Susceptible	2.2.1
SRR4034705	South Africa	nov-13	Susceptible	2.2.1
SRR4034706	South Africa	nov-13	Susceptible	2.2.1
SRR4034707	South Africa	nov-13	Susceptible	2.2.1
SRR4034709	South Africa	nov-13	Susceptible	2.2.1
SRR4034710	South Africa	nov-13	Susceptible	2.2.1
SRR4034711	South Africa	nov-13	Susceptible	2.2.1
SRR4034712	South Africa	nov-13	Susceptible	2.2.1
SRR4034713	South Africa	nov-13	Susceptible	2.2.1
SRR4034714	South Africa	nov-13	Susceptible	2.2.1
SRR4034738	South Africa	Oct-2012	Susceptible	2.2.1
SRR4034739	South Africa	Oct-2012	Susceptible	2.2.1
SRR4034740	South Africa	Oct-2012	Susceptible	2.2.1
SRR4034741	South Africa	Oct-2012	Susceptible	2.2.1
SRR4034742	South Africa	Oct-2012	Susceptible	2.2.1
SRR4034743	South Africa	Oct-2012	Susceptible	2.2.1
SRR4034744	South Africa	Oct-2012	Susceptible	2.2.1
SRR4034745	South Africa	Oct-2012	Susceptible	2.2.1
SRR4034746	South Africa	Oct-2012	Susceptible	2.2.1
SRR4034809	South Africa	nov-13	Susceptible	2.2.1
SRR4034810	South Africa	nov-13	Susceptible	2.2.1
SRR4034811	South Africa	nov-13	Susceptible	2.2.1
SRR4034813	South Africa	nov-13	Susceptible	2.2.1
SRR4034814	South Africa	nov-13	Susceptible	2.2.1
SRR4034815	South Africa	nov-13	Susceptible	2.2.1
SRR4034817	South Africa	nov-13	Susceptible	2.2.1
SRR4034818	South Africa	nov-13	Susceptible	2.2.1
SRR4034820	South Africa	nov-13	Susceptible	2.2.1
SRR4034821	South Africa	nov-13	Susceptible	2.2.1
SRR4034824	South Africa	nov-13	Susceptible	2.2.1
SRR4034826	South Africa	nov-13	Susceptible	2.2.1
SRR4034828	South Africa	nov-13	Susceptible	2.2.1
SRR4034829	South Africa	nov-13	Susceptible	2.2.1
SRR4034830	South Africa	nov-13	Susceptible	2.2.1
SRR4034831	South Africa	nov-13	Susceptible	2.2.1
SRR4034833	South Africa	nov-13	Susceptible	2.2.1
SRR4034835	South Africa	nov-13	Susceptible	2.2.1
SRR4034836	South Africa	nov-13	Susceptible	2.2.1
SRR4034837	South Africa	nov-13	Susceptible	2.2.1
SRR4034838	South Africa	nov-13	Susceptible	2.2.1
SRR4034839	South Africa	nov-13	Susceptible	2.2.1
SRR4034840	South Africa	nov-13	Susceptible	2.2.1
SRR4034841	South Africa	nov-13	Susceptible	2.2.1
SRR4034842	South Africa	nov-13	Susceptible	2.2.1
SRR4034843	South Africa	nov-13	Susceptible	2.2.1
SRR4034844	South Africa	nov-13	Susceptible	2.2.1
SRR4035487	South Africa	Sep-2012	Susceptible	2.2.1
SRR4035598	South Africa	Sep-2012	Susceptible	2.2.1
SRR4035753	South Africa	Sep-2012	Susceptible	2.2.1
SRR4035864	South Africa	Sep-2012	Susceptible	2.2.1
SRR4035974	South Africa	Sep-2012	Susceptible	2.2.1
SRR4035985	South Africa	Sep-2012	Susceptible	2.2.1

SRR4037488	South Africa	jul-13	Susceptible	2.2.1
SRR4037489	South Africa	jul-13	Susceptible	2.2.1
SRR4037490	South Africa	jul-13	Susceptible	2.2.1
SRR4037491	South Africa	jul-13	Susceptible	2.2.1
SRR4037492	South Africa	jul-13	Susceptible	2.2.1
SRR4037493	South Africa	jul-13	Susceptible	2.2.1
SRR4037494	South Africa	jul-13	Susceptible	2.2.1
SRR4037495	South Africa	jul-13	Susceptible	2.2.1
SRR4037496	South Africa	jul-13	Susceptible	2.2.1
SRR4037497	South Africa	jul-13	Susceptible	2.2.1
SRR4037499	South Africa	jul-13	Susceptible	2.2.1
SRR4037500	South Africa	jul-13	Susceptible	2.2.1
SRR4037501	South Africa	jul-13	Susceptible	2.2.1
SRR4037502	South Africa	jul-13	Susceptible	2.2.1
SRR4037503	South Africa	jul-13	Susceptible	2.2.1
SRR4037504	South Africa	jul-13	Susceptible	2.2.1
SRR4037505	South Africa	jul-13	Susceptible	2.2.1
SRR4037506	South Africa	jul-13	Susceptible	2.2.1
SRR4037507	South Africa	jul-13	Susceptible	2.2.1
SRR4037508	South Africa	jul-13	Susceptible	2.2.1
SRR4037510	South Africa	jul-13	Susceptible	2.2.1
SRR4037511	South Africa	jul-13	Susceptible	2.2.1
SRR4037512	South Africa	jul-13	Susceptible	2.2.1
SRR4037513	South Africa	jul-13	Susceptible	2.2.1
SRR4037514	South Africa	jul-13	Susceptible	2.2.1
SRR4037515	South Africa	jul-13	Susceptible	2.2.1
SRR4037516	South Africa	jul-13	Susceptible	2.2.1
SRR4037517	South Africa	jul-13	Susceptible	2.2.1
SRR4037518	South Africa	jul-13	Susceptible	2.2.1
SRR4037642	South Africa	jul-13	Susceptible	2.2.1
SRR4037653	South Africa	jul-13	Susceptible	2.2.1
SRR4037675	South Africa	jul-13	Susceptible	2.2.1
SRR4037697	South Africa	jul-13	Susceptible	2.2.1
SRR4037709	South Africa	jul-13	Susceptible	2.2.1
SRR4037720	South Africa	jul-13	Susceptible	2.2.1
SRR4037731	South Africa	jul-13	Susceptible	2.2.1
SRR4037742	South Africa	jul-13	Susceptible	2.2.1
SRR4037753	South Africa	jul-13	Susceptible	2.2.1
SRR4037764	South Africa	jul-13	Susceptible	2.2.1
SRR4037766	South Africa	Aug-2013	Susceptible	2.2.1
SRR4037768	South Africa	Aug-2013	Susceptible	2.2.1
SRR4037769	South Africa	Aug-2013	Susceptible	2.2.1
SRR4037770	South Africa	Aug-2013	Susceptible	2.2.1
SRR4037771	South Africa	Aug-2013	Susceptible	2.2.1
SRR4037772	South Africa	Aug-2013	Susceptible	2.2.1
SRR4037773	South Africa	Aug-2013	Susceptible	2.2.1
SRR4037774	South Africa	Aug-2013	Susceptible	2.2.1
SRR4037775	South Africa	jul-13	Susceptible	2.2.1
SRR4037776	South Africa	Aug-2013	Susceptible	2.2.1
SRR4037778	South Africa	Aug-2013	Susceptible	2.2.1
SRR4037779	South Africa	Aug-2013	Susceptible	2.2.1
SRR4037780	South Africa	Aug-2013	Susceptible	2.2.1
SRR4037781	South Africa	Aug-2013	Susceptible	2.2.1
SRR4037782	South Africa	Aug-2013	Susceptible	2.2.1
SRR4037783	South Africa	Aug-2013	Susceptible	2.2.1
SRR4037784	South Africa	Aug-2013	Susceptible	2.2.1
SRR4037785	South Africa	Aug-2013	Susceptible	2.2.1
SRR4037786	South Africa	jul-13	Susceptible	2.2.1
SRR4037787	South Africa	Aug-2013	Susceptible	2.2.1
SRR4037788	South Africa	Aug-2013	Susceptible	2.2.1
SRR4037789	South Africa	Aug-2013	Susceptible	2.2.1

SRR4037790	South Africa	Aug-2013	Susceptible	2.2.1
SRR4037791	South Africa	Aug-2013	Susceptible	2.2.1
SRR4037792	South Africa	Aug-2013	Susceptible	2.2.1
SRR4037793	South Africa	Aug-2013	Susceptible	2.2.1
SRR4037794	South Africa	Aug-2013	Susceptible	2.2.1
SRR4037795	South Africa	Aug-2013	Susceptible	2.2.1
SRR4037796	South Africa	Aug-2013	Susceptible	2.2.1
SRR4037797	South Africa	jul-13	Susceptible	2.2.1
SRR4037798	South Africa	Aug-2013	Susceptible	2.2.1
SRR4037800	South Africa	Aug-2013	Susceptible	2.2.1
SRR4037801	South Africa	Aug-2013	Susceptible	2.2.1
SRR4037802	South Africa	Aug-2013	Susceptible	2.2.1
SRR4037803	South Africa	Aug-2013	Susceptible	2.2.1
SRR4037804	South Africa	Aug-2013	Susceptible	2.2.1
SRR4037805	South Africa	Aug-2013	Susceptible	2.2.1
SRR4037806	South Africa	Aug-2013	Susceptible	2.2.1
SRR4037807	South Africa	Aug-2013	Susceptible	2.2.1
SRR4037808	South Africa	jul-13	Susceptible	2.2.1
SRR4037809	South Africa	Aug-2013	Susceptible	2.2.1
SRR4037811	South Africa	Aug-2013	Susceptible	2.2.1
SRR4037812	South Africa	Aug-2013	Susceptible	2.2.1
SRR4037813	South Africa	Aug-2013	Susceptible	2.2.1
SRR4037814	South Africa	Aug-2013	Susceptible	2.2.1
SRR4037816	South Africa	Aug-2013	Susceptible	2.2.1
SRR4037817	South Africa	Aug-2013	Susceptible	2.2.1
SRR4037818	South Africa	Aug-2013	Susceptible	2.2.1
SRR4037820	South Africa	jul-13	Susceptible	2.2.1
SRR4037831	South Africa	jul-13	Susceptible	2.2.1
SRR4037842	South Africa	jul-13	Susceptible	2.2.1
SRR4037853	South Africa	jul-13	Susceptible	2.2.1
SRR4037864	South Africa	jul-13	Susceptible	2.2.1
SRR4037875	South Africa	jul-13	Susceptible	2.2.1
SRR4037886	South Africa	jul-13	Susceptible	2.2.1
SRR4037897	South Africa	jul-13	Susceptible	2.2.1
SRR4037908	South Africa	jul-13	Susceptible	2.2.1
SRR4037919	South Africa	jul-13	Susceptible	2.2.1
SRR4037930	South Africa	jul-13	Susceptible	2.2.1
SRR4037931	South Africa	jul-13	Susceptible	2.2.1
SRR4037933	South Africa	jul-13	Susceptible	2.2.1
SRR4037934	South Africa	jul-13	Susceptible	2.2.1
SRR4037935	South Africa	jul-13	Susceptible	2.2.1
SRR4037936	South Africa	jul-13	Susceptible	2.2.1
SRR4037937	South Africa	jul-13	Susceptible	2.2.1
SRR4037938	South Africa	jul-13	Susceptible	2.2.1
SRR4037939	South Africa	jul-13	Susceptible	2.2.1
SRR4037941	South Africa	jul-13	Susceptible	2.2.1
SRR4037942	South Africa	jul-13	Susceptible	2.2.1
SRR4037943	South Africa	jul-13	Susceptible	2.2.1
SRR4037944	South Africa	jul-13	Susceptible	2.2.1
SRR4037946	South Africa	jul-13	Susceptible	2.2.1
SRR4037947	South Africa	jul-13	Susceptible	2.2.1
SRR4037948	South Africa	jul-13	Susceptible	2.2.1
SRR4037949	South Africa	jul-13	Susceptible	2.2.1
SRR4037950	South Africa	jul-13	Susceptible	2.2.1
SRR4037953	South Africa	jul-13	Susceptible	2.2.1
SRR4037954	South Africa	jul-13	Susceptible	2.2.1
SRR4037956	South Africa	jul-13	Susceptible	2.2.1
SRR4037957	South Africa	jul-13	Susceptible	2.2.1
SRR4037958	South Africa	jul-13	Susceptible	2.2.1
SRR4037960	South Africa	jul-13	Susceptible	2.2.1
SRR4037961	South Africa	jul-13	Susceptible	2.2.1

SRR4037962	South Africa	jul-13	Susceptible	2.2.1
SRR4037964	South Africa	jul-13	Susceptible	2.2.1
SRR4037965	South Africa	jul-13	Susceptible	2.2.1
SRR4037966	South Africa	jul-13	Susceptible	2.2.1
SRR4037967	South Africa	jul-13	Susceptible	2.2.1
SRR4037968	South Africa	jul-13	Susceptible	2.2.1
SRR4037969	South Africa	jul-13	Susceptible	2.2.1
SRR4037970	South Africa	jul-13	Susceptible	2.2.1
SRR4037971	South Africa	jul-13	Susceptible	2.2.1
SRR4037973	South Africa	jul-13	Susceptible	2.2.1
SRR4037975	South Africa	jul-13	Susceptible	2.2.1
SRR4037976	South Africa	jul-13	Susceptible	2.2.1
SRR4037977	South Africa	jul-13	Susceptible	2.2.1
SRR4037978	South Africa	jul-13	Susceptible	2.2.1
SRR4037979	South Africa	jul-13	Susceptible	2.2.1
SRR4037980	South Africa	jul-13	Susceptible	2.2.1
SRR4037981	South Africa	jul-13	Susceptible	2.2.1
SRR4037982	South Africa	jul-13	Susceptible	2.2.1
SRR4037983	South Africa	jul-13	Susceptible	2.2.1
SRR4037984	South Africa	jul-13	Susceptible	2.2.1
SRR413213	NA	NA	Other	2.2.1
SRR413216	NA	NA	Other	2.2.1
SRR413221	NA	NA	Other	2.2.1
SRR413226	NA	NA	Other	2.2.1
SRR413228	NA	NA	MDR	2.2.1
SRR413231	NA	NA	MDR	2.2.1
SRR413266	NA	NA	MDR	2.2.1
SRR4423135	Bangladesh	13-11-2007	MDR	2.2.2
SRR4423139	Bangladesh	21-06-2008	MDR	2.2.2
SRR4423141	Bangladesh	10-09-2008	MDR	2.2.1
SRR4423146	Bangladesh	09-04-2011	MDR	2.2.2
SRR4423148	Bangladesh	11-03-2012	MDR	2.2.2
SRR4423151	Bangladesh	08-09-2012	MDR	2.2.1
SRR4423153	Bangladesh	11-05-2011	MDR	2.2.2
SRR4423154	Bangladesh	22-05-2007	MDR	2.2.1
SRR4423156	Bangladesh	07-05-2007	MDR	2.2.1
SRR4423159	Bangladesh	26-11-2012	MDR	2.2.1
SRR4423161	Bangladesh	22-09-2012	MDR	2.2.1
SRR4423162	Bangladesh	01-08-2007	MDR	2.2.1
SRR4423170	Bangladesh	15-01-2009	MDR	2.2.1
SRR4423171	Bangladesh	13-02-2012	MDR	2.2.1
SRR4423173	Bangladesh	11-11-2009	MDR	2.2.1
SRR4423174	Bangladesh	23-02-2010	MDR	2.2.2
SRR4423180	Bangladesh	22-09-2010	MDR	2.2.1
SRR475257	NA	NA	Other	2.2.1
SRR5007156	United Kingdom	07-nov-14	Susceptible	2.2.1
SRR5007162	United Kingdom	04-nov-13	Susceptible	2.2.1
SRR5007164	United Kingdom	22-Dec-2013	Susceptible	2.2.1
SRR5007166	United Kingdom	07-nov-14	Susceptible	2.2.1
SRR5007171	United Kingdom	09-nov-14	Susceptible	2.2.1
SRR5007173	United Kingdom	10-nov-13	Susceptible	2.2.1
SRR5007181	United Kingdom	04-nov-13	Susceptible	2.2.1
SRR5007202	United Kingdom	09-nov-14	Susceptible	2.2.1
SRR5007203	United Kingdom	06-nov-14	Susceptible	2.2.1
SRR5007205	United Kingdom	06-nov-14	Susceptible	2.2.1
SRR5065200	Vietnam	2010	Susceptible	2.2.1
SRR5065204	Vietnam	2011	Susceptible	2.2.1
SRR5065208	Vietnam	2011	Susceptible	2.2.1
SRR5065209	Vietnam	2011	Other	2.2.1
SRR5065210	Vietnam	2011	Susceptible	2.2.1
SRR5065212	Vietnam	2010	Susceptible	2.2.1

SRR5065213	Vietnam	2010	Other	2.2.1
SRR5065215	Vietnam	2010	Other	2.2.1
SRR5065216	Vietnam	2011	Susceptible	2.2.1
SRR5065217	Vietnam	2011	Other	2.2.1
SRR5065218	Vietnam	2010	Other	2.2.1
SRR5065219	Vietnam	2011	Other	2.2.1
SRR5065220	Vietnam	2011	Susceptible	2.2.1
SRR5065221	Vietnam	2011	Susceptible	2.2.1
SRR5065222	Vietnam	2011	Other	2.2.1.1
SRR5065224	Vietnam	2011	Susceptible	2.2.1
SRR5065226	Vietnam	2011	Susceptible	2.2.1
SRR5065228	Vietnam	2011	MDR	2.2.1
SRR5065229	Vietnam	2011	Susceptible	2.2.1
SRR5065231	Vietnam	2010	Other	2.2.1.1
SRR5065232	Vietnam	2011	Susceptible	2.2.1
SRR5065234	Vietnam	2011	Other	2.2.1.1
SRR5065235	Vietnam	2010	Other	2.2.1
SRR5065238	Vietnam	2010	Other	2.2.1
SRR5065240	Vietnam	2011	Susceptible	2.2.1
SRR5065241	Vietnam	2010	Other	2.2.1
SRR5065245	Vietnam	2010	Other	2.2.1.1
SRR5065249	Vietnam	2011	Susceptible	2.2.1
SRR5065250	Vietnam	2011	MDR	2.2.1
SRR5065251	Vietnam	2010	Susceptible	2.2.1
SRR5065253	Vietnam	2011	Other	2.2.1
SRR5065255	Vietnam	2010	Susceptible	2.2.1
SRR5065257	Vietnam	2011	Other	2.2.1
SRR5065259	Vietnam	2010	Other	2.2.1
SRR5065260	Vietnam	2010	Other	2.2.1
SRR5065261	Vietnam	2011	MDR	2.2.1
SRR5065262	Vietnam	2011	Susceptible	2.2.1
SRR5065265	Vietnam	2010	Other	2.2.1.1
SRR5065269	Vietnam	2010	Other	2.2.1
SRR5065270	Vietnam	2010	Other	2.2.1
SRR5065272	Vietnam	2010	Susceptible	2.2.1
SRR5065273	Vietnam	2010	Susceptible	2.2.1
SRR5065274	Vietnam	2011	Other	2.2.1.1
SRR5065275	Vietnam	2011	Susceptible	2.2.1
SRR5065277	Vietnam	2010	Other	2.2.1
SRR5065278	Vietnam	2010	Susceptible	2.1
SRR5065281	Vietnam	2011	Other	2.2.1
SRR5065282	Vietnam	2011	Other	2.2.1
SRR5065283	Vietnam	2010	Susceptible	2.1
SRR5065286	Vietnam	2011	Susceptible	2.2.1
SRR5065288	Vietnam	2011	Other	2.2.1
SRR5065290	Vietnam	2011	Susceptible	2.2.2
SRR5065291	Vietnam	2011	Other	2.2.1
SRR5065292	Vietnam	2011	Other	2.1
SRR5065293	Vietnam	2010	Other	2.2.1
SRR5065294	Vietnam	2011	Susceptible	2.1
SRR5065297	Vietnam	2011	Susceptible	2.1
SRR5065298	Vietnam	2010	Susceptible	2.2.1
SRR5065299	Vietnam	2011	Other	2.2.1.1
SRR5065300	Vietnam	2011	Susceptible	2.2.1.1
SRR5065301	Vietnam	2010	Susceptible	2.1
SRR5065303	Vietnam	2011	Susceptible	2.2.1
SRR5065304	Vietnam	2011	Susceptible	2.2.1
SRR5065307	Vietnam	2010	Susceptible	2.2.1
SRR5065308	Vietnam	2011	Susceptible	2.1
SRR5065311	Vietnam	2011	Susceptible	2.2.1.1
SRR5065312	Vietnam	2011	Susceptible	2.2.1

SRR5065313	Vietnam	2011	Other	2.2.1
SRR5065314	Vietnam	2010	Other	2.2.1
SRR5065315	Vietnam	2011	Susceptible	2.2.1
SRR5065319	Vietnam	2011	Susceptible	2.2.1
SRR5065321	Vietnam	2011	Susceptible	2.2.1
SRR5065324	Vietnam	2010	Susceptible	2.2.1
SRR5065325	Vietnam	2010	Other	2.2.1.1
SRR5065326	Vietnam	2011	Other	2.2.1
SRR5065327	Vietnam	2010	Susceptible	2.2.1
SRR5065328	Vietnam	2011	Other	2.2.2
SRR5065332	Vietnam	2011	Susceptible	2.2.1
SRR5065333	Vietnam	2010	Susceptible	2.2.1
SRR5065334	Vietnam	2011	Susceptible	2.2.1
SRR5065336	Vietnam	2011	MDR	2.2.1
SRR5065338	Vietnam	2010	Susceptible	2.2.1
SRR5065339	Vietnam	2010	MDR	2.2.1
SRR5065340	Vietnam	2011	Susceptible	2.2.1
SRR5065341	Vietnam	2010	Susceptible	2.2.1
SRR5065343	Vietnam	2010	Susceptible	2.2.1
SRR5065344	Vietnam	2011	Susceptible	2.2.1
SRR5065345	Vietnam	2011	Susceptible	2.2.1
SRR5065347	Vietnam	2011	Susceptible	2.2.1
SRR5065350	Vietnam	2011	Susceptible	2.2.1
SRR5065351	Vietnam	2011	Susceptible	2.2.1
SRR5065352	Vietnam	2010	Susceptible	2.2.1
SRR5065353	Vietnam	2010	Susceptible	2.2.1
SRR5065354	Vietnam	2010	Susceptible	2.2.1
SRR5065356	Vietnam	2011	Other	2.2.1
SRR5065357	Vietnam	2010	Other	2.2.1
SRR5065358	Vietnam	2011	Other	2.2.1.1
SRR5065359	Vietnam	2010	Susceptible	2.2.1
SRR5065360	Vietnam	2010	Susceptible	2.2.1
SRR5065361	Vietnam	2011	Other	2.2.1
SRR5065362	Vietnam	2011	Other	2.2.1
SRR5065364	Vietnam	2010	Susceptible	2.2.1
SRR5065365	Vietnam	2011	Susceptible	2.2.1
SRR5065366	Vietnam	2010	Susceptible	2.2.1
SRR5065367	Vietnam	2011	Susceptible	2.2.1
SRR5065368	Vietnam	2011	Susceptible	2.1
SRR5065370	Vietnam	2010	Other	2.2.1
SRR5065371	Vietnam	2010	MDR	2.2.2
SRR5065372	Vietnam	2011	Susceptible	2.2.1
SRR5065373	Vietnam	2011	Susceptible	2.2.1
SRR5065376	Vietnam	2010	Other	2.2.1
SRR5065377	Vietnam	2011	Other	2.2.1
SRR5065378	Vietnam	2010	Susceptible	2.2.1
SRR5065380	Vietnam	2010	Other	2.2.1
SRR5065381	Vietnam	2010	MDR	2.2.1.1
SRR5065382	Vietnam	2011	Susceptible	2.1
SRR5065384	Vietnam	2010	Susceptible	2.2.1
SRR5065385	Vietnam	2010	Other	2.2.1
SRR5065387	Vietnam	2011	Susceptible	2.2.1
SRR5065388	Vietnam	2010	Susceptible	2.2.1.1
SRR5065397	Vietnam	2010	Susceptible	2.2.1
SRR5065400	Vietnam	2010	Other	2.2.1
SRR5065401	Vietnam	2011	Susceptible	2.2.1
SRR5065403	Vietnam	2010	Other	2.2.1
SRR5065404	Vietnam	2010	Other	2.2.1
SRR5065405	Vietnam	2011	Other	2.2.1
SRR5065406	Vietnam	2011	Other	2.2.1.1
SRR5065407	Vietnam	2011	MDR	2.2.1

SRR5065409	Vietnam	2010	Other	2.2.1
SRR5065410	Vietnam	2010	Other	2.2.1
SRR5065411	Vietnam	2010	Other	2.1
SRR5065412	Vietnam	2010	Other	2.2.1
SRR5065413	Vietnam	2011	Other	2.2.1
SRR5065414	Vietnam	2010	Susceptible	2.2.1
SRR5065418	Vietnam	2010	Susceptible	2.1
SRR5065419	Vietnam	2011	Susceptible	2.2.2
SRR5065420	Vietnam	2010	Susceptible	2.2.1.1
SRR5065421	Vietnam	2010	Susceptible	2.2.1
SRR5065422	Vietnam	2011	Other	2.2.1
SRR5065423	Vietnam	2011	Other	2.2.1
SRR5065424	Vietnam	2011	Susceptible	2.2.1
SRR5065425	Vietnam	2011	Other	2.2.1
SRR5065426	Vietnam	2011	Susceptible	2.2.1
SRR5065427	Vietnam	2011	Susceptible	2.2.1
SRR5065429	Vietnam	2011	Susceptible	2.2.1
SRR5065433	Vietnam	2011	Susceptible	2.2.1
SRR5065434	Vietnam	2011	Susceptible	2.2.1
SRR5065435	Vietnam	2011	Susceptible	2.2.1
SRR5065436	Vietnam	2011	Other	2.2.1
SRR5065438	Vietnam	2010	Susceptible	2.2.1
SRR5065440	Vietnam	2011	Other	2.2.1.2
SRR5065441	Vietnam	2010	Susceptible	2.1
SRR5065442	Vietnam	2010	Susceptible	2.2.1
SRR5065443	Vietnam	2011	Susceptible	2.2.1
SRR5065444	Vietnam	2010	Susceptible	2.2.1
SRR5065450	Vietnam	2011	Susceptible	2.2.1.1
SRR5065451	Vietnam	2011	Other	2.2.1
SRR5065457	Vietnam	2011	Other	2.2.1
SRR5065460	Vietnam	2011	Other	2.2.1
SRR5065461	Vietnam	2010	Susceptible	2.2.1
SRR5065462	Vietnam	2010	Other	2.2.1.1
SRR5065465	Vietnam	2011	Susceptible	2.2.1
SRR5065466	Vietnam	2010	Susceptible	2.2.1
SRR5065467	Vietnam	2011	Susceptible	2.1
SRR5065469	Vietnam	2010	Other	2.2.1.1
SRR5065470	Vietnam	2010	Other	2.2.1
SRR5065471	Vietnam	2010	Susceptible	2.2.1
SRR5065472	Vietnam	2011	Susceptible	2.2.1
SRR5065473	Vietnam	2010	Other	2.2.1
SRR5065474	Vietnam	2010	Other	2.2.1.1
SRR5065475	Vietnam	2010	Susceptible	2.2.1
SRR5065477	Vietnam	2011	Other	2.2.1
SRR5065478	Vietnam	2011	Other	2.2.1
SRR5065479	Vietnam	2010	Susceptible	2.2.1
SRR5065480	Vietnam	2011	MDR	2.2.1
SRR5065481	Vietnam	2011	Other	2.2.1
SRR5065482	Vietnam	2011	Other	2.2.1.1
SRR5065483	Vietnam	2011	Susceptible	2.2.1
SRR5065484	Vietnam	2010	Other	2.2.1.1
SRR5065486	Vietnam	2011	Other	2.2.1.1
SRR5065487	Vietnam	2011	Susceptible	2.1
SRR5065488	Vietnam	2010	MDR	2.2.1.1
SRR5065489	Vietnam	2010	Susceptible	2.2.1
SRR5065497	Vietnam	2011	Other	2.2.1
SRR5065499	Vietnam	2011	Susceptible	2.2.2
SRR5065500	Vietnam	2010	Other	2.2.2
SRR5065501	Vietnam	2011	Other	2.2.1
SRR5065505	Vietnam	2010	Susceptible	2.2.1
SRR5065507	Vietnam	2011	Susceptible	2.2.1.1

SRR5065510	Vietnam	2011	Susceptible	2.2.1
SRR5065511	Vietnam	2010	Other	2.2.1
SRR5065512	Vietnam	2010	Susceptible	2.2.1
SRR5065514	Vietnam	2010	Susceptible	2.2.1
SRR5065515	Vietnam	2010	Susceptible	2.2.1
SRR5065518	Vietnam	2010	Susceptible	2.2.1
SRR5065519	Vietnam	2011	Other	2.2.1
SRR5065520	Vietnam	2010	Other	2.2.1
SRR5065521	Vietnam	2011	Susceptible	2.2.1
SRR5065522	Vietnam	2011	Other	2.2.1
SRR5065524	Vietnam	2010	MDR	2.2.1
SRR5065526	Vietnam	2011	Other	2.2.1
SRR5065527	Vietnam	2011	Susceptible	2.2.1
SRR5065528	Vietnam	2011	Susceptible	2.2.1
SRR5065529	Vietnam	2011	Susceptible	2.2.1
SRR5065530	Vietnam	2010	Susceptible	2.2.1
SRR5065531	Vietnam	2011	Susceptible	2.2.1
SRR5065533	Vietnam	2011	Susceptible	2.2.1
SRR5065534	Vietnam	2010	Other	2.2.1
SRR5065535	Vietnam	2010	Other	2.2.1
SRR5065536	Vietnam	2011	Other	2.2.1.1
SRR5065537	Vietnam	2011	MDR	2.2.1
SRR5065538	Vietnam	2011	MDR	2.2.1
SRR5065541	Vietnam	2011	Other	2.2.1
SRR5065543	Vietnam	2010	Other	2.2.1.1
SRR5065544	Vietnam	2011	Susceptible	2.2.1
SRR5065545	Vietnam	2011	Susceptible	2.2.1
SRR5065547	Vietnam	2011	Susceptible	2.2.1.1
SRR5065549	Vietnam	2010	Susceptible	2.2.1.1
SRR5065550	Vietnam	2011	Other	2.2.1
SRR5065551	Vietnam	2010	Susceptible	2.1
SRR5065554	Vietnam	2011	Other	2.2.1
SRR5065555	Vietnam	2011	Other	2.2.1
SRR5065556	Vietnam	2010	Susceptible	2.2.1
SRR5065557	Vietnam	2011	Susceptible	2.2.1
SRR5065558	Vietnam	2010	Susceptible	2.2.1
SRR5065560	Vietnam	2011	MDR	2.1
SRR5065561	Vietnam	2010	Susceptible	2.2.1
SRR5065562	Vietnam	2010	Other	2.2.1
SRR5065563	Vietnam	2010	Susceptible	2.1
SRR5065565	Vietnam	2010	Other	2.2.1
SRR5065566	Vietnam	2011	Other	2.2.1
SRR5065567	Vietnam	2010	Other	2.2.2
SRR5065568	Vietnam	2011	Susceptible	2.2.1
SRR5065569	Vietnam	2011	Other	2.2.1
SRR5065570	Vietnam	2010	Susceptible	2.2.1
SRR5065572	Vietnam	2011	Susceptible	2.2.1.1
SRR5065573	Vietnam	2010	Other	2.2.1
SRR5065574	Vietnam	2011	Other	2.2.1
SRR5065575	Vietnam	2010	Susceptible	2.2.1
SRR5065576	Vietnam	2011	Other	2.2.1
SRR5065579	Vietnam	2011	Susceptible	2.1
SRR5065580	Vietnam	2010	Susceptible	2.2.2
SRR5065581	Vietnam	2011	Other	2.2.1
SRR5065582	Vietnam	2010	Other	2.2.1
SRR5065583	Vietnam	2011	Susceptible	2.2.1
SRR5065585	Vietnam	2011	Other	2.2.1
SRR5065586	Vietnam	2011	Susceptible	2.2.1.1
SRR5065588	Vietnam	2010	MDR	2.2.1
SRR5065589	Vietnam	2011	Susceptible	2.2.1.1
SRR5065590	Vietnam	2011	Susceptible	2.2.1

SRR5065591	Vietnam	2011	Susceptible	2.2.1.1
SRR5065592	Vietnam	2010	Other	2.2.1.1
SRR5065594	Vietnam	2010	Susceptible	2.2.1.1
SRR5065595	Vietnam	2011	MDR	2.2.1
SRR5065596	Vietnam	2011	MDR	2.2.1
SRR5065598	Vietnam	2011	Susceptible	2.2.1
SRR5065599	Vietnam	2010	Other	2.2.1
SRR5065600	Vietnam	2010	Susceptible	2.2.1
SRR5065601	Vietnam	2011	Susceptible	2.2.1
SRR5065604	Vietnam	2011	Susceptible	2.1
SRR5065605	Vietnam	2011	Susceptible	2.2.1
SRR5065606	Vietnam	2011	Other	2.2.1.1
SRR5065608	Vietnam	2010	MDR	2.2.1.1
SRR5065610	Vietnam	2010	Other	2.2.1
SRR5065611	Vietnam	2011	Other	2.2.1
SRR5065612	Vietnam	2010	Susceptible	2.2.1
SRR5065613	Vietnam	2010	Susceptible	2.2.1
SRR5065615	Vietnam	2011	Susceptible	2.2.1
SRR5065616	Vietnam	2011	Other	2.1
SRR5065617	Vietnam	2010	Susceptible	2.1
SRR5065618	Vietnam	2010	Other	2.2.1
SRR5065619	Vietnam	2010	Other	2.1
SRR5065620	Vietnam	2011	Susceptible	2.2.1
SRR5065621	Vietnam	2011	Other	2.2.1
SRR5065623	Vietnam	2010	Susceptible	2.2.1
SRR5065624	Vietnam	2010	Other	2.2.1
SRR5065625	Vietnam	2011	Other	2.2.1.1
SRR5065627	Vietnam	2010	MDR	2.2.1
SRR5065628	Vietnam	2011	Susceptible	2.2.1
SRR5065630	Vietnam	2011	Other	2.2.1
SRR5065631	Vietnam	2011	Other	2.2.1
SRR5065634	Vietnam	2011	Susceptible	2.2.1
SRR5065637	Vietnam	2011	Other	2.2.1
SRR5065638	Vietnam	2011	Other	2.2.1.1
SRR5065642	Vietnam	2011	Other	2.2.1
SRR5065643	Vietnam	2011	Other	2.2.1
SRR5065644	Vietnam	2010	Susceptible	2.2
SRR5065645	Vietnam	2011	Other	2.2.1
SRR5065646	Vietnam	2010	Susceptible	2.2.1
SRR5065647	Vietnam	2011	Susceptible	2.2.1
SRR5065650	Vietnam	2010	Susceptible	2.2.1
SRR5065652	Vietnam	2010	Susceptible	2.2.1
SRR5065653	Vietnam	2010	Susceptible	2.2.1.1
SRR5065654	Vietnam	2011	MDR	2.2.1.1
SRR5065656	Vietnam	2010	Susceptible	2.2.2
SRR5065658	Vietnam	2011	Susceptible	2.2.1
SRR5065660	Vietnam	2010	Susceptible	2.2.1.1
SRR5065661	Vietnam	2011	Susceptible	2.2.1
SRR5065662	Vietnam	2011	Susceptible	2.2.1
SRR5065664	Vietnam	2011	Susceptible	2.2.1
SRR5065665	Vietnam	2010	Other	2.2.1.1
SRR5065667	Vietnam	2011	Other	2.2.1
SRR5065669	Vietnam	2010	Susceptible	2.2.1
SRR5065670	Vietnam	2010	Other	2.2.1
SRR5065671	Vietnam	2010	Susceptible	2.2.1
SRR5065672	Vietnam	2011	Susceptible	2.2.1
SRR5065674	Vietnam	2011	Susceptible	2.2.1
SRR5065675	Vietnam	2010	Susceptible	2.2.1
SRR5065676	Vietnam	2011	Other	2.2.1
SRR5065678	Vietnam	2011	Susceptible	2.1
SRR5065680	Vietnam	2011	Other	2.2.1.1

SRR5065681	Vietnam	2010	Susceptible	2.2.1
SRR5065682	Vietnam	2011	Susceptible	2.2.1
SRR5065683	Vietnam	2010	Susceptible	2.2.1
SRR5065685	Vietnam	2011	Susceptible	2.2.1
SRR5065686	Vietnam	2011	Other	2.2.1
SRR5065687	Vietnam	2011	Other	2.2.1.1
SRR5065688	Vietnam	2011	Susceptible	2.2.1
SRR5065691	Vietnam	2011	Susceptible	2.2.1
SRR5065692	Vietnam	2010	Susceptible	2.2.1
SRR5065694	Vietnam	2010	Susceptible	2.2.1
SRR5065696	Vietnam	2010	Susceptible	2.2.1.1
SRR5065697	Vietnam	2011	Susceptible	2.2.1
SRR5065699	Vietnam	2011	Other	2.2.1
SRR5065700	Vietnam	2010	Other	2.2.1
SRR5065702	Vietnam	2010	Susceptible	2.2.1
SRR5065703	Vietnam	2011	Susceptible	2.2.1
SRR5065705	Vietnam	2011	Other	2.2.1
SRR5067220	Vietnam	2010	Susceptible	2.2.1
SRR5067221	Vietnam	2010	Other	2.2.1
SRR5067222	Vietnam	2010	MDR	2.2.1
SRR5067227	Vietnam	2010	Other	2.2.1
SRR5067228	Vietnam	2010	Susceptible	2.2.1
SRR5067229	Vietnam	2010	Susceptible	2.2.1
SRR5067230	Vietnam	2010	Susceptible	2.2.1
SRR5067231	Vietnam	2010	Susceptible	2.2.1
SRR5067233	Vietnam	2010	Susceptible	2.2.1.1
SRR5067234	Vietnam	2010	Other	2.2.1
SRR5067236	Vietnam	2010	Susceptible	2.2.2
SRR5067237	Vietnam	2010	Susceptible	2.2.1
SRR5067238	Vietnam	2010	Other	2.2.1
SRR5067240	Vietnam	2010	Other	2.2.1
SRR5067241	Vietnam	2010	Susceptible	2.2.1
SRR5067243	Vietnam	2010	Other	2.2.1
SRR5067244	Vietnam	2010	Other	2.2.1
SRR5067245	Vietnam	2010	Susceptible	2.2.1
SRR5067249	Vietnam	2010	Susceptible	2.2.1
SRR5067250	Vietnam	2010	Susceptible	2.2.1
SRR5067251	Vietnam	2010	Other	2.2.1
SRR5067253	Vietnam	2010	Susceptible	2.2.1
SRR5067254	Vietnam	2010	Susceptible	2.2.1
SRR5067257	Vietnam	2010	Susceptible	2.2.1
SRR5067258	Vietnam	2010	Susceptible	2.2.1
SRR5067259	Vietnam	2010	Other	2.2.1
SRR5067260	Vietnam	2010	Other	2.2.1.2
SRR5067261	Vietnam	2010	Other	2.2.1
SRR5067264	Vietnam	2010	Other	2.2.1
SRR5067265	Vietnam	2010	Susceptible	2.2.1.1
SRR5067270	Vietnam	2010	Susceptible	2.2.1
SRR5067271	Vietnam	2010	Susceptible	2.2.1
SRR5067272	Vietnam	2010	Other	2.2.1
SRR5067273	Vietnam	2010	Other	2.2.1
SRR5067275	Vietnam	2010	Susceptible	2.2.1
SRR5067277	Vietnam	2010	Other	2.2.1.1
SRR5067278	Vietnam	2010	Susceptible	2.2.1
SRR5067282	Vietnam	2010	Other	2.2.1
SRR5067285	Vietnam	2010	Susceptible	2.1
SRR5067286	Vietnam	2010	Susceptible	2.2.1
SRR5067288	Vietnam	2010	Other	2.2.1.1
SRR5067289	Vietnam	2010	MDR	2.2.1
SRR5067290	Vietnam	2010	Susceptible	2.2.1
SRR5067292	Vietnam	2010	MDR	2.2.1

SRR5067293	Vietnam	2010	Susceptible	2.2.1
SRR5067294	Vietnam	2010	Other	2.2.1
SRR5067295	Vietnam	2010	Susceptible	2.2.1
SRR5067297	Vietnam	2010	Susceptible	2.2.1
SRR5067299	Vietnam	2010	Susceptible	2.2.2
SRR5067301	Vietnam	2010	Other	2.2.1
SRR5067302	Vietnam	2010	Other	2.2.1.1
SRR5067304	Vietnam	2010	Susceptible	2.2.1
SRR5067306	Vietnam	2010	Susceptible	2.2.1
SRR5067307	Vietnam	2010	Susceptible	2.2.1.1
SRR5067308	Vietnam	2010	Susceptible	2.2.1
SRR5067309	Vietnam	2010	MDR	2.2.1.1
SRR5067311	Vietnam	2010	Susceptible	2.2.1
SRR5067312	Vietnam	2010	Susceptible	2.2.1
SRR5067313	Vietnam	2010	Other	2.2.1
SRR5067314	Vietnam	2010	Susceptible	2.2.1
SRR5067316	Vietnam	2010	Susceptible	2.2.1
SRR5067317	Vietnam	2010	Susceptible	2.2.1.1
SRR5067319	Vietnam	2010	Susceptible	2.2.1
SRR5067321	Vietnam	2010	Other	2.2.1
SRR5067322	Vietnam	2010	Other	2.2.1
SRR5067324	Vietnam	2010	Other	2.2.1
SRR5067325	Vietnam	2010	Other	2.2.1
SRR5067326	Vietnam	2010	Susceptible	2.2.1
SRR5067329	Vietnam	2010	Other	2.2.1
SRR5067331	Vietnam	2010	Susceptible	2.2.1
SRR5067333	Vietnam	2010	Other	2.2.1
SRR5067334	Vietnam	2010	Susceptible	2.2.1.1
SRR5067335	Vietnam	2010	Susceptible	2.2.1.1
SRR5067337	Vietnam	2010	Other	2.2.1
SRR5067338	Vietnam	2010	Susceptible	2.2.1.1
SRR5067339	Vietnam	2010	Susceptible	2.2.1
SRR5067340	Vietnam	2010	Other	2.2.1
SRR5067343	Vietnam	2010	Other	2.2.1
SRR5067345	Vietnam	2009	Susceptible	2.2.1
SRR5067346	Vietnam	2010	Susceptible	2.2.1
SRR5067347	Vietnam	2010	Other	2.2.1.1
SRR5067348	Vietnam	2010	MDR	2.2.1
SRR5067351	Vietnam	2010	Susceptible	2.2.1
SRR5067354	Vietnam	2010	Other	2.2.1
SRR5067356	Vietnam	2010	Susceptible	2.2.1.1
SRR5067359	Vietnam	2010	Other	2.2.1.1
SRR5067360	Vietnam	2010	Susceptible	2.2.1.1
SRR5067366	Vietnam	2010	Susceptible	2.2.1.1
SRR5067367	Vietnam	2010	Other	2.2.1
SRR5067368	Vietnam	2010	Other	2.2.1.1
SRR5067369	Vietnam	2010	Susceptible	2.2.1
SRR5067371	Vietnam	2010	Susceptible	2.2.1
SRR5067372	Vietnam	2010	Susceptible	2.2
SRR5067374	Vietnam	2010	Susceptible	2.2.2
SRR5067375	Vietnam	2010	Other	2.2.1.1
SRR5067378	Vietnam	2010	Other	2.2.2
SRR5067379	Vietnam	2010	Other	2.2.1
SRR5067380	Vietnam	2010	Susceptible	2.2.1
SRR5067383	Vietnam	2010	Susceptible	2.2.1
SRR5067385	Vietnam	2010	Susceptible	2.2.1.1
SRR5067387	Vietnam	2010	Other	2.2.1
SRR5067388	Vietnam	2009	Other	2.2.1
SRR5067390	Vietnam	2010	Other	2.2.1
SRR5067391	Vietnam	2010	Other	2.2.1.1
SRR5067393	Vietnam	2010	Susceptible	2.2.1

SRR5067396	Vietnam	2010	Other	2.2.1
SRR5067399	Vietnam	2010	Other	2.2.1
SRR5067400	Vietnam	2010	Susceptible	2.2.1
SRR5067401	Vietnam	2010	Susceptible	2.2.1
SRR5067402	Vietnam	2010	Susceptible	2.2.1
SRR5067403	Vietnam	2010	Susceptible	2.2.1
SRR5067404	Vietnam	2010	Other	2.2.1.1
SRR5067405	Vietnam	2010	Other	2.1
SRR5067406	Vietnam	2010	Other	2.2.2
SRR5067408	Vietnam	2010	Other	2.1
SRR5067409	Vietnam	2010	Other	2.2.1
SRR5067411	Vietnam	2010	Susceptible	2.2.1
SRR5067413	Vietnam	2010	Susceptible	2.2.1
SRR5067414	Vietnam	2010	Susceptible	2.2.1
SRR5067416	Vietnam	2010	Susceptible	2.2.1
SRR5067417	Vietnam	2010	Other	2.2.1
SRR5067419	Vietnam	2010	Other	2.2.2
SRR5067420	Vietnam	2010	Other	2.2.1.1
SRR5067421	Vietnam	2010	Susceptible	2.2.1
SRR5067422	Vietnam	2010	Susceptible	2.2.1
SRR5067423	Vietnam	2010	Susceptible	2.2.1
SRR5067424	Vietnam	2010	Other	2.2.1
SRR5067425	Vietnam	2010	Other	2.2.1
SRR5067426	Vietnam	2010	Susceptible	2.2.1
SRR5067429	Vietnam	2010	Susceptible	2.2.1
SRR5067430	Vietnam	2010	Other	2.2.1.1
SRR5067431	Vietnam	2009	Susceptible	2.2.1
SRR5067432	Vietnam	2010	Susceptible	2.2.1
SRR5067433	Vietnam	2010	Other	2.1
SRR5067434	Vietnam	2010	Susceptible	2.2.1.1
SRR5067436	Vietnam	2010	Susceptible	2.2.1
SRR5067438	Vietnam	2010	Other	2.2.1.1
SRR5067439	Vietnam	2010	MDR	2.2.1
SRR5067440	Vietnam	2010	MDR	2.2.1
SRR5067441	Vietnam	2010	Other	2.2.1.1
SRR5067442	Vietnam	2009	Other	2.2.1
SRR5067443	Vietnam	2010	Susceptible	2.2.1
SRR5067444	Vietnam	2010	Other	2.2.1
SRR5067446	Vietnam	2010	Susceptible	2.2.1
SRR5067447	Vietnam	2010	Other	2.2.1
SRR5067448	Vietnam	2010	Susceptible	2.2.1
SRR5067450	Vietnam	2010	Susceptible	2.2.1.1
SRR5067451	Vietnam	2010	Susceptible	2.2.1
SRR5067454	Vietnam	2010	Susceptible	2.2.1
SRR5067456	Vietnam	2010	Susceptible	2.2.1
SRR5067457	Vietnam	2010	Susceptible	2.2.1
SRR5067458	Vietnam	2010	MDR	2.1
SRR5067460	Vietnam	2009	Susceptible	2.2.1
SRR5067461	Vietnam	2011	Other	2.1
SRR5067462	Vietnam	2010	Other	2.2.1
SRR5067465	Vietnam	2010	Other	2.2.1
SRR5067467	Vietnam	2010	Other	2.2.1
SRR5067469	Vietnam	2010	Susceptible	2.2.1
SRR5067471	Vietnam	2010	Susceptible	2.2.1
SRR5067472	Vietnam	2010	Other	2.2.1
SRR5067474	Vietnam	2010	Susceptible	2.2.1
SRR5067475	Vietnam	2010	Susceptible	2.1
SRR5067476	Vietnam	2010	Susceptible	2.2.1
SRR5067477	Vietnam	2010	Other	2.2.1
SRR5067478	Vietnam	2010	Susceptible	2.2.1
SRR5067479	Vietnam	2010	Other	2.2.1.1

SRR5067480	Vietnam	2010	Other	2.2.1
SRR5067481	Vietnam	2010	MDR	2.2.1
SRR5067482	Vietnam	2010	Other	2.2.1
SRR5067483	Vietnam	2010	Susceptible	2.2.1
SRR5067484	Vietnam	2010	Other	2.2.1.1
SRR5067489	Vietnam	2010	Susceptible	2.2.1
SRR5067490	Vietnam	2011	Other	2.2.1
SRR5067491	Vietnam	2009	Other	2.2.1
SRR5067493	Vietnam	2010	Susceptible	2.2.1
SRR5067494	Vietnam	2009	Other	2.2.1
SRR5067495	Vietnam	2010	Other	2.2.1.1
SRR5067496	Vietnam	2010	Other	2.2.1
SRR5067499	Vietnam	2010	Susceptible	2.2.1
SRR5067500	Vietnam	2010	Other	2.2.1
SRR5067501	Vietnam	2010	Susceptible	2.2.1
SRR5067502	Vietnam	2011	Other	2.2.1
SRR5067504	Vietnam	2010	Other	2.2.1.1
SRR5067505	Vietnam	2010	Susceptible	2.2.1
SRR5067510	Vietnam	2009	Susceptible	2.1
SRR5067511	Vietnam	2010	Susceptible	2.2.1
SRR5067513	Vietnam	2010	Susceptible	2.2.1
SRR5067514	Vietnam	2010	Susceptible	2.2.1
SRR5067516	Vietnam	2010	Susceptible	2.2.1
SRR5067518	Vietnam	2010	Other	2.2.1
SRR5067520	Vietnam	2010	Susceptible	2.2.1
SRR5067521	Vietnam	2010	Other	2.2.1
SRR5067523	Vietnam	2010	Susceptible	2.2.1
SRR5067525	Vietnam	2010	Susceptible	2.2.1
SRR5067527	Vietnam	2010	Susceptible	2.2.1.1
SRR5067528	Vietnam	2010	Susceptible	2.2.1
SRR5067530	Vietnam	2010	Susceptible	2.2.1
SRR5067531	Vietnam	2010	Susceptible	2.2.1
SRR5067532	Vietnam	2010	Other	2.2.1
SRR5067533	Vietnam	2010	Other	2.2.1.1
SRR5067535	Vietnam	2010	Susceptible	2.2.1
SRR5067536	Vietnam	2010	Susceptible	2.2.1
SRR5067538	Vietnam	2010	Susceptible	2.2.1.1
SRR5067541	Vietnam	2010	Susceptible	2.2.1.2
SRR5067543	Vietnam	2010	MDR	2.2.1.1
SRR5067544	Vietnam	2010	Other	2.2.1.1
SRR5067545	Vietnam	2010	Other	2.2.1
SRR5067546	Vietnam	2010	Susceptible	2.2.1
SRR5067547	Vietnam	2010	Susceptible	2.2.1
SRR5067549	Vietnam	2010	Other	2.2.1
SRR5067550	Vietnam	2010	Other	2.1
SRR5067551	Vietnam	2010	Other	2.2.1.1
SRR5067552	Vietnam	2010	Susceptible	2.2.1
SRR5067554	Vietnam	2010	Susceptible	2.1
SRR5067555	Vietnam	2009	Susceptible	2.2.1
SRR5067556	Vietnam	2010	Susceptible	2.2.1
SRR5067557	Vietnam	2010	Susceptible	2.2.1
SRR5067558	Vietnam	2010	MDR	2.2.1
SRR5067560	Vietnam	2010	Other	2.2.1
SRR5067561	Vietnam	2011	Susceptible	2.2.1
SRR5067562	Vietnam	2010	Susceptible	2.2.1
SRR5067563	Vietnam	2010	Other	2.2.1
SRR5067564	Vietnam	2010	Other	2.2.1
SRR5067566	Vietnam	2010	Susceptible	2.2.1
SRR5067567	Vietnam	2010	Susceptible	2.2.1
SRR5067568	Vietnam	2010	Susceptible	2.2.1
SRR5067570	Vietnam	2010	Susceptible	2.2.1

SRR5067571	Vietnam	2010	Other	2.2.1.1
SRR5067574	Vietnam	2010	Susceptible	2.2.1
SRR5067576	Vietnam	2010	Susceptible	2.2.1.1
SRR5067579	Vietnam	2010	Other	2.2.1
SRR5067580	Vietnam	2010	Susceptible	2.1
SRR5067582	Vietnam	2010	Other	2.2.1
SRR5067584	Vietnam	2010	Other	2.2.1
SRR5067586	Vietnam	2010	Susceptible	2.2.1
SRR5067587	Vietnam	2010	Other	2.2.1
SRR5067588	Vietnam	2010	Susceptible	2.2.1.1
SRR5067590	Vietnam	2010	Susceptible	2.2.1
SRR5067592	Vietnam	2010	Susceptible	2.2.1.1
SRR5067593	Vietnam	2010	Susceptible	2.2.1
SRR5067594	Vietnam	2010	Susceptible	2.2.1
SRR5067595	Vietnam	2010	Susceptible	2.2.1
SRR5067600	Vietnam	2010	Susceptible	2.2.1
SRR5067601	Vietnam	2010	Other	2.2.1
SRR5067603	Vietnam	2010	Susceptible	2.2.1
SRR5067605	Vietnam	2010	Other	2.2.1.1
SRR5067606	Vietnam	2009	Susceptible	2.2.1.1
SRR5067608	Vietnam	2010	Other	2.2.1.1
SRR5067609	Vietnam	2010	Susceptible	2.2.1
SRR5067611	Vietnam	2010	Other	2.2.1.1
SRR5067612	Vietnam	2010	Susceptible	2.2.1.1
SRR5067613	Vietnam	2010	Other	2.2.1
SRR5067614	Vietnam	2010	Other	2.2.1
SRR5067616	Vietnam	2010	Susceptible	2.2.1
SRR5067617	Vietnam	2010	Other	2.2.1
SRR5067618	Vietnam	2010	Susceptible	2.2.1
SRR5067619	Vietnam	2010	Susceptible	2.2.1
SRR5067620	Vietnam	2010	Susceptible	2.2.1
SRR5067623	Vietnam	2010	Other	2.2.1
SRR5067624	Vietnam	2010	Susceptible	2.2.2
SRR5067626	Vietnam	2010	Susceptible	2.2.1
SRR5067627	Vietnam	2010	Susceptible	2.2.1
SRR5067629	Vietnam	2010	Susceptible	2.2.1.1
SRR5067630	Vietnam	2010	Susceptible	2.2.1
SRR5067631	Vietnam	2010	Susceptible	2.2.1
SRR5067632	Vietnam	2010	Other	2.2.1
SRR5067635	Vietnam	2009	Other	2.2.1
SRR5067636	Vietnam	2010	Susceptible	2.2.1.1
SRR5067637	Vietnam	2010	Other	2.2.1
SRR5067638	Vietnam	2010	Other	2.2.1.1
SRR5067639	Vietnam	2010	Other	2.2.1
SRR5067640	Vietnam	2010	Susceptible	2.2.1
SRR5067642	Vietnam	2009	Susceptible	2.1
SRR5067643	Vietnam	2010	Susceptible	2.2.1.1
SRR5067646	Vietnam	2010	Other	2.2.1.1
SRR5067649	Vietnam	2010	Other	2.2.1
SRR5067653	Vietnam	2011	Susceptible	2.2.1
SRR5067654	Vietnam	2010	Other	2.2.1
SRR5067657	Vietnam	2010	Other	2.2.1
SRR5067658	Vietnam	2010	Other	2.2.1
SRR5067659	Vietnam	2010	Susceptible	2.2.1
SRR5067660	Vietnam	2010	Susceptible	2.2.1.1
SRR5067661	Vietnam	2010	Susceptible	2.2.1
SRR5067665	Vietnam	2010	Other	2.2.1
SRR5067666	Vietnam	2010	Susceptible	2.2.1
SRR5067670	Vietnam	2010	Susceptible	2.2.1
SRR5067672	Vietnam	2010	Susceptible	2.2.1
SRR5067673	Vietnam	2010	Other	2.2.1

SRR5067675	Vietnam	2009	Susceptible	2.2.2
SRR5067676	Vietnam	2010	Other	2.2.1.1
SRR5067677	Vietnam	2010	Other	2.2.1
SRR5067678	Vietnam	2010	Susceptible	2.2.1.1
SRR5067679	Vietnam	2010	Susceptible	2.2.1
SRR5067682	Vietnam	2010	Other	2.2.1
SRR5067683	Vietnam	2010	Other	2.1
SRR5067684	Vietnam	2010	Susceptible	2.2.1
SRR5067686	Vietnam	2011	Other	2.2.1
SRR5067687	Vietnam	2010	Susceptible	2.2.1
SRR5067688	Vietnam	2010	Susceptible	2.2.1
SRR5067689	Vietnam	2010	Susceptible	2.2.1
SRR5067690	Vietnam	2010	Susceptible	2.2.1
SRR5067691	Vietnam	2010	Other	2.2.1
SRR5067692	Vietnam	2010	Susceptible	2.2.1
SRR5067693	Vietnam	2010	MDR	2.2.1
SRR5067694	Vietnam	2010	Other	2.2.1
SRR5067695	Vietnam	2010	Other	2.2.1
SRR5067696	Vietnam	2010	Other	2.2.1
SRR5067697	Vietnam	2010	Susceptible	2.2.1
SRR5067699	Vietnam	2010	Susceptible	2.2.1.1
SRR5067700	Vietnam	2010	Other	2.2.1.1
SRR5067701	Vietnam	2010	Other	2.2.2
SRR5067702	Vietnam	2010	Susceptible	2.2.1.1
SRR5067704	Vietnam	2010	MDR	2.2.1
SRR5067705	Vietnam	2009	Other	2.2.1
SRR5067706	Vietnam	2010	Susceptible	2.2.1
SRR5067707	Vietnam	2010	Other	2.2.1
SRR5067710	Vietnam	2010	Susceptible	2.2.1
SRR5067711	Vietnam	2009	Other	2.2.1
SRR5067712	Vietnam	2010	Susceptible	2.2.1
SRR5067713	Vietnam	2010	Susceptible	2.1
SRR5067714	Vietnam	2010	Susceptible	2.2.1
SRR5067715	Vietnam	2010	Susceptible	2.2.2
SRR5067716	Vietnam	2010	Susceptible	2.2.2
SRR5067717	Vietnam	2010	Susceptible	2.2.1.1
SRR5067718	Vietnam	2010	Other	2.2.1.1
SRR5073499	Vietnam	2009	Susceptible	2.1
SRR5073500	Vietnam	2009	Susceptible	2.2.1
SRR5073504	Vietnam	2009	Susceptible	2.2.1
SRR5073505	Vietnam	2009	Other	2.2.1
SRR5073506	Vietnam	2009	Other	2.2.1
SRR5073507	Vietnam	2010	Susceptible	2.2.1
SRR5073508	Vietnam	2009	Susceptible	2.2.1
SRR5073509	Vietnam	2009	Susceptible	2.2.1
SRR5073513	Vietnam	2009	Susceptible	2.2.1
SRR5073514	Vietnam	2009	Susceptible	2.2.1.1
SRR5073515	Vietnam	2009	Susceptible	2.2.1
SRR5073519	Vietnam	2009	Susceptible	2.2.1
SRR5073520	Vietnam	2010	Other	2.2.1
SRR5073521	Vietnam	2009	MDR	2.2.1
SRR5073522	Vietnam	2009	Other	2.2.1
SRR5073523	Vietnam	2009	Other	2.2.1
SRR5073524	Vietnam	2009	Susceptible	2.2.1
SRR5073526	Vietnam	2010	Susceptible	2.2.1
SRR5073527	Vietnam	2009	Other	2.2.1
SRR5073530	Vietnam	2009	Susceptible	2.2.1
SRR5073532	Vietnam	2009	Susceptible	2.2.1
SRR5073533	Vietnam	2009	MDR	2.2.1
SRR5073534	Vietnam	2010	MDR	2.2.1.1
SRR5073535	Vietnam	2009	Susceptible	2.2.1

SRR5073536	Vietnam	2009	Susceptible	2.2.1
SRR5073537	Vietnam	2010	Susceptible	2.2.1
SRR5073538	Vietnam	2009	Other	2.2.1.1
SRR5073543	Vietnam	2009	Susceptible	2.2.1
SRR5073546	Vietnam	2009	Susceptible	2.2.1
SRR5073547	Vietnam	2009	Other	2.2.1
SRR5073548	Vietnam	2009	Susceptible	2.1
SRR5073549	Vietnam	2009	Susceptible	2.2.2
SRR5073550	Vietnam	2009	Other	2.2.1
SRR5073555	Vietnam	2009	MDR	2.2.1
SRR5073562	Vietnam	2009	Susceptible	2.2.2
SRR5073563	Vietnam	2009	Susceptible	2.2.1
SRR5073564	Vietnam	2009	Other	2.2.1.1
SRR5073567	Vietnam	2009	Susceptible	2.2.1
SRR5073568	Vietnam	2009	Other	2.2.1
SRR5073569	Vietnam	2009	Susceptible	2.2.1
SRR5073571	Vietnam	2009	Susceptible	2.2.1
SRR5073573	Vietnam	2009	Susceptible	2.2.1.1
SRR5073574	Vietnam	2009	Susceptible	2.2.1
SRR5073575	Vietnam	2009	Other	2.2.1
SRR5073576	Vietnam	2009	Susceptible	2.2.1
SRR5073577	Vietnam	2009	Other	2.2.1.1
SRR5073578	Vietnam	2009	Other	2.2.1
SRR5073579	Vietnam	2009	Susceptible	2.2.1
SRR5073581	Vietnam	2009	Susceptible	2.2.1
SRR5073582	Vietnam	2009	Susceptible	2.2.1
SRR5073583	Vietnam	2009	Other	2.2.1.1
SRR5073585	Vietnam	2009	Other	2.2.1
SRR5073586	Vietnam	2009	Other	2.2.2
SRR5073588	Vietnam	2009	Susceptible	2.2.1
SRR5073589	Vietnam	2009	Other	2.2.1.1
SRR5073591	Vietnam	2009	Other	2.2.1
SRR5073593	Vietnam	2009	Other	2.2.1.1
SRR5073594	Vietnam	2009	Susceptible	2.2.1.1
SRR5073595	Vietnam	2009	Susceptible	2.2.1
SRR5073596	Vietnam	2009	Susceptible	2.2.1
SRR5073597	Vietnam	2009	Susceptible	2.2.1
SRR5073598	Vietnam	2009	Susceptible	2.2.1
SRR5073599	Vietnam	2009	Other	2.2.1.1
SRR5073600	Vietnam	2009	Susceptible	2.2.1.1
SRR5073601	Vietnam	2009	Other	2.2.1.1
SRR5073602	Vietnam	2009	Susceptible	2.1
SRR5073603	Vietnam	2009	MDR	2.2.1
SRR5073605	Vietnam	2009	Other	2.2.1
SRR5073607	Vietnam	2009	Susceptible	2.2.1
SRR5073608	Vietnam	2009	Other	2.2.1
SRR5073610	Vietnam	2009	Other	2.2.1
SRR5073611	Vietnam	2009	Susceptible	2.2.1
SRR5073612	Vietnam	2009	Susceptible	2.2.1
SRR5073613	Vietnam	2009	Other	2.2.1.1
SRR5073615	Vietnam	2009	Susceptible	2.2.1
SRR5073616	Vietnam	2009	Susceptible	2.2.1
SRR5073618	Vietnam	2009	Other	2.2.1
SRR5073619	Vietnam	2010	Other	2.2.1.1
SRR5073622	Vietnam	2009	Other	2.2.1
SRR5073625	Vietnam	2009	Other	2.2.1.1
SRR5073626	Vietnam	2009	Susceptible	2.2.1
SRR5073628	Vietnam	2009	Susceptible	2.2.1
SRR5073629	Vietnam	2009	Other	2.2.1
SRR5073630	Vietnam	2009	Other	2.2.1
SRR5073631	Vietnam	2010	MDR	2.2.1

SRR5073632	Vietnam	2009	Susceptible	2.2.1
SRR5073633	Vietnam	2009	Susceptible	2.2.2
SRR5073636	Vietnam	2009	Susceptible	2.2.1
SRR5073637	Vietnam	2009	Other	2.1
SRR5073638	Vietnam	2009	Other	2.2.1
SRR5073641	Vietnam	2009	Other	2.2.1.1
SRR5073642	Vietnam	2009	Susceptible	2.2.1
SRR5073643	Vietnam	2010	Susceptible	2.2.2
SRR5073644	Vietnam	2010	Other	2.2.1.1
SRR5073646	Vietnam	2009	Susceptible	2.2.1
SRR5073647	Vietnam	2009	Other	2.2.1
SRR5073648	Vietnam	2009	Other	2.2.1
SRR5073649	Vietnam	2009	Other	2.2.1
SRR5073650	Vietnam	2009	Susceptible	2.2.1
SRR5073651	Vietnam	2009	Other	2.2.1
SRR5073652	Vietnam	2009	Other	2.2.1
SRR5073654	Vietnam	2010	Other	2.2.1
SRR5073655	Vietnam	2009	Susceptible	2.2.1
SRR5073656	Vietnam	2009	Susceptible	2.1
SRR5073657	Vietnam	2009	Susceptible	2.2.1
SRR5073658	Vietnam	2009	Other	2.2.1
SRR5073659	Vietnam	2009	Susceptible	2.2.1
SRR5073660	Vietnam	2009	Other	2.2.2
SRR5073661	Vietnam	2009	Other	2.2.1
SRR5073662	Vietnam	2009	Susceptible	2.2.1
SRR5073664	Vietnam	2009	Other	2.2.1
SRR5073666	Vietnam	2009	Susceptible	2.2.1
SRR5073669	Vietnam	2009	Susceptible	2.2.1
SRR5073670	Vietnam	2009	Susceptible	2.2.1
SRR5073671	Vietnam	2009	Other	2.2.1
SRR5073672	Vietnam	2009	Other	2.2.1.1
SRR5073673	Vietnam	2009	Other	2.2.1
SRR5073676	Vietnam	2009	Other	2.2.1
SRR5073677	Vietnam	2009	Other	2.2.1
SRR5073680	Vietnam	2009	Other	2.2.1
SRR5073681	Vietnam	2009	Other	2.2.1
SRR5073683	Vietnam	2009	Susceptible	2.2.1.1
SRR5073685	Vietnam	2010	Susceptible	2.2.1
SRR5073687	Vietnam	2009	Susceptible	2.2.1
SRR5073689	Vietnam	2009	Susceptible	2.2.2
SRR5073694	Vietnam	2009	MDR	2.2.1
SRR5073695	Vietnam	2009	Other	2.2.1
SRR5073696	Vietnam	2009	Susceptible	2.2.1
SRR5073697	Vietnam	2010	Susceptible	2.2.1
SRR5073698	Vietnam	2009	MDR	2.2.1.1
SRR5073699	Vietnam	2009	Susceptible	2.2.1
SRR5073701	Vietnam	2009	Susceptible	2.2.1
SRR5073702	Vietnam	2009	Susceptible	2.2.1
SRR5073703	Vietnam	2009	Other	2.2.1
SRR5073704	Vietnam	2009	Other	2.2.1
SRR5073707	Vietnam	2009	Other	2.2.1
SRR5073708	Vietnam	2009	Other	2.2.1
SRR5073709	Vietnam	2009	Other	2.2.1
SRR5073710	Vietnam	2009	Susceptible	2.1
SRR5073712	Vietnam	2009	Susceptible	2.2.1
SRR5073713	Vietnam	2009	Susceptible	2.2.1
SRR5073714	Vietnam	2009	Susceptible	2.2.1
SRR5073716	Vietnam	2009	Other	2.2.1
SRR5073717	Vietnam	2010	Susceptible	2.2.1
SRR5073718	Vietnam	2009	Other	2.2.1.1
SRR5073719	Vietnam	2009	Susceptible	2.2.1

SRR5073721	Vietnam	2009	Other	2.2.1
SRR5073723	Vietnam	2009	Other	2.2.1
SRR5073727	Vietnam	2009	Other	2.2.1
SRR5073728	Vietnam	2009	Other	2.2.1.1
SRR5073730	Vietnam	2009	Susceptible	2.2.1
SRR5073731	Vietnam	2009	Other	2.2.1
SRR5073732	Vietnam	2009	Other	2.2.1
SRR5073735	Vietnam	2009	Susceptible	2.2.1
SRR5073736	Vietnam	2009	Other	2.2.1
SRR5073737	Vietnam	2009	Other	2.2.1
SRR5073738	Vietnam	2009	Susceptible	2.2.1
SRR5073739	Vietnam	2009	Other	2.1
SRR5073740	Vietnam	2009	Susceptible	2.2.1
SRR5073741	Vietnam	2009	Susceptible	2.2.1
SRR5073742	Vietnam	2009	Susceptible	2.2.1
SRR5073743	Vietnam	2009	Other	2.2.1
SRR5073744	Vietnam	2009	Other	2.2.1.1
SRR5073745	Vietnam	2009	Susceptible	2.2.1
SRR5073747	Vietnam	2009	Susceptible	2.2.1
SRR5073750	Vietnam	2009	Other	2.2.1.1
SRR5073755	Vietnam	2010	Susceptible	2.2.1
SRR5073757	Vietnam	2009	Susceptible	2.2.1
SRR5073758	Vietnam	2009	Susceptible	2.2.1
SRR5073759	Vietnam	2009	Other	2.2.1.1
SRR5073760	Vietnam	2009	Susceptible	2.2.1
SRR5073761	Vietnam	2009	Susceptible	2.1
SRR5073762	Vietnam	2009	Susceptible	2.2.1
SRR5073764	Vietnam	2009	MDR	2.2.1
SRR5073766	Vietnam	2010	Susceptible	2.2.1
SRR5073767	Vietnam	2010	Susceptible	2.2.1
SRR5073768	Vietnam	2009	Susceptible	2.2.1
SRR5073771	Vietnam	2009	Other	2.2.1
SRR5073772	Vietnam	2009	Other	2.2.1.1
SRR5073773	Vietnam	2009	Susceptible	2.2.1
SRR5073774	Vietnam	2009	Susceptible	2.2.1
SRR5073776	Vietnam	2009	Susceptible	2.2.1
SRR5073777	Vietnam	2009	Susceptible	2.2.1
SRR5073779	Vietnam	2009	Susceptible	2.2.1
SRR5073780	Vietnam	2009	Other	2.2.1
SRR5073782	Vietnam	2009	Other	2.2.1
SRR5073783	Vietnam	2009	Susceptible	2.2.1
SRR5073786	Vietnam	2009	Susceptible	2.2.2
SRR5073787	Vietnam	2010	MDR	2.2.1
SRR5073790	Vietnam	2009	Susceptible	2.2.1
SRR5073791	Vietnam	2009	Other	2.2.1
SRR5073794	Vietnam	2009	Other	2.2.1
SRR5073796	Vietnam	2009	Other	2.2.1
SRR5073799	Vietnam	2010	Other	2.2.1
SRR5073800	Vietnam	2009	Susceptible	2.2.1
SRR5073802	Vietnam	2009	Susceptible	2.2.1
SRR5073803	Vietnam	2009	Susceptible	2.2.1
SRR5073807	Vietnam	2009	Susceptible	2.2.1
SRR5073809	Vietnam	2010	Susceptible	2.2
SRR5073810	Vietnam	2009	MDR	2.2.1
SRR5073814	Vietnam	2009	Other	2.2.1
SRR5073815	Vietnam	2009	Susceptible	2.2.1
SRR5073818	Vietnam	2009	MDR	2.2.2
SRR5073819	Vietnam	2009	Susceptible	2.2.1
SRR5073821	Vietnam	2009	Other	2.2.1
SRR5073822	Vietnam	2009	Other	2.1
SRR5073823	Vietnam	2009	Other	2.2.1

SRR5073824	Vietnam	2009	Susceptible	2.2.2
SRR5073828	Vietnam	2009	Susceptible	2.2.1
SRR5073829	Vietnam	2009	Susceptible	2.2.1
SRR5073830	Vietnam	2009	Other	2.2.1
SRR5073833	Vietnam	2009	Other	2.2.1
SRR5073834	Vietnam	2009	Other	2.2.1
SRR5073835	Vietnam	2009	Susceptible	2.2.1
SRR5073836	Vietnam	2010	Susceptible	2.2.1.1
SRR5073838	Vietnam	2009	Susceptible	2.1
SRR5073839	Vietnam	2009	Other	2.2.1
SRR5073843	Vietnam	2009	Susceptible	2.2.1
SRR5073845	Vietnam	2009	Susceptible	2.2.1.1
SRR5073852	Vietnam	2009	Susceptible	2.2.1
SRR5073853	Vietnam	2009	Other	2.2.1
SRR5073854	Vietnam	2009	MDR	2.2.1
SRR5073856	Vietnam	2009	Susceptible	2.2.1
SRR5073859	Vietnam	2009	Susceptible	2.2.1
SRR5073860	Vietnam	2009	Susceptible	2.2.2
SRR5073862	Vietnam	2009	Susceptible	2.2.1
SRR5073863	Vietnam	2009	Other	2.2.1
SRR5073864	Vietnam	2009	Other	2.2.1
SRR5073865	Vietnam	2009	Other	2.2.1
SRR5073866	Vietnam	2009	Susceptible	2.1
SRR5073867	Vietnam	2009	Susceptible	2.2.1
SRR5073868	Vietnam	2009	Other	2.2.1
SRR5073870	Vietnam	2009	MDR	2.2.1
SRR5073872	Vietnam	2009	Susceptible	2.2.1
SRR5073873	Vietnam	2009	Other	2.2.1
SRR5073875	Vietnam	2009	Susceptible	2.2.1
SRR5073878	Vietnam	2009	Susceptible	2.2.1
SRR5073879	Vietnam	2009	Susceptible	2.2.1
SRR5073880	Vietnam	2009	Susceptible	2.2.1
SRR5073882	Vietnam	2009	Other	2.1
SRR5073883	Vietnam	2010	Susceptible	2.1
SRR5073884	Vietnam	2009	Other	2.2.1
SRR5073888	Vietnam	2009	Susceptible	2.2.1
SRR5073890	Vietnam	2009	Other	2.2.1.1
SRR5073892	Vietnam	2009	Other	2.2.1.1
SRR5073893	Vietnam	2009	Other	2.2.1.1
SRR5073895	Vietnam	2009	Susceptible	2.2.1
SRR5073900	Vietnam	2009	Other	2.2.1
SRR5073902	Vietnam	2009	Other	2.1
SRR5073903	Vietnam	2010	Susceptible	2.2.1
SRR5073904	Vietnam	2009	Susceptible	2.2.1
SRR5073906	Vietnam	2009	Susceptible	2.2.1
SRR5073907	Vietnam	2009	Susceptible	2.2.1
SRR5073909	Vietnam	2009	Other	2.2.1
SRR5073911	Vietnam	2009	Susceptible	2.2.1
SRR5073913	Vietnam	2009	Other	2.2.1
SRR5073914	Vietnam	2009	Susceptible	2.2.1
SRR5073916	Vietnam	2009	Susceptible	2.2.1
SRR5073917	Vietnam	2009	Other	2.2.1
SRR5073918	Vietnam	2009	Susceptible	2.2.1.1
SRR5073920	Vietnam	2009	Other	2.2.1
SRR5073921	Vietnam	2009	Susceptible	2.1
SRR5073922	Vietnam	2009	Other	2.2.1
SRR5073924	Vietnam	2009	Other	2.2.1.1
SRR5073927	Vietnam	2009	Susceptible	2.2.1.1
SRR5073928	Vietnam	2009	Susceptible	2.2.1
SRR5073929	Vietnam	2009	Susceptible	2.2.1
SRR5073931	Vietnam	2009	Other	2.2.1

SRR5073933	Vietnam	2009	Other	2.2.1
SRR5073935	Vietnam	2009	Other	2.2.1
SRR5073936	Vietnam	2009	Susceptible	2.2.1
SRR5073937	Vietnam	2009	Susceptible	2.2.1
SRR5073938	Vietnam	2009	Susceptible	2.2.1
SRR5073941	Vietnam	2009	Other	2.2.1
SRR5073942	Vietnam	2009	Susceptible	2.2.1
SRR5073943	Vietnam	2009	Susceptible	2.2.2
SRR5073945	Vietnam	2009	Susceptible	2.2.1.1
SRR5073946	Vietnam	2009	Susceptible	2.2.1.1
SRR5073948	Vietnam	2009	Other	2.2.1
SRR5073949	Vietnam	2009	Susceptible	2.2.1
SRR5073951	Vietnam	2009	Susceptible	2.2.1
SRR5073953	Vietnam	2010	Susceptible	2.2.1
SRR5073958	Vietnam	2009	Other	2.2.1
SRR5073959	Vietnam	2009	Other	2.2.2
SRR5073961	Vietnam	2009	Other	2.2.1
SRR5073962	Vietnam	2009	Susceptible	2.2.1
SRR5073963	Vietnam	2009	Susceptible	2.2.1.1
SRR5073964	Vietnam	2009	Other	2.2.1
SRR5073965	Vietnam	2009	Other	2.2.1
SRR5073968	Vietnam	2009	Susceptible	2.2.1
SRR5073970	Vietnam	2009	Other	2.2.1
SRR5073972	Vietnam	2009	MDR	2.2.1
SRR5073974	Vietnam	2009	Susceptible	2.2.1.1
SRR5073975	Vietnam	2009	Susceptible	2.2.1
SRR5073976	Vietnam	2009	Susceptible	2.2.1
SRR5073982	Vietnam	2009	MDR	2.2.1
SRR5073983	Vietnam	2009	Susceptible	2.2.1
SRR5073984	Vietnam	2009	Other	2.2.1
SRR5073985	Vietnam	2009	Susceptible	2.2.1
SRR5073992	Vietnam	2009	Susceptible	2.2.1
SRR5073994	Vietnam	2009	Susceptible	2.2.1
SRR5073995	Vietnam	2009	Susceptible	2.1
SRR5073996	Vietnam	2009	Susceptible	2.2.1
SRR5073997	Vietnam	2009	Susceptible	2.2.1
SRR5073998	Vietnam	2009	MDR	2.2.1.1
SRR5074000	Vietnam	2009	Other	2.2.1
SRR5074053	Vietnam	2009	Other	2.2.1
SRR5074054	Vietnam	2009	Other	2.1
SRR5074055	Vietnam	2009	Susceptible	2.2.1
SRR5074058	Vietnam	2009	Other	2.2.1.1
SRR5074059	Vietnam	2009	Susceptible	2.2.1
SRR5074060	Vietnam	2009	Susceptible	2.2.1
SRR5074061	Vietnam	2008	Susceptible	2.2.1
SRR5074062	Vietnam	2009	Susceptible	2.2.1
SRR5074063	Vietnam	2009	Susceptible	2.2.1
SRR5074065	Vietnam	2009	Other	2.2.1
SRR5074066	Vietnam	2009	Susceptible	2.2.1
SRR5074067	Vietnam	2009	Other	2.2.1
SRR5074068	Vietnam	2009	Other	2.1
SRR5074070	Vietnam	2009	Other	2.2.1
SRR5074071	Vietnam	2009	Susceptible	2.2.1
SRR5074073	Vietnam	2008	Other	2.2.1.1
SRR5074074	Vietnam	2009	MDR	2.2.1
SRR5074076	Vietnam	2009	Other	2.2.1
SRR5074078	Vietnam	2009	Susceptible	2.2.1
SRR5074080	Vietnam	2009	Susceptible	2.2.1
SRR5074081	Vietnam	2009	Susceptible	2.2.1.1
SRR5074082	Vietnam	2009	Susceptible	2.2.1
SRR5074087	Vietnam	2009	Other	2.2.1

SRR5074088	Vietnam	2009	Susceptible	2.2.1
SRR5074089	Vietnam	2009	Other	2.2.1.1
SRR5074091	Vietnam	2009	Other	2.2.1
SRR5074093	Vietnam	2009	Susceptible	2.1
SRR5074094	Vietnam	2009	Susceptible	2.2.1
SRR5074095	Vietnam	2009	Susceptible	2.2.1
SRR5074096	Vietnam	2009	Other	2.2.1
SRR5074098	Vietnam	2009	Susceptible	2.2.1
SRR5074100	Vietnam	2008	Susceptible	2.2.1
SRR5074103	Vietnam	2009	Susceptible	2.2.2
SRR5074105	Vietnam	2008	Other	2.2.1
SRR5074107	Vietnam	2009	Other	2.2.1.1
SRR5074108	Vietnam	2009	Susceptible	2.2.1
SRR5074110	Vietnam	2009	Susceptible	2.2.1
SRR5074112	Vietnam	2009	Other	2.2.1
SRR5074113	Vietnam	2009	Susceptible	2.2.1
SRR5074114	Vietnam	2009	Other	2.2.1
SRR5074116	Vietnam	2009	Susceptible	2.2.1
SRR5074122	Vietnam	2009	Other	2.2.2
SRR5074123	Vietnam	2009	MDR	2.2.1
SRR5074125	Vietnam	2009	Susceptible	2.2.1
SRR5074127	Vietnam	2009	Susceptible	2.2.1.1
SRR5074130	Vietnam	2009	Other	2.2.1.1
SRR5074136	Vietnam	2009	Susceptible	2.1
SRR5074137	Vietnam	2009	Susceptible	2.2.1
SRR5074138	Vietnam	2008	Susceptible	2.2.2
SRR5074139	Vietnam	2009	Susceptible	2.2.2
SRR5074140	Vietnam	2009	Susceptible	2.2.1
SRR5074144	Vietnam	2009	Susceptible	2.2.1
SRR5074146	Vietnam	2009	MDR	2.2.1
SRR5074147	Vietnam	2009	Susceptible	2.2.1
SRR5074148	Vietnam	2008	Susceptible	2.2.1
SRR5074149	Vietnam	2009	Other	2.2.1
SRR5074150	Vietnam	2009	Susceptible	2.2.2
SRR5074153	Vietnam	2009	Susceptible	2.2.1
SRR5074154	Vietnam	2008	Susceptible	2.2.1.2
SRR5074155	Vietnam	2009	MDR	2.2.1
SRR5074156	Vietnam	2009	Susceptible	2.2.1
SRR5074157	Vietnam	2008	Other	2.2.1
SRR5074158	Vietnam	2009	Susceptible	2.2.1
SRR5074160	Vietnam	2009	Susceptible	2.2.1
SRR5074163	Vietnam	2009	Susceptible	2.2.1
SRR5074164	Vietnam	2009	Susceptible	2.2.1.1
SRR5074166	Vietnam	2009	Other	2.2.1
SRR5074168	Vietnam	2008	Other	2.2.1
SRR5074169	Vietnam	2009	Susceptible	2.2.1.1
SRR5074170	Vietnam	2008	Other	2.2.1
SRR5074171	Vietnam	2009	Other	2.2.1
SRR5074173	Vietnam	2009	Other	2.2.1.1
SRR5074174	Vietnam	2009	Susceptible	2.1
SRR5074175	Vietnam	2009	Susceptible	2.2.1
SRR5074176	Vietnam	2009	Other	2.2.1
SRR5074179	Vietnam	2009	Other	2.2.1
SRR5074180	Vietnam	2009	Susceptible	2.2.1
SRR5074181	Vietnam	2009	Other	2.2.1
SRR5074182	Vietnam	2009	Susceptible	2.1
SRR5074183	Vietnam	2008	Other	2.2.1
SRR5074184	Vietnam	2009	Susceptible	2.2.1
SRR5074185	Vietnam	2009	MDR	2.2.1
SRR5074188	Vietnam	2009	Susceptible	2.2.1
SRR5074189	Vietnam	2009	Susceptible	2.2.1

SRR5074191	Vietnam	2009	Other	2.2.1.1
SRR5074193	Vietnam	2009	Susceptible	2.2.1
SRR5114017	Thailand	04-Apr-2012	MDR	2.1
SRR5114018	Thailand	06-Oct-2011	MDR	2.1
SRR5114019	Thailand	26-Sep-2007	MDR	2.1
SRR5114020	Thailand	15-Oct-2008	MDR	2.1
SRR5114021	Thailand	26-Sep-2012	MDR	2.1
SRR5114022	Thailand	03-May-2008	MDR	2.1
SRR5152895	Georgia	2015	MDR	2.2.1
SRR5152896	Georgia	2015	MDR	2.2.1
SRR5152897	Georgia	2015	MDR	2.2.1
SRR5152898	Georgia	2016	MDR	2.2.1
SRR5152902	Georgia	2015	MDR	2.2.1
SRR5152903	Georgia	2015	XDR	2.2.1
SRR5152904	Georgia	2015	Other	2.2.1
SRR5152905	Georgia	2015	MDR	2.2.1
SRR5152906	Georgia	2015	MDR	2.2.1
SRR5152907	Georgia	2015	MDR	2.2.1
SRR5152908	Georgia	2015	MDR	2.2.1
SRR5152909	Georgia	2015	XDR	2.2.1
SRR5152910	Georgia	2015	MDR	2.2.1
SRR5152912	Georgia	2015	XDR	2.2.1
SRR5152914	Georgia	2015	MDR	2.2.1
SRR5152917	Georgia	2015	MDR	2.2.1
SRR5152918	Georgia	2015	Other	2.2.1
SRR5152919	Georgia	2015	XDR	2.2.1
SRR5152920	Georgia	2015	MDR	2.2.1
SRR5152921	Georgia	2015	XDR	2.2.1
SRR5152922	Georgia	2015	Other	2.2.1
SRR5152923	Georgia	2015	MDR	2.2.1
SRR5152924	Georgia	2014	MDR	2.2.1
SRR5152925	Georgia	2015	MDR	2.2.1
SRR5152926	Georgia	2015	Other	2.2.1
SRR5152930	Georgia	2015	MDR	2.2.1
SRR5152936	Georgia	2015	MDR	2.2.1
SRR5152937	Georgia	2015	MDR	2.2.1
SRR5152939	Georgia	2014	MDR	2.2.1
SRR5152940	Azerbaijan	2016	MDR	2.2.1
SRR5152941	Georgia	2015	XDR	2.2.1
SRR5152942	Azerbaijan	2016	MDR	2.2.1
SRR5152944	Azerbaijan	2016	MDR	2.2.1
SRR5152945	Azerbaijan	2015	MDR	2.2.1
SRR5152946	Azerbaijan	2016	Other	2.2.1
SRR5152947	Azerbaijan	2016	XDR	2.2.1
SRR5152948	Azerbaijan	2016	MDR	2.2.1
SRR5152949	Azerbaijan	2016	Other	2.2.1
SRR5152951	Georgia	2015	MDR	2.2.1
SRR5152952	Azerbaijan	2016	XDR	2.2.1
SRR5152953	Georgia	2015	MDR	2.2.1
SRR5152954	Georgia	2015	MDR	2.2.1
SRR5152956	Georgia	2015	MDR	2.2.1
SRR5152957	Azerbaijan	2015	MDR	2.2.1
SRR5152958	Azerbaijan	2016	Other	2.2.1
SRR5152959	Georgia	2015	MDR	2.2.1
SRR5152963	Azerbaijan	2015	Other	2.2.1
SRR5153032	Azerbaijan	2016	Susceptible	2.2.1
SRR5153072	Azerbaijan	2016	Other	2.2.1
SRR5153074	Azerbaijan	2016	MDR	2.2.1
SRR5153076	Azerbaijan	2016	Susceptible	2.2.1
SRR5153078	Azerbaijan	2016	MDR	2.2.1
SRR5153079	Georgia	2015	Other	2.2.1

SRR5153080	Azerbaijan	2016	Other	2.2.1
SRR5153081	Georgia	2015	Other	2.2.1
SRR5153082	Azerbaijan	2015	Other	2.2.1
SRR5153083	Azerbaijan	2016	Susceptible	2.2.1
SRR5153085	Azerbaijan	2016	MDR	2.2.1
SRR5153086	Azerbaijan	2016	MDR	2.2.1
SRR5153087	Azerbaijan	2015	XDR	2.2.1
SRR5153090	Georgia	2015	Susceptible	2.2.1
SRR5153092	Georgia	2015	MDR	2.2.1
SRR5153093	Georgia	2015	MDR	2.2.1
SRR5153094	Georgia	2015	XDR	2.2.1
SRR5153206	Georgia	2015	Other	2.2.1
SRR5153208	Georgia	2015	MDR	2.2.1
SRR5153214	Georgia	2014	MDR	2.2.1
SRR5153217	Georgia	2015	MDR	2.2.1
SRR5153218	Georgia	2015	MDR	2.2.1
SRR5153220	Georgia	2014	XDR	2.2.1
SRR5153221	Georgia	2015	Other	2.2.1
SRR5153222	Georgia	2014	MDR	2.2.1
SRR5153223	Georgia	2014	MDR	2.2.1
SRR5153224	Georgia	2015	Other	2.2.1
SRR5153225	Georgia	2014	MDR	2.2.1
SRR5153226	Georgia	2014	MDR	2.2.1
SRR5153227	Georgia	2014	MDR	2.2.1
SRR5153228	Georgia	2014	MDR	2.2.1
SRR5153229	Georgia	2014	MDR	2.2.1
SRR5153231	Georgia	2014	MDR	2.2.1
SRR5153232	Georgia	2014	Other	2.2.1
SRR5153233	Georgia	2014	MDR	2.2.1
SRR5153234	Georgia	2015	XDR	2.2.1
SRR5153235	Georgia	2015	MDR	2.2.1
SRR5153237	Georgia	2014	Other	2.2.1
SRR5153242	Georgia	2015	MDR	2.2.1
SRR5153245	Georgia	2015	MDR	2.2.1
SRR5153253	Georgia	2014	MDR	2.2.1
SRR5153254	Georgia	2014	XDR	2.2.1
SRR5153255	Georgia	2014	MDR	2.2.1
SRR5153256	Georgia	2014	Other	2.2.1
SRR5153259	Georgia	2014	Other	2.2.1
SRR5153261	Georgia	2014	XDR	2.2.1
SRR5153262	Georgia	2014	MDR	2.2.1
SRR5153263	Georgia	2014	Other	2.2.1
SRR5153264	Georgia	2014	MDR	2.2.1
SRR5153265	Georgia	2015	Other	2.2.1
SRR5153268	Georgia	2014	MDR	2.2.1
SRR5153269	Georgia	2014	MDR	2.2.1
SRR5153270	Georgia	2014	MDR	2.2.1
SRR5153271	Georgia	2014	MDR	2.2.1
SRR5153272	Georgia	2014	MDR	2.2.1
SRR5153273	Georgia	2014	MDR	2.2.1
SRR5153274	Georgia	2014	Other	2.2.1
SRR5153275	Georgia	2014	MDR	2.2.1
SRR5153276	Georgia	2014	MDR	2.2.1
SRR5153278	Georgia	2014	XDR	2.2.1
SRR5153279	Georgia	2015	Susceptible	2.2.1
SRR5153291	Georgia	2015	MDR	2.2.1
SRR5153303	Georgia	2014	Susceptible	2.2.1
SRR5153307	Georgia	2015	MDR	2.2.1
SRR5153308	Georgia	2015	XDR	2.2.1
SRR5153310	Georgia	2014	MDR	2.2.1
SRR5153311	Georgia	2014	XDR	2.2.1

SRR5153312	Georgia	2014	MDR	2.2.1
SRR5153313	Georgia	2015	MDR	2.2.1
SRR5153314	Georgia	2014	XDR	2.2.1
SRR5153317	Georgia	2014	XDR	2.2.1
SRR5153318	Georgia	2014	Other	2.2.1
SRR5153320	Georgia	2014	MDR	2.2.1
SRR5153321	Georgia	2015	MDR	2.2.1
SRR5153322	Georgia	2014	MDR	2.2.1
SRR5153323	Georgia	2014	MDR	2.2.1
SRR5153326	Georgia	2014	Other	2.2.1
SRR5153327	Georgia	2014	Other	2.2.1
SRR5153328	Georgia	2014	XDR	2.2.1
SRR5153329	Georgia	2014	MDR	2.2.1
SRR5153330	Georgia	2014	MDR	2.2.1
SRR5153331	Georgia	2014	MDR	2.2.1
SRR5153334	Georgia	2014	MDR	2.2.1
SRR5153335	Georgia	2014	MDR	2.2.1
SRR5153336	Georgia	2015	MDR	2.2.1
SRR5153423	Georgia	2014	MDR	2.2.1
SRR5153424	Georgia	2014	Other	2.2.1
SRR5153509	Georgia	2014	MDR	2.2.1
SRR5153595	Georgia	2015	MDR	2.2.1
SRR5153597	Azerbaijan	2015	MDR	2.2.1
SRR5153598	Azerbaijan	2015	MDR	2.2.1
SRR5153599	Azerbaijan	2016	MDR	2.2.1
SRR5153600	Azerbaijan	2015	MDR	2.2.1
SRR5153601	Georgia	2015	MDR	2.2.1
SRR5153602	Georgia	2015	MDR	2.2.1
SRR5153603	Azerbaijan	2015	MDR	2.2.1
SRR5153605	Azerbaijan	2015	MDR	2.2.1
SRR5153606	Azerbaijan	2015	Other	2.2.1
SRR5153608	Azerbaijan	2015	MDR	2.2.1
SRR5153609	Georgia	2014	MDR	2.2.1
SRR5153611	Georgia	2015	MDR	2.2.1
SRR5153612	Azerbaijan	2015	XDR	2.2.1
SRR5153614	Georgia	2015	MDR	2.2.1
SRR5153616	Georgia	2015	MDR	2.2.1
SRR5153617	Azerbaijan	2015	XDR	2.2.1
SRR5153618	Azerbaijan	2015	MDR	2.2.1
SRR5153619	Georgia	2015	MDR	2.2.1
SRR5153620	Azerbaijan	2015	MDR	2.2.1
SRR5153621	Georgia	2015	MDR	2.2.1
SRR5153622	Azerbaijan	2015	MDR	2.2.1
SRR5153709	Azerbaijan	2015	MDR	2.2.1
SRR5153710	Azerbaijan	2015	MDR	2.2.1
SRR5153711	Azerbaijan	2016	MDR	2.2.1
SRR5153712	Azerbaijan	2016	MDR	2.2.1
SRR5153713	Azerbaijan	2016	MDR	2.2.1
SRR5153716	Azerbaijan	2015	MDR	2.2.1
SRR5153718	Azerbaijan	2016	Susceptible	2.2.1
SRR5153720	Azerbaijan	2015	MDR	2.2.1
SRR5153721	Azerbaijan	2015	MDR	2.2.1
SRR5153722	Azerbaijan	2015	MDR	2.2.1
SRR5153723	Azerbaijan	2015	MDR	2.2.1
SRR5153808	Azerbaijan	2016	XDR	2.2.1
SRR5153809	Azerbaijan	2015	XDR	2.2.1
SRR5153812	Azerbaijan	2016	MDR	2.2.1
SRR5153814	Azerbaijan	2015	MDR	2.2.1
SRR5153815	Azerbaijan	2016	MDR	2.2.1
SRR5153816	Moldova	2015	MDR	2.2.1
SRR5153818	Moldova	2012	Susceptible	2.2.1

SRR5153819	Moldova	2015	MDR	2.2.1
SRR5153820	Azerbaijan	2016	MDR	2.2.1
SRR5153825	Azerbaijan	2015	XDR	2.2.1
SRR5153826	Azerbaijan	2015	MDR	2.2.1
SRR5153829	Azerbaijan	2015	MDR	2.2.1
SRR5153830	Moldova	2015	MDR	2.2.1
SRR5153831	Moldova	2015	Other	2.2.1
SRR5153833	Moldova	2012	Susceptible	2.2.1
SRR5153834	Moldova	2016	MDR	2.2.1
SRR5153837	Moldova	2015	MDR	2.2.1
SRR5153841	Moldova	2015	MDR	2.2.1
SRR5153843	Moldova	2009	Susceptible	2.2.1
SRR5153845	Moldova	2012	Susceptible	2.2.1
SRR5153852	Moldova	2015	MDR	2.2.1
SRR5153853	Moldova	2015	MDR	2.2.1
SRR5153856	Moldova	2016	MDR	2.2.1
SRR5153864	Moldova	2016	MDR	2.2.1
SRR5153883	Moldova	2015	MDR	2.2.1
SRR5153884	Moldova	2015	Susceptible	2.2.1
SRR5153886	Moldova	2016	MDR	2.2.1
SRR5153900	Moldova	2009	Susceptible	2.2.1
SRR5153904	Moldova	2015	MDR	2.2.1
SRR5153905	Moldova	2009	MDR	2.2.1
SRR5153908	Moldova	2016	MDR	2.2.1
SRR5153911	Moldova	2015	MDR	2.2.1
SRR5153912	Moldova	2015	MDR	2.2.1
SRR5153916	Moldova	2015	Other	2.2.1
SRR5153919	Moldova	2015	MDR	2.2.1
SRR5153923	Moldova	2015	MDR	2.2.1
SRR5153924	Moldova	2014	MDR	2.2.1
SRR5153925	Moldova	2009	MDR	2.2.1
SRR5153926	Moldova	2015	MDR	2.2.1
SRR5153927	Moldova	2015	Susceptible	2.2.1
SRR5153928	Moldova	2015	MDR	2.2.1
SRR5153930	Moldova	2012	MDR	2.2.1
SRR5306022	Myanmar	2016	MDR	2.2.1
SRR5314268	India	01-December-2011	MDR	2.2.1
SRR5535777	Peru	missing	Susceptible	2.2.1
SRR5818573	Djibouti	2014/2015	Susceptible	2.2.1
SRR5818641	Djibouti	2014/2015	MDR	2.2.1
SRR611415	NA	NA	MDR	2.2.1
SRR611416	NA	NA	MDR	2.2.1
SRR611417	NA	NA	MDR	2.2.1
SRR611421	NA	NA	MDR	2.2.1
SRR611423	NA	NA	Other	2.2.1
SRR611425	NA	NA	Susceptible	2.2.1
SRR611426	NA	NA	MDR	2.2.1
SRR671719	NA	NA	MDR	2.2.1
SRR671720	NA	NA	MDR	2.2.1
SRR671721	NA	NA	MDR	2.2.1
SRR671722	NA	NA	MDR	2.2.1
SRR671723	NA	NA	Other	2.2.1
SRR671724	NA	NA	MDR	2.2.1
SRR671726	NA	NA	Other	2.2.1
SRR671727	NA	NA	Other	2.2.1
SRR671728	NA	NA	Other	2.2.1
SRR671729	NA	NA	Susceptible	2.2.1
SRR671730	NA	NA	Susceptible	2.2.1
SRR671731	NA	NA	MDR	2.2.1
SRR671732	NA	NA	MDR	2.2.1
SRR671733	NA	NA	MDR	2.2.1

SRR671734	NA	NA	MDR	2.2.1
SRR671735	NA	NA	MDR	2.2.1
SRR671736	NA	NA	MDR	2.2.1
SRR671737	NA	NA	Other	2.2.1
SRR671738	NA	NA	MDR	2.2.1
SRR671739	NA	NA	MDR	2.2.1
SRR671742	NA	NA	MDR	2.2.1
SRR671743	NA	NA	MDR	2.2.1
SRR671744	NA	NA	MDR	2.2.1
SRR671745	NA	NA	MDR	2.2.1
SRR671746	NA	NA	MDR	2.2.1
SRR671747	NA	NA	Susceptible	2.2.1
SRR671748	NA	NA	MDR	2.2.1
SRR671752	NA	NA	MDR	2.2.1
SRR671753	NA	NA	MDR	2.2.2
SRR671754	NA	NA	MDR	2.2.1
SRR671755	NA	NA	MDR	2.2.1
SRR671756	NA	NA	Other	2.2.1
SRR671758	NA	NA	Other	2.2.1.1
SRR671759	NA	NA	XDR	2.2.1
SRR671761	NA	NA	XDR	2.2.1.1
SRR671765	NA	NA	MDR	2.2.1
SRR671770	NA	NA	MDR	2.2.1
SRR671771	NA	NA	MDR	2.2.1
SRR671776	NA	NA	Susceptible	2.2.1
SRR671777	NA	NA	Susceptible	2.2.1
SRR671779	NA	NA	XDR	2.2.1
SRR671780	NA	NA	Other	2.2.1
SRR671781	NA	NA	XDR	2.2.1
SRR671783	NA	NA	Other	2.2.1
SRR671784	NA	NA	Other	2.2.1
SRR671785	NA	NA	MDR	2.2.1
SRR671786	NA	NA	Susceptible	2.2.1.1
SRR671787	NA	NA	MDR	2.2.1
SRR671788	NA	NA	Susceptible	2.2.1
SRR671789	NA	NA	MDR	2.2.1
SRR671791	NA	NA	MDR	2.2.1
SRR671792	NA	NA	Susceptible	2.2.1
SRR671793	NA	NA	Susceptible	2.2.1
SRR671794	NA	NA	XDR	2.2.1
SRR671795	NA	NA	MDR	2.2.1
SRR671796	NA	NA	MDR	2.2.1
SRR671797	NA	NA	MDR	2.2.2
SRR671798	NA	NA	MDR	2.2.1
SRR671799	NA	NA	XDR	2.2.1
SRR671800	NA	NA	Susceptible	2.2.1
SRR671801	NA	NA	MDR	2.2.1
SRR671802	NA	NA	Susceptible	2.2.1
SRR671803	NA	NA	Susceptible	2.2.1
SRR671804	NA	NA	MDR	2.2.2
SRR671805	NA	NA	Susceptible	2.2.1
SRR671806	NA	NA	MDR	2.2.1
SRR671807	NA	NA	MDR	2.2.1
SRR671808	NA	NA	Susceptible	2.2.1
SRR671809	NA	NA	MDR	2.2.1
SRR671810	NA	NA	Other	2.2.1
SRR671811	NA	NA	MDR	2.2.1
SRR671812	NA	NA	MDR	2.2.1
SRR671813	NA	NA	Other	2.2.2
SRR671814	NA	NA	Susceptible	2.2.1
SRR671815	NA	NA	MDR	2.2.1

SRR671816	NA	NA	MDR	2.2.1
SRR671817	NA	NA	MDR	2.2.1
SRR671818	NA	NA	MDR	2.2.1
SRR671819	NA	NA	MDR	2.2.1
SRR671820	NA	NA	MDR	2.2.1
SRR671821	NA	NA	MDR	2.2.1
SRR671822	NA	NA	MDR	2.2.1
SRR671823	NA	NA	Susceptible	2.2.1
SRR671824	NA	NA	MDR	2.2.2
SRR671825	NA	NA	MDR	2.2.1
SRR671826	NA	NA	MDR	2.2.1
SRR671827	NA	NA	Susceptible	2.2.1
SRR671828	NA	NA	MDR	2.2.1
SRR671829	NA	NA	MDR	2.2.1
SRR671830	NA	NA	Susceptible	2.2.1.1
SRR671834	NA	NA	MDR	2.2.1
SRR671836	NA	NA	Other	2.2.1
SRR671837	NA	NA	MDR	2.2.1
SRR671839	NA	NA	MDR	2.2.1
SRR671840	NA	NA	MDR	2.2.1
SRR671841	NA	NA	MDR	2.2.1.1
SRR671842	NA	NA	Other	2.2.1
SRR671843	NA	NA	MDR	2.2.1
SRR671844	NA	NA	Other	2.2.1
SRR671845	NA	NA	Other	2.2.1
SRR671847	NA	NA	Susceptible	2.2.1
SRR671848	NA	NA	MDR	2.2.1
SRR671849	NA	NA	Other	2.2.1
SRR671851	NA	NA	Other	2.2.1
SRR671852	NA	NA	MDR	2.2.1
SRR671854	NA	NA	Other	2.2.1
SRR671855	NA	NA	MDR	2.2.2
SRR671856	NA	NA	Susceptible	2.2.1
SRR671857	NA	NA	MDR	2.2.2
SRR671858	NA	NA	Susceptible	2.2.1
SRR671863	NA	NA	MDR	2.2.1
SRR671864	NA	NA	MDR	2.2.1
SRR671865	NA	NA	MDR	2.2.1
SRR671866	NA	NA	MDR	2.2.1
SRR671867	NA	NA	MDR	2.2.1
SRR671869	NA	NA	MDR	2.2.1
SRR671870	NA	NA	Susceptible	2.2.1
SRR671871	NA	NA	Susceptible	2.2.1
SRR671875	NA	NA	Susceptible	2.2.1
SRR671876	NA	NA	Susceptible	2.2.1
SRR671877	NA	NA	Susceptible	2.2.1
SRR671879	NA	NA	Susceptible	2.2.1
SRR786503	NA	NA	Other	2.2.1
SRR786667	NA	NA	MDR	2.2.1
SRR921502	NA	NA	Susceptible	2

NA - not available

REFERENCES

1. Coll F, McNerney R, Guerra-Assuncao JA, Glynn JR, Perdigao J, Viveiros M, et al. A robust SNP barcode for typing Mycobacterium tuberculosis complex strains. Nat Commun. 2014;5:4812.

2. Coll F, McNerney R, Preston MD, Guerra-Assuncao JA, Warry A, Hill-Cawthorne G, et al. Rapid determination of anti-tuberculosis drug resistance from whole-genome sequences. Genome Med. 2015;7(1):51.
